# FAP adipogenesis drives early spinal muscle loss in dysferlinopathy and reveals a therapeutic target

**DOI:** 10.1038/s44321-026-00514-5

**Published:** 2026-10-12

**Authors:** Young Jae Moon, Nikki McCormack, Thomas Diehn, Stephen Lin, Katia Staedler, Surajit Bhattacharya, Daniel C Bittel, Paul C Wang, Marshall Hogarth, Jong-Sung Oh, Tae Sik Goh, Ji Hong Min, Tanya Stojkovic, Isabelle Ackermann-Bonan, Jyoti K Jaiswal

**Affiliations:** 1 Center for Precision Medicine and Genomics Research, Children’s National Research Institute, Children’s National Hospital, 7144 13th Pl NW, Washington, DC, USA; 2 Departments of Biochemistry and Orthopaedic Surgery, Research Institute of Clinical Medicine of Jeonbuk National University – Biomedical Research Institute of Jeonbuk National University Hospital, Jeonbuk National University Medical School, Jeonju, Republic of Korea; 3 Institute for Medical Science of Jeonbuk National University Medical School, Jeonju, Republic of Korea; 4 Molecular Imaging Laboratory, Department of Radiology, Howard University, Washington, DC, USA; 5 Sorbonne Université, Service de Neuromyologie, Centre de référence Nord-Est-Ile-de-France, Institut de Myologie, Hôpital Pitié-Salpêtrière, 47-83 boulevard de l’Hôpital 75651 Paris Cedex 13, Paris, France; 6 Department of Neurology, Neurocenter of Southern Switzerland, Ente Ospedaliero Cantonale, Lugano, Switzerland; 7 Department of Pediatrics, The George Washington University School of Medicine and Health Sciences, Washington, DC, USA; 8 Department of Orthopaedic Surgery, Jeonbuk National University Hospital, Jeonju, Republic of Korea; 9 Department of Orthopaedic Surgery, Biomedical Research Institute, Pusan National University School of Medicine, Busan, Republic of Korea; 10 Department of Rehabilitation Medicine, Research Institute for Convergence of Biomedical Science and Technology, Pusan National University Yangsan Hospital, Yangsan, Republic of Korea

**Keywords:** Genetics, Gene Therapy & Genetic Disease, Musculoskeletal System

## Abstract

Dysferlinopathy (Limb Girdle Muscular Dystrophy R2/LGMD2B) causes progressive weakness of limb-girdle muscles. Although gait abnormalities are common, the contribution of spinal muscles to disease symptoms remains poorly understood. Using cross-sectional MRI in patients and longitudinal analyses in a dysferlinopathic mouse model, we identify early-onset, progressive adipogenic degeneration of spinal muscles as a previously unrecognized hallmark of dysferlinopathy. Multiple spinal muscles, including the iliocostalis, longissimus, and spinalis, are affected, with degeneration preceding pathology in limb muscles. In mice, early spinal muscle loss is associated with altered posture and gait, implicating axial muscle degeneration as a functional driver of disease. Mechanistically, spinal muscle degeneration is accompanied by an intrinsic increase in adipogenic differentiation of spinal fibroadipogenic progenitors (FAPs). Transcriptomic profiling implicated HDAC and Wnt/ß-catenin signaling as the main regulators of FAP adipogenic shift. Pharmacological inhibition of either pathway suppressed adipogenic differentiation of dysferlinopathic FAPs in vitro, with in vivo inhibition of GSK3 reducing adipogenic degeneration in spinal and injured limb muscles. These findings establish early spinal muscle degeneration as a key pathological feature of dysferlinopathy and identify GSK3 signaling as a promising therapeutic target.

## Abstract (web summary)

Early degeneration of spinal muscles, rather than limb muscles, emerges as a key driver of gait dysfunction in dysferlinopathy, revealing axial muscle pathology as a previously overlooked disease feature and identifying GSK3 inhibition as a promising therapeutic strategy.

## Graphical abstract

**Figure:**
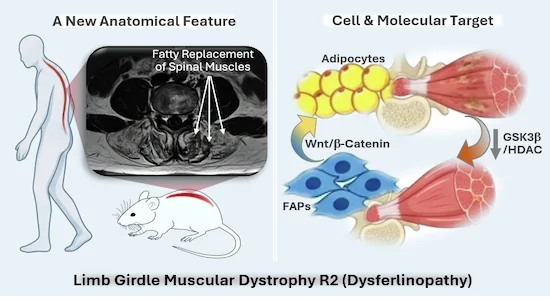

## Introduction

Limb Girdle Muscular Dystrophy (LGMD) refers to a group of disorders characterized by progressive skeletal muscle weakness that initially affects the limb and girdle muscles (Angelini, 2020; Straub et al, 2018). The disease presents a wide range of muscle involvement, varying in severity, onset, and progression. LGMD can range from severe forms with early onset and rapid progression to milder forms with late or slow onset. The classification of LGMD involves a proximal or distal-proximal muscle presentation, myofiber degeneration, elevated creatine kinase (CK) levels, and evidence of fibro-fatty degeneration notable through magnetic resonance imaging (MRI) and by histopathological examination of the affected muscles. The degree of muscle loss and weakness varies according to genetic factors and is heterogeneous among individuals (Angelini, 2020).

LGMD2B/R2, or dysferlinopathy, is an autosomal recessive subtype caused by mutations in the *DYSF* gene that lead to the reduction or absence of the muscle protein dysferlin (Bashir et al, 1998; Liu et al, 1998). Dysferlin is a transmembrane protein present at the sarcolemma, T-tubules, and cytoplasmic vesicles that facilitates calcium-dependent repair of plasma membrane injury and maintenance of calcium homeostasis (Bansal et al, 2003; Defour et al, 2014; Quinn et al, 2024). Consequently, the absence of dysferlin inhibits repair and survival of the injured myofibers, resulting in progressive loss of the myofibers and eventual fibrofatty replacement of muscles, typically starting in early adulthood and progressively causing loss of limb and girdle muscles (Diaz-Manera et al, 2018; Selcen et al, 2001). Patients affected by dysferlinopathy are diagnosed on the basis of muscle weakness, elevated serum CK level, lack of dysferlin protein in skeletal muscle or peripheral blood monocytes, and/or confirmed genetic test of biallelic pathogenic mutations in the *DYSF* gene (Bouchard and Tremblay, 2023; Gallardo et al, 2001; Ho et al, 2002; Meznaric et al, 2011). Muscle MRI analysis of dysferlinopathic patients shows fatty replacement of posterior thigh and leg muscles, which can extend to other limb and trunk muscles and is associated with loss of these muscles (Llanso et al, 2023). However, these human studies do not establish if fatty replacement is the cause of the disease or a consequence of myofiber loss due to poor repair of damaged dysferlinopathic myofiber. The BLA/J mouse model of dysferlinopathy demonstrates late-onset limb muscle loss attributable to adipogenesis, with cellular pathology closely resembling that observed in dysferlinopathic patients. This model provides an alternative to human studies by enabling longitudinal monitoring of limb and trunk muscles throughout disease onset and progression (Hornsey et al, 2013; Nagy et al, 2017).

Despite significant efforts to understand dysferlinopathy, no therapies are available for this disease. While chronic muscle inflammation is a feature of this disease, use of anti-inflammatory corticosteroids is ineffective for dysferlinopathy (Walter et al, 2013). While none of the therapies have been approved for clinical use in human, use of the dysferlinopathic mouse model has helped identify potential therapeutic avenues (Bouchard and Tremblay, 2023; Rawls et al, 2023). In one such example, Vamorolone, a modified corticosteroid approved for treating Duchenne muscular dystrophy (DMD), has shown promise in our preclinical studies using a dysferlinopathic mouse model (Sreetama et al, 2018). Other targets to prevent muscle loss in dysferlinopathy include restorative gene therapy, drugs that stabilize mutant dysferlin protein, muscle cell membrane, regulate sphingomyelin, or limit adipogenic fate of fibroadipogenic progenitors (FAPs) (Bittel et al, 2022; Gushchina et al, 2017; Hogarth et al, 2019; Potter et al, 2018; Pryadkina et al, 2015; Tominaga et al, 2022).

Here, we have performed muscle MRI-based comparisons of anatomical changes in dysferlinopathic patients and mice in addition to mechanistic and drug studies. Results of these studies identify adipogenic degeneration of spinal muscles as a unifying dystrophic feature of both human and mice affected by dysferlinopathy. Cross-sectional assessments in patients showed a correlation of muscle MRI outcomes with disease, while longitudinal muscle MRI assessment in the mouse model revealed spinal muscle degeneration as one of the earliest signs of skeletal muscle loss due to dysferlinopathy. We find that this contributes to the characteristic gait abnormalities seen in mice and patients. Further, we find that at the cellular level, paraspinal muscle loss is linked to impaired repair of dysferlin-deficient myofibers, which triggers excess inflammation, and FAP accumulation and adipogenic differentiation. By transcriptomic analysis of healthy and dysferlinopathic FAPs and in vitro testing of potential therapeutic targets using paraspinal muscle FAPs, we identified and tested potential drug-based therapies to treat adipogenic loss of spinal muscle in dysferlinopathy.

## Results

### Fatty replacement of paraspinal muscles is a feature of dysferlinopathy patients

**Figure 1:**
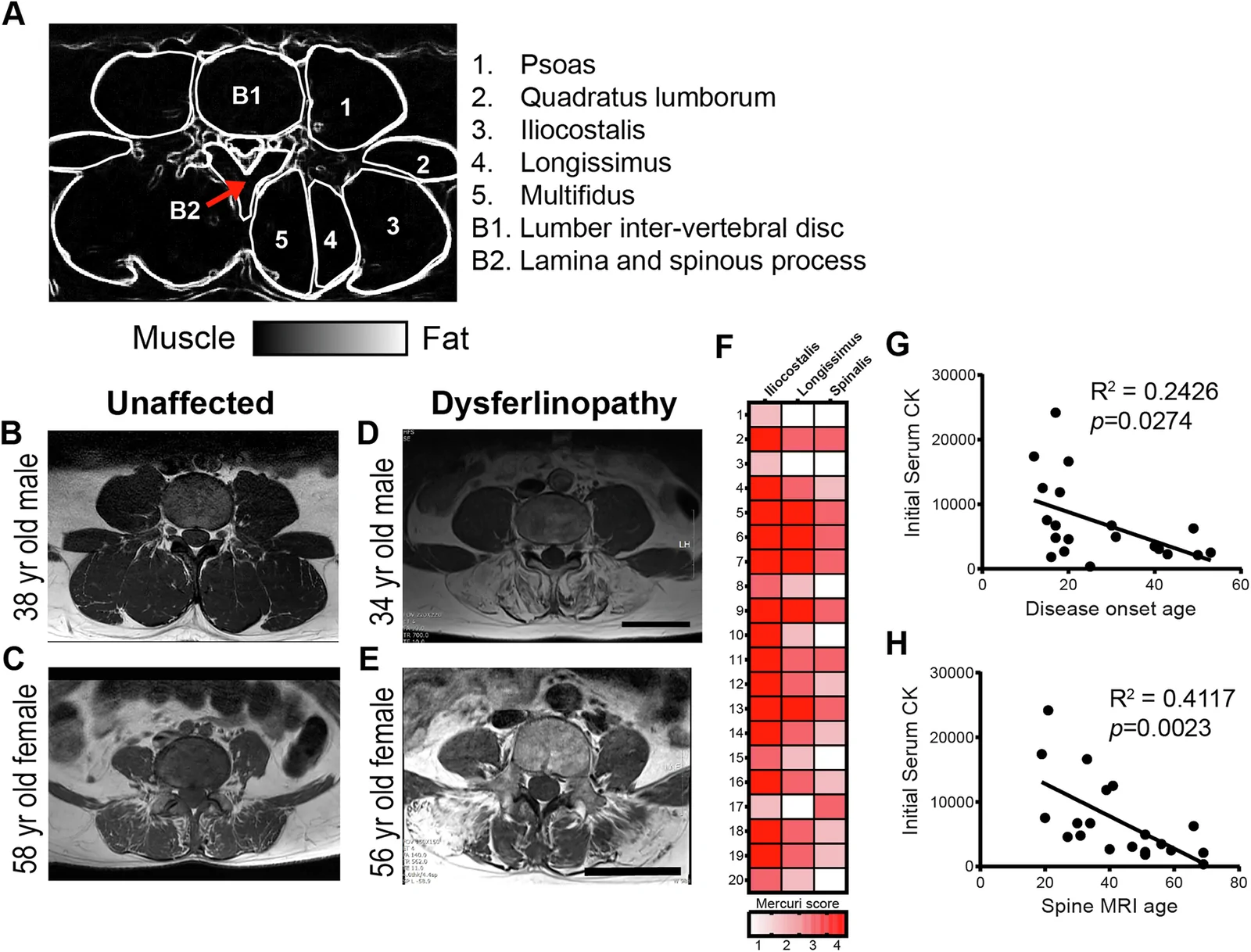
MRI analysis of paraspinal muscle degeneration in dysferlinopathic patients. (**A**) Schematic showing the anatomical location of muscles around the human lumbar spine. (**B**–**E**) T1-weighted axial MRI images showing the disc space of L3-4 or the superior endplate of L4 of (**B**, **C**) Unaffected subjects: (**B**) 38-year-old male, (**C**) 58-year-old female. (**D**, **E**) Age- and sex-matched Dysferlinopathy patients. (**D**) 34-year-old male (ID #2), this MRI scan was conducted four years after symptom onset. (**E**) 56-year-old female (ID #4), this MRI scan was sixteen years after symptom onset. Scale bar = 30 mm. (**F**) Heatmap showing the degree of fatty change observed on MRI T1-weighted images of the iliocostalis, longissimus, and spinalis, which constitute the spinal muscles of 20 dysferlinopathy patients. (**G**, **H**) Scatterplots of initial serum CK level (IU/L) and disease onset age (**G**) or age at time of spine MRI (**H**). Statistical analyses used simple linear regression with *P* values as noted. The coefficient of determination was used to compare the association between initial serum CK level (IU/L) and disease onset age or age at time of spine MRI (*n* = 20). Source data are available online for this figure.

**Figure EV1:**
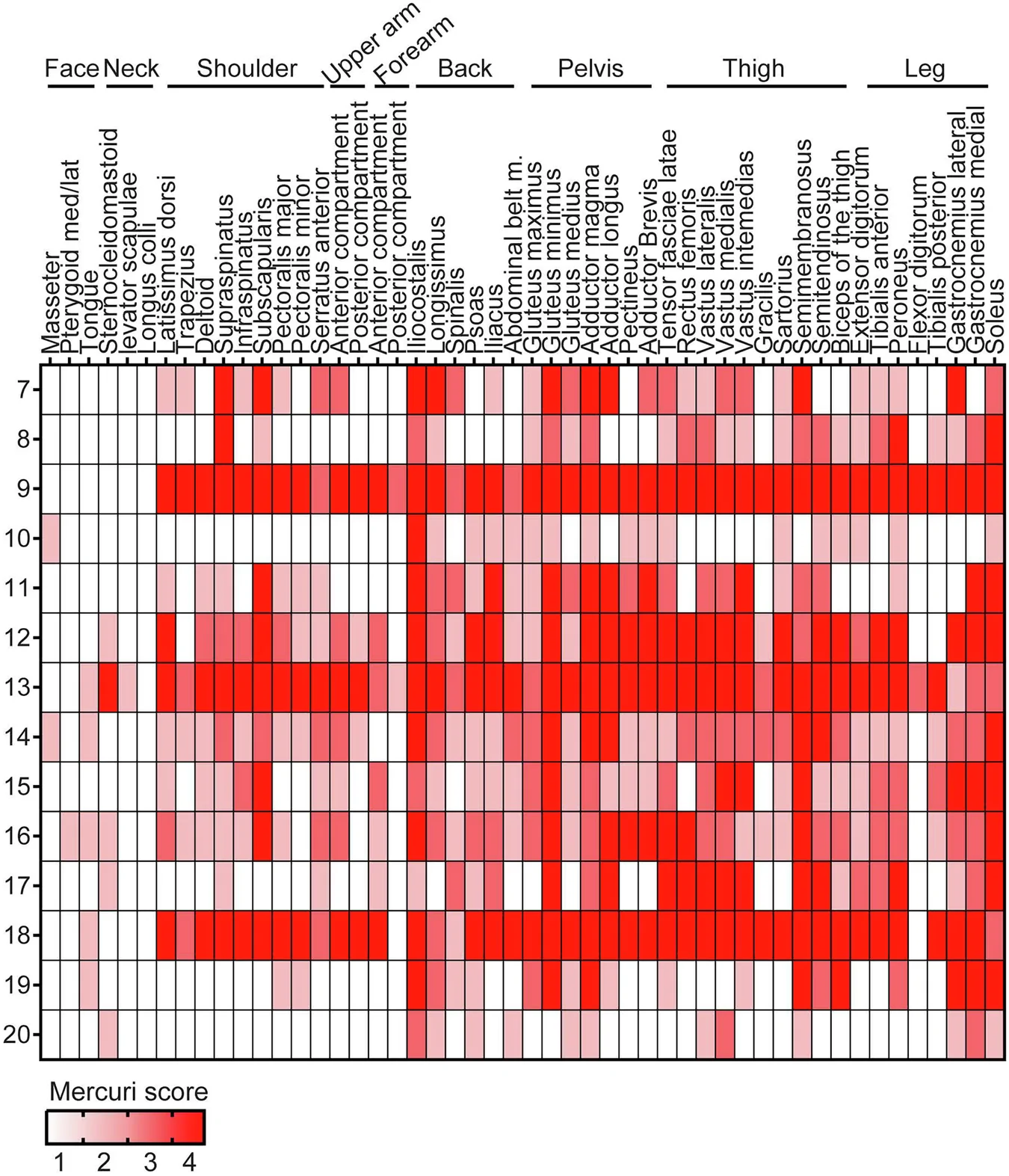
Heterogeneous manifestation of muscle loss in the dysferlinopathic patient cohort. Heat map showing the degree of fatty change observed on MRI T1-weighted images of the various body muscles imaged in the dysferlinopathic patient cohort that underwent high-resolution whole-body MRI. The numbers denote the patient ID as listed in Table 1.

To identify changes in axial muscles in patients with dysferlinopathy, we retrospectively identified a cohort of dysferlinopathic patients who underwent muscle MRI at two different locations (South Korea and France). This resulted in data from 20 dysferlinopathic patients with biallelic mutations in the dysferlin gene confirmed by next-generation sequencing (NGS) (Table 1). All patients suffered from progressive limb-girdle muscle weakness and had significantly elevated serum creatine kinase (CK) levels (Table 1). Based on the clinical features, these patients were diagnosed as either (A) distal myopathy (Miyoshi type), (B) Limb girdle myopathy (LGMD type), or (C) CK elevation with/without exercise intolerance (Table 1). The T1-weighted lumbar spine MRI scans from patients were analyzed for individual spinal muscles (Fig. 1A). This showed that, unlike the images of unaffected controls, dysferlinopathic patients exhibit high T1 signal intensity of erector spinae muscles, indicating fatty replacement of these muscles in patients (Fig. 1B–E). While these images showed a marked degeneration of several spinal muscles in patients, MRI images of the adjacent pelvic girdle muscles that support hip and back flexion (psoas and quadratus lumborum) as well as limb muscles remained unaffected (Figs. 1F and EV1). Detailed analysis of the T1 images showed that the lower back muscles were more affected than the lower dorsal muscles, while the upper dorsal and cranial muscles were the least affected. Degeneration of the spinal muscle manifested as iliocostalis > longissimus > spinalis (Fig. 1F). Use of serum CK as an indicator of disease severity identified that a higher serum CK level was associated with an earlier age of disease onset and age at which spinal MRI needed to be performed (Fig. 1G,H). The extent and severity of spinal muscle degeneration vary among patients and among different spinal muscles. For example, in patient #4, high signal intensity was observed only in the iliocostalis muscle, not in multifidus, longissimus, and iliocostalis, which make up the erector spinae muscles (Fig. 1E). However, irrespective of how widespread fatty degeneration was, the extent of signal in the spinal muscles was greater than in the girdle muscles. Together, these results demonstrate degeneration of the erector spinae muscles, with the iliocostalis muscles being the first or the most severely affected spinal muscle.

**Table 1:** Clinical and genetic features of the patients examined for this study.

ID	Sex	Race	*Dysf* variant	Predicted protein change	Diagnosis	Clinical status	Serum CK (#)	Age of Onset^#^	Age at MRI^#^
1	F	Asian	Intron 13: c.1284+2 T > C, Exon 24: c.2494 C > T	Splicing error, p.Q832*	A	Gradual progression; Trouble climbing stairs at 15	7522 (15)	15	20^†^
2	M	Asian	Intron 13: c.1284+2 T > C, Exon 16: c.1464delT	Splicing error, p. G489Efs*4	A	Gradual progression; Trouble climbing stairs at 30	6703 (30)	30	34^†^
3	F	Asian	Exon 11: c.965 T > C, Exon 12: c.1129 C > T	p.L322P, p.R377*	A	Gradual progression; Ambulatory, polymyalgia	2264 (43)	43	51^†^
4	F	Asian	Exon 28: c.2997 G > T, Exon 12: c.1169 A > G	p.W999C, p. D390G	B	Gradual progression; Trouble climbing stairs at 40	3492 (40)	40	56^†^
5	M	Asian	Intron 13: c.1284+2 T > C, Exon 51: c.5713 C > T	Splicing error, p. R1905*	B	Gradual progression; Wheelchair bound at 58.	2136 (50)	50	69^†^
6	M	Asian	Intron 6: c.663+1 G > C, Exon 28: c.2974 T > C	Splicing error, p. W992R	A	Gradual progression; Trouble climbing stairs at 18, Wheelchair bound at 45.	352 (69)	25	69^†^
7	M	Caucasian	Exon 19: c.1663 C > T Exon 11: c.1004 G > C	Exon 19: p. Arg555Trp Exon 11: p. Gly335Ala	C	Isolated hyperCKemia; Camptocormia (57 y); Late proximal lower limb involvement; Ambulatory with a walker	6253 (49)	49	66^†^, 69, 70, 71, 72, 73
8	F	Caucasian	Intron 8: c.855+1delG Exon 50: c.5594delG	Intron 8: mRNA-decay Exon 50: p. Gly1865AlafsX101	A	Gradual proximodistal progression; Wheelchair bound at 30	17393 (15)	12	19^†^, 20, 21, 22
9	F	Caucasian	Exon 9: c.896 G > A Exon 12: c.1168 G > A	Exon 9: p. Gly299Glu Exon 12: p. Asp390Asn	A	Gradual proximodistal progression; Wheelchair bound at 43.	12500 (22)	14	^†^41, 42, 43, 44
10	M	Arabic	Exon 47: c.5303 G > A	Exon 47: p. Arg1768Gln	C	HyperCKemia with exercise intolerance; No weakness; Ambulant	2668 (34)	19	^†^40, 42, 43
11	F	Caucasian	Exon 52: c.5871_5872delGT Exon 7: c.757 C > T	Exon 52: p. Ser1958ProfsX3 Exon 7: p. Arg253Trp	C	Initial hyperCKemia with exercise intolerance; Distal myopathy (46 y), then proximo-distal involvement; Wheelchair bound at 51	4938 (43)	31	51^†^, 52, 54
12	F	Arabic	Exon1: c20T>C Exon 26: c.2810 C > T	Exon1: p. Leu7Pro Exon 26: p. Thr937Ile	A	Gradual proximo-distal weakness; Ambulatory with a crutch	6710 (24)	17	30^†^, 31
13	M	Caucasian	Exon 46 (IVS46): c.5200+1 > A	Exon 46 (IVS46): p. Glu1734Glyfs*47	A	Gradual progression; Wheelchair bound at 30	11840 (22)	18	39^†^, 40, 41, 42
14	M	Caucasian	Exon 30: c.3321_3322delinsC	Exon 30: p. Ala1108LeufsX12	B	Gradual progression; Ambulatory	4589 (27)	20	27^†^, 28, 29, 30
15	F	Caucasian	Exon 30: c.3321_3322delinsC	- Exon 30: p. Ala1108LeufsX12	B	Gradual progression; Ambulatory	4805 (28)	17	31^†^, 33, 34
16	M	Arabic	Intron 5: c.457+1dupG	Intron 5: abnormal splicing	A	Gradual progression; Loss of ambulation at 27	24170 (18)	17	21^†^, 22, 23, 24
17	M	Arabic	Exon 19: c.1663 C > T	Exon 19: p. Arg555Trp	A	Gradual progression with proximo-distal involvement; Wheelchair bound at 37	16600 (22)	20	33^†^, 34, 35, 36
18	F	Caucasian	Exon12: c.1177 C > T Exon12: c.1168 G > A	Exon12: p. Gln393X Exon12: p. Asp390Asn	B	Gradual progression; Wheelchair bound at 47	1830 (19)	16	51^†^, 52 53, 54
19	F	Arabic	Exon1: c20T>C in Exon 26: c.2810 C > T	Exon1: p. Leu7Pro Exon 26: p. Thr937Ile	B	Gradual progression; Ambulatory	3057 (47)	41	47^†^
20	F	Caucasian	Exon 1: c.5 T > C Exon 44: c.4856dup	Exon 1: p. Leu2Pro Exon 44: p. Cys1621Leufs*29	C	Initial hyperCKemia with exercise intolerance; LGMD phenotype (59 y) with gradual progression; Walks with a cane	2500 (56)	53	59^†^

*A* Miyoshi myopathy, *B* LGMD, *C* CK elevation, *+/−* exercise intolerance, *CK* creatine kinase, *DYSF* dysferlin, *Hom* homozygous.

*Nonsense mutation

^#^Age in years

^†^Age at which spinal muscle loss was observed

### Fatty degeneration of spinal muscles precedes degeneration of quadriceps in BLA/J mice

**Figure 2:**
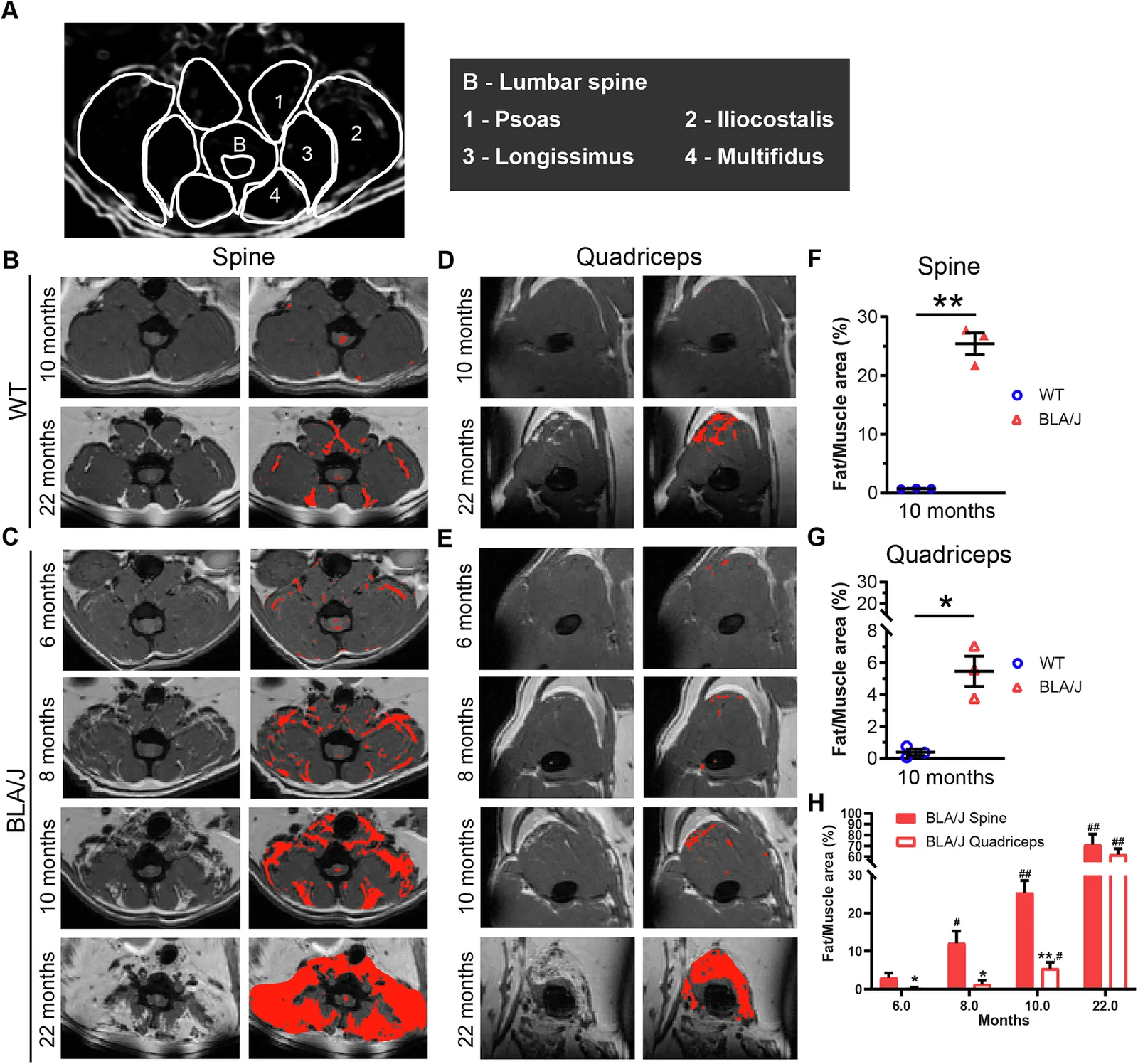
Assessment of progressive fatty degeneration of paraspinal and limb muscle in dysferlinopathic mice by MRI. (**A**) Schematic showing the anatomical location of mouse spinal muscles around the lumbar spine. (**B**, **C**) T1-weighted axial MRI images of WT (**B**) lumbar spine and (**C**) quadriceps muscles without (left panels) and with (right panels) fat saturation from WT mice at 10 months and 22 months of age. (**D**, **E**) T1-weighed axial MRI images without and with fat saturation (as above) for BLA/J mice from (**D**) lumbar spine areas and (**E**) quadriceps muscles from mice at 6-months, 8-months, 10-months, and 22-months of mouse age. Red color indicates the fat fractions. (**F**, **G**) Plots showing quantification of percent fat fraction area from 10-month-old (**F**) WT spine and BLA/J spine. (**G**) WT and BLA/J quadriceps. (**H**) Plot showing the quantification of the percent fat fraction area from the BLA/J spine and quadriceps at the indicated time. *: BLA/J spine vs BLA/J quadriceps, #: vs. 6-month-old BLA/J spine or BLA/J quadriceps in the same group. Scale bar = 2.6 mm (white), and 1.8 mm (yellow). The values are mean ± SD from *n* = 3 mice per group. *P < 0.05, **P < 0.01, ***P < 0.001 by *t* test or two-way ANOVA, Holm–Šídák’s multiple-comparisons test. Source data are available online for this figure.

The observations above suggest dysferlinopathy causes early-onset spinal muscle adipogenic degeneration. However, an alternate possibility is that spinal muscle degeneration in dysferlinopathic patients is a secondary manifestation of limb weakness in these patients, leading to greater axial load on the spine muscles to stabilize the vertical biped posture and walking gait of the patient. If so, we reasoned, this feature will be limited to the patients (biped) and not to the mouse (quadruped) model. To examine this, we performed T1-weighted MR imaging of the spinal muscles using WT and BLA/J mouse model of dysferlinopathy (Fig. 2A). At their symptomatic age (10 months old), BLA/J mice showed a significant increase in the fatty degeneration of their limb and the spinal muscles such that the spinal muscles showing 25-fold greater fatty replacement as compared to fivefold greater degeneration of the limb (quadriceps) muscle of BLA/J mice when compared to the WT mice (Fig. 2B,F,G). To monitor the age of onset of this fatty degeneration, we performed MRI analysis of BLA/J mice at 6, 8, 10, and 22 months of age and compared the results with the WT mice (Fig. 2B–E). This showed fatty degeneration first manifests at the spinal muscles by 6 months of age, when there is little to no fatty MRI-detectable degeneration of the quadriceps muscle (Fig. 2E,H). The adipogenic loss of spinal muscle progressed further by 8, 10, and 22 months, being first detectable in the iliocostalis muscles and progressing to the longissimus and multifidus and the entire spinal muscle cluster (Fig. 2C,F,H). The quadriceps muscle lagged behind the spinal muscle in the onset of fatty degeneration, but followed a similar trajectory, showing increased fatty degeneration detectable in BLA/J by 10 months of age, which progresses throughout the muscle by 22 months of age (Fig. 2E,G,H). The WT mice lacked fatty degeneration of spinal or quadriceps muscles with minimal signs of degeneration even at 22 months of age (Fig. 2B,D). These findings suggest that fatty degeneration of spinal muscles is a common and primary deficit associated with dysferlinopathy, not a consequence of the degeneration of the limb/girdle muscles. Further, it showed that the loss of spinal muscle is not a feature of the biped gait/posture of the human, but a feature of dysferlinopathy.

### Spinal muscle degeneration is linked to spine rigidity and abnormal gait

**Figure 3:**
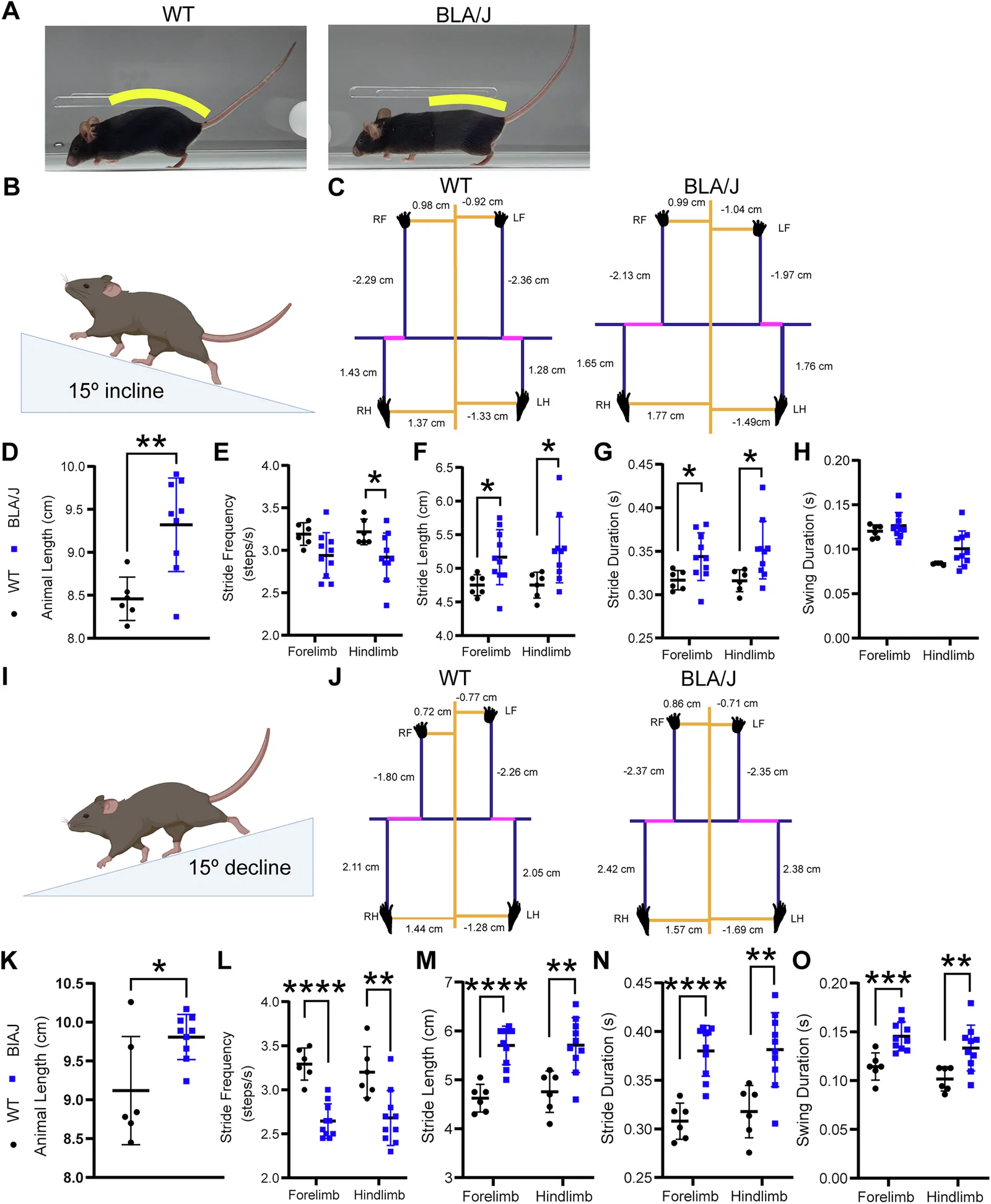
Analysis of posture, locomotion, and gait changes in BLA/J mice. (**A**) Image showing the lateral view of the body posture of 8-month-old mice during locomotion on a treadmill at a speed of 18 cm/s. The yellow line traces the shape of the rear spine. (**B**) Schematic showing mouse running on a treadmill with 15° incline. (**C**) Representative posture plot generated by Digigait software for a mouse walking on a 15° incline treadmill at a speed of 15 cm/s. The golden lines mark the midline and axial distance of the forepaw and hindpaw. The magenta line indicates the difference between the midline distance of the hindlimb and the midline distance of the forelimb. RF right forelimb, LF left forelimb, RH right hindlimb, LH left hindlimb. (**D**–**H**) Plots showing quantification of animal length and gait parameters of BLA/J (*n* = 10) and WT (*n* = 6) mice (8-month-old females) walking on a 15° incline. Results for gait parameters included both left and right sides. Displayed are the (**D**) animal length, (**E**) stride frequency, (**F**) stride length, (**G**) stride duration, and (**H**) swing duration. (**I**) Schematic showing mouse running on a treadmill with 15° decline. (**J**) Representative posture plot similar to (**C**) above, generated for a mouse walking on a 15° decline treadmill at a speed of 15 cm/s. (**K**–**O**) Plots showing quantification of animal length and gait parameters of BLA/J (*n* = 10) and WT (*n* = 6) mice (8-month-old females) walking on a 15° decline. Results for gait parameters included both left and right sides. (**K**) Animal length, (**L**) stride frequency, (**M**) stride length, (**N**) stride duration, and (**O**) swing duration. The values represent mean ± SD. *P < 0.05, **P < 0.01, ***P < 0.001, ****P < 0.0001 by *t* test/Mann–Whitney test. Source data are available online for this figure.

**Figure EV2:**
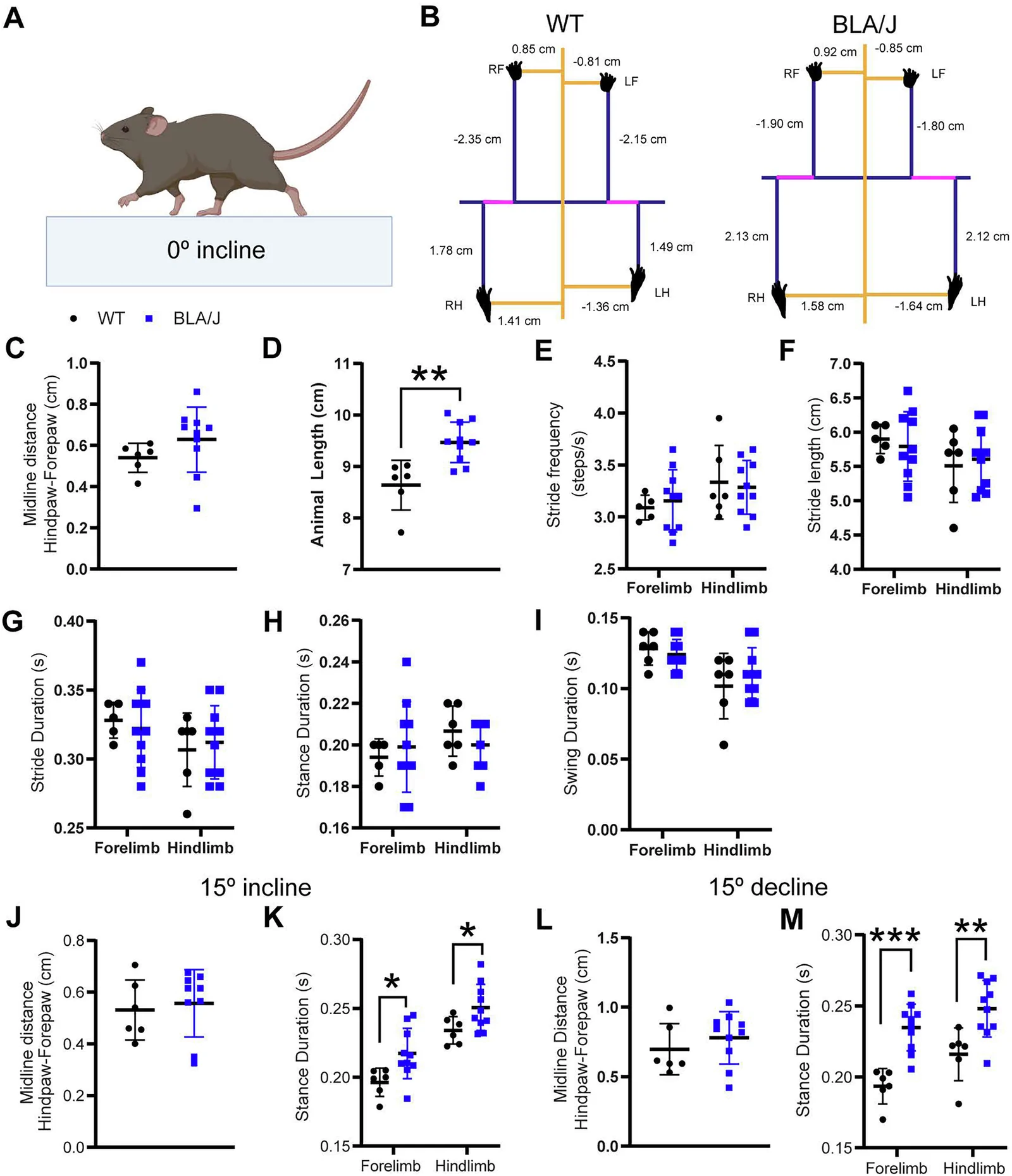
Analysis of dysferlinopathic mouse gait during horizontal locomotion. (**A**) Schematic showing a mouse running on a flat treadmill. (**B**) Representative posture plot generated by Digigait software for a mouse walking on the treadmill at a speed of 18 cm/s. The golden lines mark the midline and axial distance of the forepaw and hindpaw. The magenta line indicates the difference between the midline distance of the hindlimb and the midline distance of the forelimb. RF right forelimb, LF left forelimb, RH right hindlimb, LH left hindlimb. (**C**–**I**) Plots showing quantification of (**C**) midline distance between forepaw and hindpaw, (**D**) animal length, (**E**) stride frequency, (**F**) stride length, (**G**) stride duration, (**H**) stride duration, and (**I**) swing duration. Gait parameters are presented for 8-month-old female BLA/J (*n* = 10) and WT (*n* = 6) mice, with results included for both (left and right) sides. (**J**, **K**) Plots showing (**J**) midline distance, and (**K**) stance duration for BLA/J and WT mice walking on a 15° incline. (**L**, **M**) Plots showing (**L**) midline distance, and (**M**) stance duration from mice walking on a 15° decline. The values are mean ± SD. *P < 0.05, **P < 0.01, ***P < 0.001, ****P < 0.0001 by *T* test (when normally distributed) or Mann–Whitney test.

In the early stages of the disease, the gait pattern of patients with dysferlinopathy is characterized by a normal step length but a wider stride width (Mahjneh et al, 2001). After 10 years of disease, patients manifest a rapid decrease in the swing phase, an increase in hip tilt, and hyperlordosis (Chawla et al, 2025; Mahjneh et al, 2001). To investigate whether adipogenic degeneration of the spinal muscle of BLA/J mice affects their locomotion on a treadmill, we conducted gait analysis. Healthy wild-type (WT) mice typically have a natural spine curvature that creates a slightly hunched posture. In contrast, symptomatic BLA/J mice lost this curvature, resulting in a noticeably flatter posture (Fig. 3A). To quantify these posture changes, we generated posture plots of WT and BLA/J mice walking on inclined (Fig. 3B–H), declined (Fig. 3I–O), and flat (Fig. EV2) treadmills. We quantified the axial and horizontal body lengths marked by vertical gold line for body’s midline length and horizontal gold lines marking the position of the forepaws and hindpaws (Figs. 3C,J and EV2B). In all locomotory conditions, BLA/J mice had a longer body length than WT mice reflecting their flattened posture (Figs. 3D,K and EV2D). Limb positioning relative to the midline (magenta line) remained unchanged, indicating that spinal muscle loss did not affect limb placement in the BLA/J mice (Fig. EV2J,L).

We next assessed gait parameters and found that on both inclined (Fig. 3B–H) and declined treadmills (Fig. 3I–O), BLA/J mice showed increased stride length, stride duration, and stance duration, with mice on the decline treadmill also showing increased swing duration (Figs. 3F–H,M–O and EV2K,M). As the treadmill was maintained at a constant speed (15 cm/s), these alterations in locomotory parameters in BLA/J mice were matched by a concomitant reduction in their forelimb and hindlimb stride frequency. We then measured gait and found that BLA/J mice had longer strides and spent more time in each step phase (stance and stride duration) on both uphill and downhill treadmills. On the downhill treadmill, they also had longer swing times (Figs. 3F–H,M–O and EV2K,M). Since the treadmill speed was fixed at 15 cm/s, these changes meant the mice stepped less often with both their front and back legs. (Fig. 3E,L). The increase in BLA/J body length as compared to the WT mice caused by a flattened spine manifested even during horizontal (0° incline) locomotion (Fig. EV2B,D). However, the locomotory deficit due to limb placement (relative to the midline) was no longer evident during horizontal locomotion (Fig. EV2B,C). Their stride frequency, length, duration, stance, and swing durations were also similar to WT mice during horizontal locomotion (Fig. EV2E–I). The findings show that rigidity and spinal muscle loss in BLA/J mice lead them to adapt during incline and decline locomotion by walking with fewer but longer and slower steps, affecting both forelimb and hindlimb stride frequency. This adjustment helps them maintain mobility and locomotory speed despite structural changes to the spinal muscle. Recognizing such compensatory strategies in humans could help identify early signs of patient adaptations to the disease and inform rehabilitation approaches aimed at delaying functional decline in mobility.

### BLA/J mice show progressive myofiber degeneration and fatty replacement of the spinal muscle

**Figure 4:**
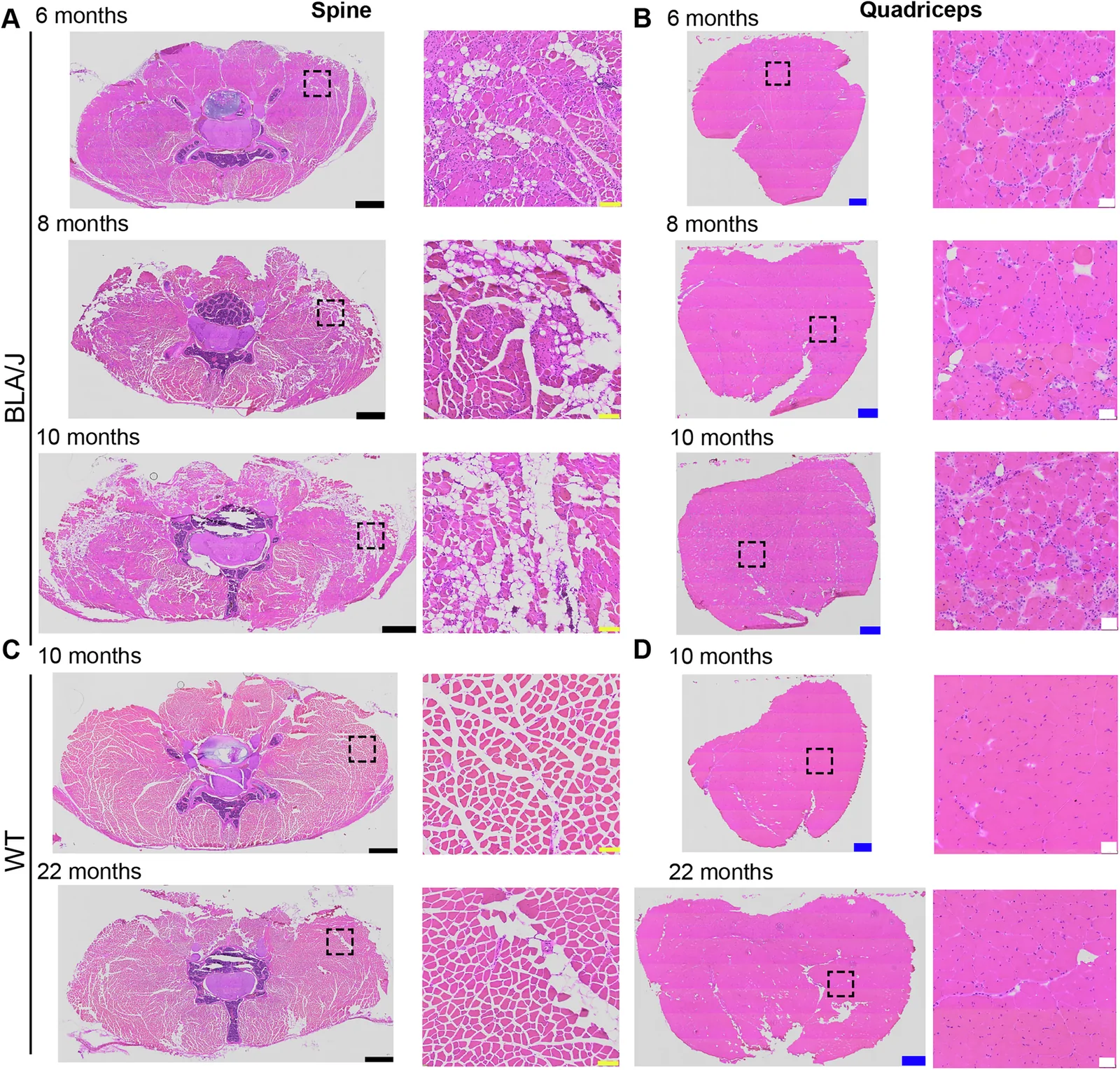
Histological assessment of progressive degeneration of spinal and quadriceps muscles in BLA/J mice. (**A**) Images showing H&E staining of lumbar spine muscles of 6-month, 8-month, and 10-month-old BLA/J mice. (**B**) Images showing H&E staining of quadriceps muscles of 6-month, 8-month, and 10-month-old BLA/J mice. (**C**, **D**) Images showing H&E staining of (**C**) lumbar spine and (**D**) Quadriceps from 10-month, and 22-month-old WT (C57Bl6) mice. The dotted square marks the location of the zoomed-in image presented to the right. Scale bar = 1 mm (black), 500 µm (blue), 100 µm (yellow), and 50 µm (white). Source data are available online for this figure.

While gait modification is an avenue for rehabilitative care of *DYSF*-mutated patients, to gain therapeutic insights into the underlying cellular pathology responsible for spine degeneration, we performed histopathological assessment. We first examined the spinal muscles from BLA/J mice from early to late symptomatic stages (6 months to 10 months of age) and matched the WT control. Hemotoxylin and eosin staining revealed centrally nucleated fibers, immune cell infiltration, and fibrofatty muscle degeneration in BLA/J spinal muscles, as early as 6 months of age (Fig. 4A). This worsens progressively by 10 months of age, with widespread replacement of myofibers with fibrofatty deposits. In comparison to the spine, and similar to our previous studies (Bittel et al, 2022; Hogarth et al, 2019), we found quadriceps were less severe at 6 and 8 months of age, with predominantly centrally nucleated fibers and immune infiltration and minimal fibrofatty replacement of muscle but increased infiltrating immune cells and necrotic fibers by 10 months of age (Fig. 4B). These phenotypes are in contrast to the WT mice, where the spinal and quadriceps muscles show no such extensive histopathological signs of muscle degeneration even at 22 months of age (Fig. 4C,D).

**Figure 5:**
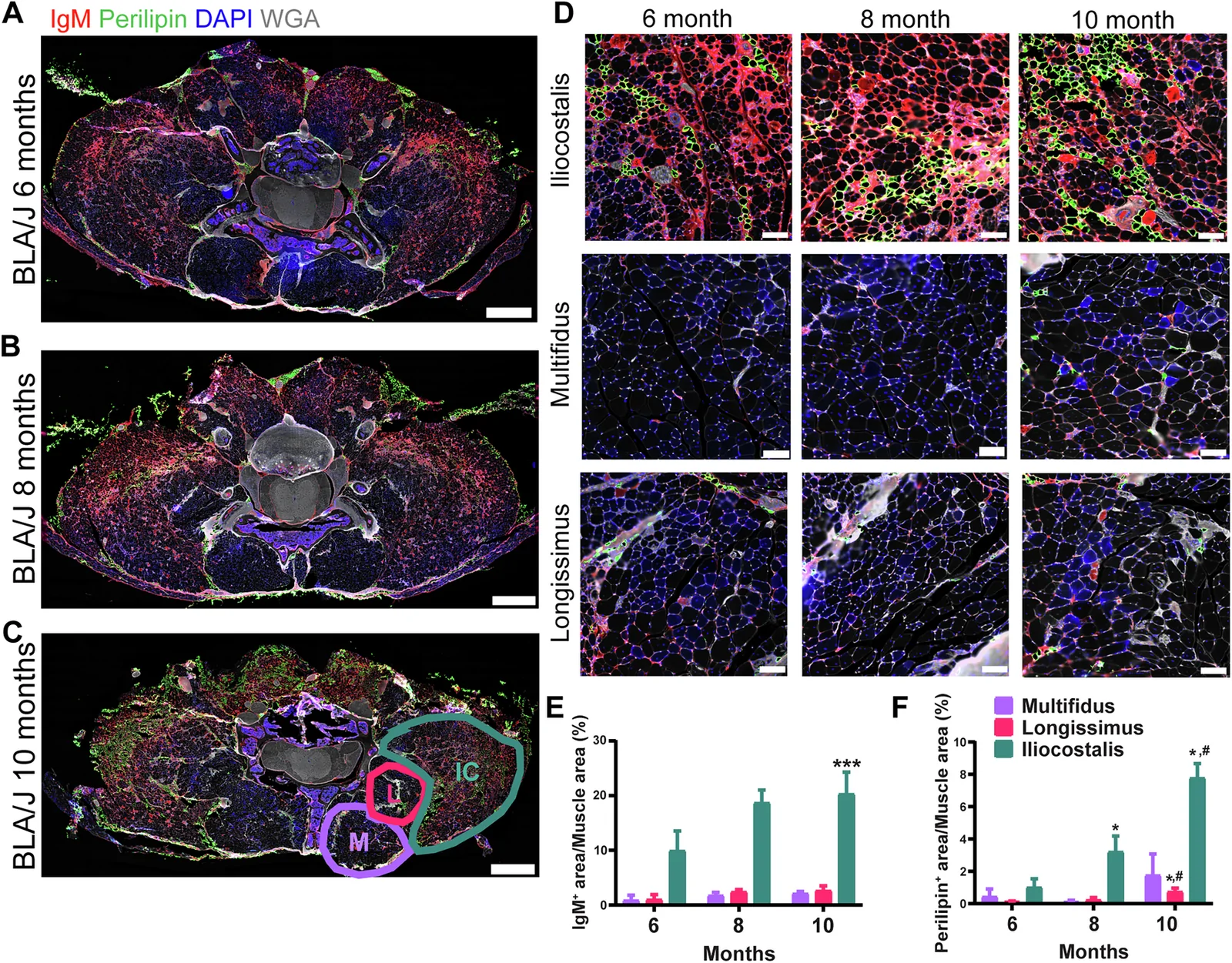
Evidence of myofiber damage, FAP accumulation, and fatty degeneration of spinal muscles. (**A**–**C**) Images showing 6-month, 8-month, and 10-month-old BLA/J spine cross-sections stained to identify leaky/damaged myofibers (IgM) and adipocytes (perilipin) and co-stained with DAPI and WGA to mark the nuclei and tissue matrix. (**D**) Images of zoomed-in areas of the illicostalis, multifidus, and longissimus muscles at 6, 8, and 10 months. (**E**, **F**) Quantification of the percent of the IgM^+^ area (**E**) and perilipin^+^ area (**F**) in multifidus, longissimus, and iliocostalis muscle from the BLA/J spine at the indicated ages. The values are mean ± SD from *n* = 3 mice per group. *: vs. 6-month, #: vs. 8-month in the same group. *P < 0.05, **P < 0.01, ***P < 0.001 by two-way ANOVA, Holm–Šídák’s multiple-comparisons test. Scale bars = 1 mm (**A**–**C**), 100 μm (**D**). Source data are available online for this figure.

Immune infiltration is a physiological response of muscle damage, which guides the process of muscle repair by supporting myogenesis and extracellular matrix (ECM) remodeling by fibroadipogenic progenitors (FAPs) (Hogarth et al, 2025; Wosczyna and Rando, 2018). This sequence of events often goes awry in dystrophies due to chronic inflammation and leads to fibrofatty muscle degeneration (Hogarth et al, 2025; Hogarth et al, 2022). To assess these pathogenic features, we immunostained early symptomatic spinal muscles of BLA/J mice for signs of myofiber damage (IgM) and myofiber fatty degeneration (perilipin) (Fig. 5). IgM staining showed that the most prominent myofiber damage occurred in the iliocostalis muscles even at 6 months of age (Fig. 5A,D,E). The number of these IgM^+^ fibers in the iliocostalis increased significantly at 8-10 months of age (Fig. 5B–E). Using perilipin to mark areas with degenerated myofibers in the iliocostalis showed a significant increase starting at 8 months of age, which progressively worsened by 10 months of age (Fig. 5F). Compared to the iliocostalis, the longissimus and multifidus muscles showed relatively limited myofiber damage and adipogenic degeneration by 10 months of age (Fig. 5D,F). Taken together, these histological analyses of BLA/J mice reveal that muscle damage, followed by immune cell infiltration to clear the debris, which becomes chronic and results in the fibrofatty muscle degeneration, is the mechanistic feature of spinal muscles, and addressing these sequelae of dysferlin deficiency on spinal muscle is a potential therapeutic target.

### Spinal FAPs show increased adipogenic potential amenable to therapeutic intervention

**Figure 6:**
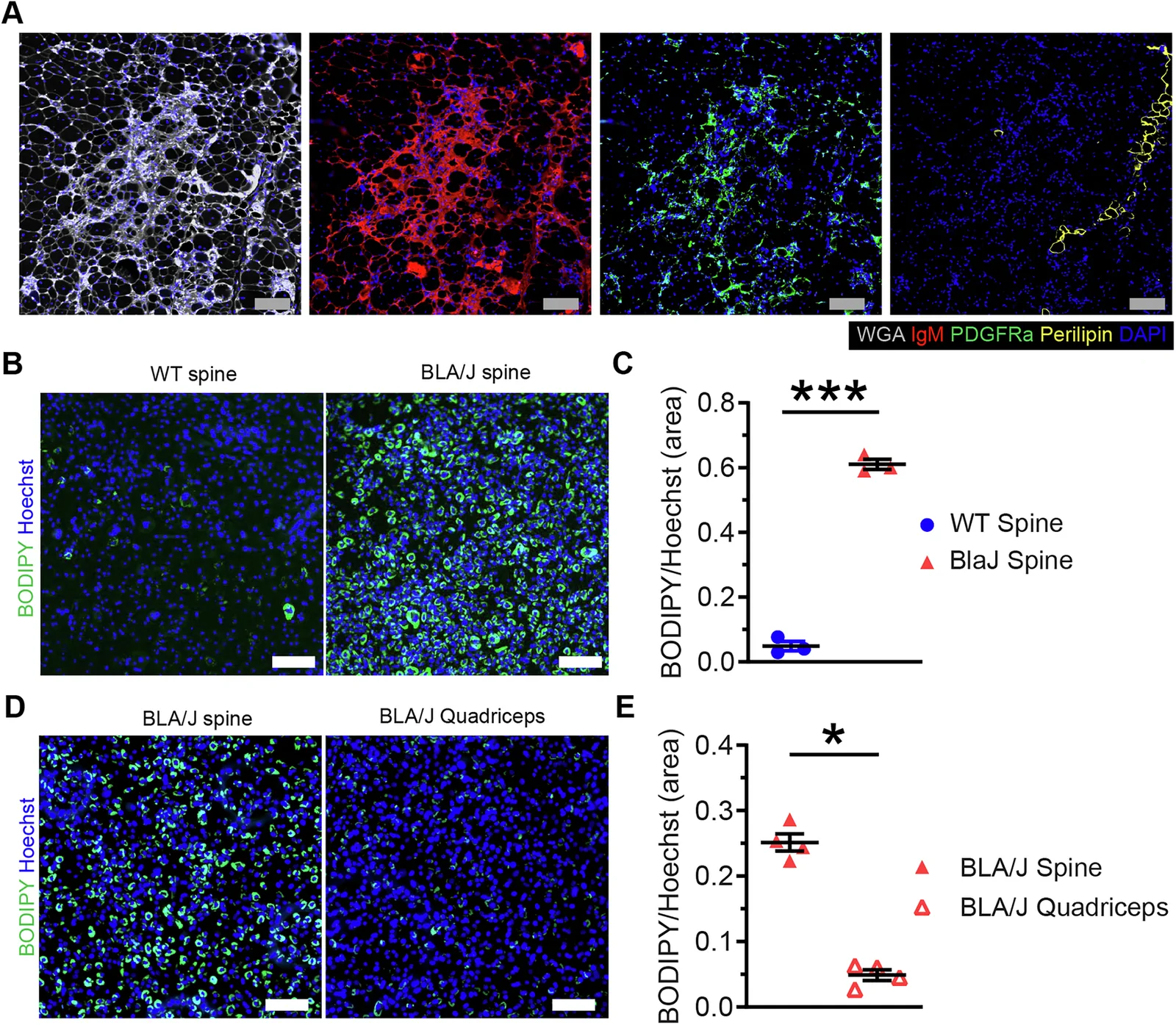
Increased spontaneous adipogenesis of dysferlinopathic FAPs from spinal versus quadriceps muscles. (**A**) Immunofluorescence images of BLA/J iliocostalis muscle at 6 months of age showing WGA, IgM, PDGFRα, perlipin, and DAPI in serial cross-sections. (**B**) Images, and (**C**) quantification of the extent of spontaneous in vitro adipogenesis of FAPs from 10-month-old WT and BLA/J mice. (**D**) Images and (**E**) quantification of the extent of spontaneous in vitro adipogenesis of FAPs from spinal and quadriceps muscles of 8-month-old BLA/J mice. The cells were stained with BODIPY to label lipid droplets and Hoechst to mark nuclei after eight days in culture to assess spontaneous adipogenesis. Data were quantified to represent imaged areas positive for BODIPY staining, normalized to Hoechst-positive area to represent nuclear abundance (*n* = 4). Scale bar = 100 µm. The values represent mean ± SD. ***P < 0.0001 versus WT by Student’s *t* test. Source data are available online for this figure.

As FAPs are central players in fibrofatty muscle degeneration, they are attractive and viable cellular targets to inhibit muscle loss in muscular dystrophy (Hogarth et al, 2025). As PDGFRα expression marks the FAPs, we immunostained the spinal muscles of 6-month-old BLA/J mice for PDGFRα to assess FAP abundance in the spinal muscle. We observed that the areas enriched in IgM-labeled damaged myofibers are also highly enriched in PDGFRα-labeled FAPs (Fig. 6A). Upon differentiation, FAPs and pre-adipocytes lose PDGFRα expression and instead adopt the differentiated cell marker, perilipin, which labels adipocytes (Contreras et al, 2019; Uezumi et al, 2010; Vaziri and Faller, 1996). Consistent with this feature of FAPs, we observed reduced staining for PDGFRα (FAPs) in areas enriched in perilipin labeling (adipocytes) (Fig. 6A). With the difference in the extent of fatty degeneration of the spinal muscles in WT versus BLA/J mice, we next examined the adipogenic potential of FAPs isolated from the iliocostalis muscle of 8-month-old WT and BLA/J mice. Adipogenic differentiation of FAPs was monitored by staining with the neutral lipid probe BODIPY to visualize lipid accumulation. This showed significant adipogenesis of FAPs from the iliocostalis muscles of BLA/J mice as compared to the WT mice (Fig. 6B,C). Next, we examined spontaneous adipogenesis of FAPs isolated from the iliocostalis versus quadriceps muscles of BLA/J mice and observed the iliocostalis-derived FAPs to have significantly greater adipogenic potential (Fig. 6D,E). This identified greater commitment indicated by increased propensity of FAPs from iliocostalis muscles of BLA/J to undergo adipogenesis as compared to the FAPs from WT iliocostalis or from BLA/J quadriceps muscles, indicating targeting FAP adipogenesis as a potential target for therapy.

### Potential therapeutic strategies to inhibit spinal FAP adipogenesis

**Figure 7:**
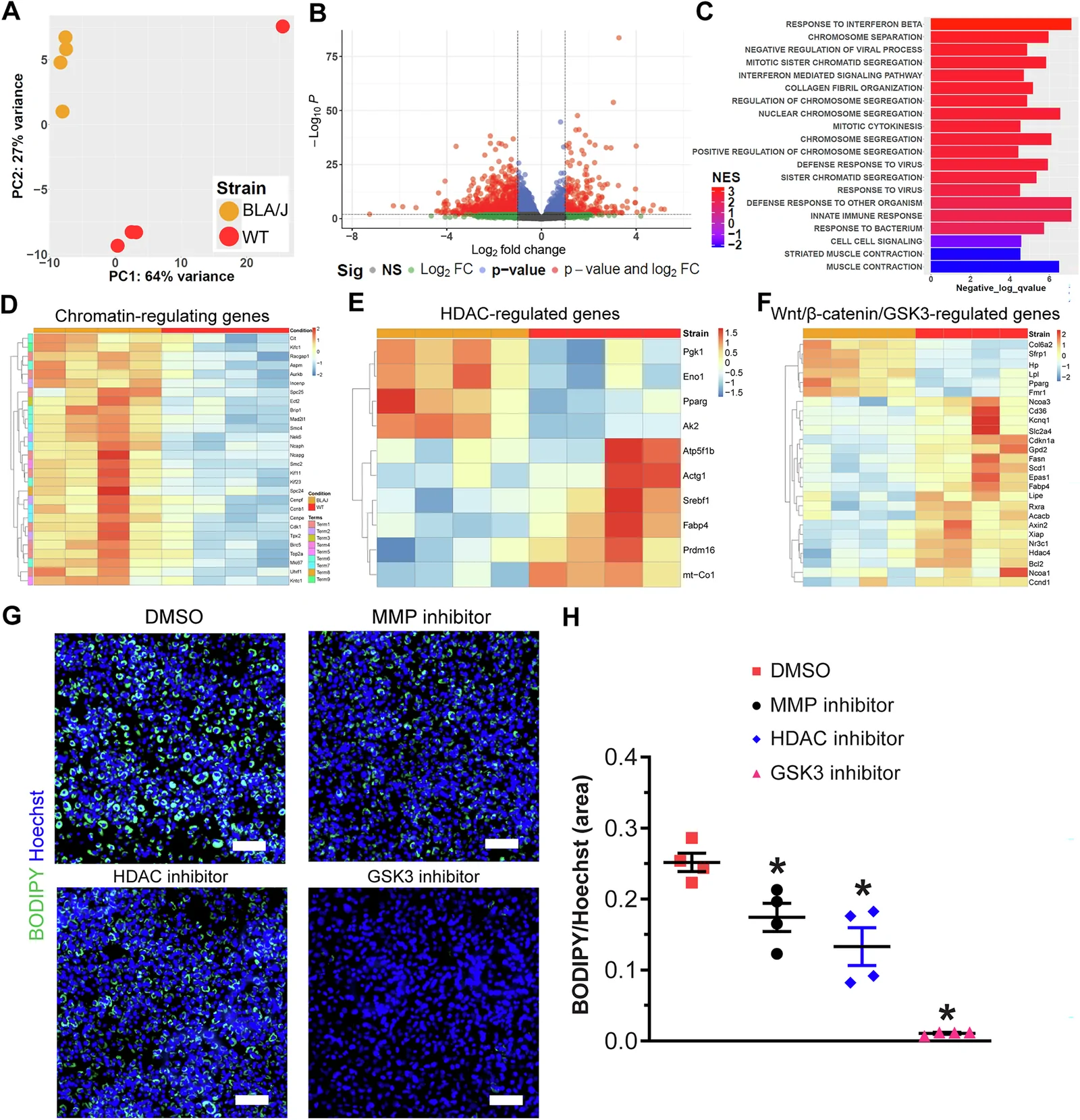
Molecular analysis and therapeutic targeting of BLA/J FAP adipogenesis. (**A**) PCA plot showing the difference between BLA/J (gold) and wild-type (WT, red) mouse FAPs. (**B**) Volcano plot representing the 1,035 differentially expressed genes between FAPs from BLA/J and WT mice with adjusted *P* value < 0.05 and log2FC ≥ 1 determined using Deseq2 with *P* value determined using Wald test, and thresholds are adjusted *P* value < 0.05 and log FC >= |1| evaluated by Benjamini–Hochberg (BH) test. (**C**) Bar plot for the top 20 dysregulated biological processes. *Y* axes represent negative log FDR (qvalue), and color represents the Normalized enrichment score (NES); red – upregulated and blue - downregulated. (**D**–**F**) Heatmap of genes associated with the (**D**) terms involved in chromatin regulation pathways. Term 1: Chromatid segregation, Chromatid separation, Mitotic Sister chromatid segregation, Nuclear Chromatid segregation, Positive regulation of chromosome segregation, regulation of chromosome segregation, and Sister chromatid segregation. Term 2: Chromatid segregation, chromatid separation, mitotic sister chromatid segregation, nuclear Chromatid segregation, regulation of chromosome segregation, and sister chromatid segregation. Term 3: Chromatid segregation, Mitotic Sister chromatid segregation, Nuclear Chromatid segregation, Positive regulation of chromosome segregation, regulation of chromosome segregation, and Sister chromatid segregation. Term 4: Chromatid segregation, Mitotic Sister chromatid segregation, Nuclear Chromatid segregation, regulation of chromosome segregation, and sister chromatid segregation. Term 5: Chromatid segregation, Chromatid separation, Nuclear Chromatid segregation, and sister chromatid segregation. Term 6: Chromatid segregation, Mitotic Sister chromatid segregation, Nuclear Chromatid segregation, and sister chromatid segregation. Term 7: Chromatid segregation and Nuclear Chromatid segregation. Term 8: Chromatid segregation and regulation of chromosome segregation. Term9: Regulation of chromosome segregation, (**E**) HDAC pathway, (**F**) Wnt/β-catenin/GSK3 pathway, (**G**) images, and (**H**) quantification of the extent of spontaneous in vitro adipogenesis by spinal FAPs isolated from 10-month BLA/J mice and treated with DMSO, batimastat (10 µM), Givinostat (50 nM), or LY2090314 (10 nM). BODIPY and Hoechst staining were performed, and quantification represents BODIPY staining normalized to nuclear abundance (*n* = 4 separate coverslips). Scale bar = 100 µm. The values are mean ± SD. *P < 0.05, ***P < 0.001 versus DMSO by Mann–Whitney test. Source data are available online for this figure.

To identify therapeutic targets to inhibit the adipogenic potential of FAPs from dysferlinopathic muscles, we performed RNAseq analysis of freshly isolated FAPs from muscles of 10-month-old WT and BLA/J mice (*n* = 4 each). Principal component analysis (PCA) of this transcriptomic dataset identified distinct clustering of FAPs from the WT and BLA/J mice (Fig. 7A). We found 1035 differentially expressed genes (DEGs - Log_2_ fold change >1; *P* value < 0.05) with 762 genes downregulated and 273 genes upregulated in the BLA/J FAPs (Fig. 7B). Gene ontology (GO) analysis identified BLA/J FAPs exhibit upregulation of genes involved in cell cycle, chromatin organization, and inflammatory responses, while genes that regulate muscle function are downregulated (Fig. 7C,D).

These broad-ranging cellular functions are known to be regulated by Histone deacetylases (HDACs), which function to deacetylate proteins involved in regulating cell cycle and chromatin organization, affecting cell proliferation (Lagger et al, 2002; Telles and Seto, 2012). HDAC activity also alters expression of adipogenic transcription factors (TFs), including PPARγ, C/EBPβ, and SREBP1α (Lagace and Nachtigal, 2004; Lv et al, 2021). It also acetylates TFs like C/EBPα among others, altering their chromatin accessibility and adipogenic metabolism through proteins including *Otub1, Pcna, Eno1*, and *Actg1*, among others (Haberland et al, 2010; Xu et al, 2013). We found that several of these genes were also dysregulated in BLA/J FAPs (Fig. 7E). Due to these features, the ability of HDAC inhibitors to regulate expression of these genes, and recent approval of the HDAC inhibitor Givinostat for Duchenne Muscular Dystrophy treatment (Consalvi et al, 2013), we tested if this could prevent adipogenesis of spinal FAPs from dysferlinopathic mice. Our previous work has identified that MMP inhibitor Batimastat can inhibit dysferlinopathic FAP adipogenesis (Hogarth et al, 2019), therefore we used this drug as a positive control. Givinostat treatment of spinal FAPs from 8-month-old BLA/J mice inhibited their spontaneous adipogenesis to the same extent as Batimastat (Fig. 7G,H).

A larger group of differentially expressed genes between the WT and BLA/J FAPs included those linked to the Wnt/β-catenin pathway. Notably, these genes include the classic transcriptional targets of β-catenin - Cyclin D1 (*Ccnd1*), p21(CIP1/WAF1- *Cdkn1a*), and Axin (*Axin2)* (Jho et al, 2002; Shtutman et al, 1999; van de Wetering et al, 2002) (Fig. 7F). They also included adipogenic co-regulators of Wnt signaling - nuclear co-receptor (*Ncoa-1*), fatty acid synthase (*Fasn*), fatty acid binding protein 4 (*Fabp4)*, stearoyl CoA desaturase 1 *(Scd1)* (Bagchi et al, 2020a; Bagchi et al, 2020b; de Winter and Nusse, 2021; Ross et al, 2000; Triki et al, 2017; Yang Loureiro et al, 2023) (Fig. 7F). As GSK3 is the central regulator that controls the Wnt/β-catenin pathway to modulate adipogenesis (Reggio et al, 2020; Zhang et al, 2024), we tested the effect of a potent GSK3 inhibitor LY2090314. Unlike the partial inhibition of spontaneous BLA/J FAP adipogenesis by HDAC and MMP inhibition, GSK3 inhibition fully abolished spontaneous adipogenesis of dysferlinopathic FAPs (Fig. 7G,H). These results identify GSK3 inhibition as a particularly strong druggable target and a promising therapeutic approach. Together, the molecular analyses and targeted in vitro drug testing open avenues to counter the enhanced adipogenic commitment of BLA/J spinal FAPs, offering potential strategies to nearly abolish the adipogenic degeneration of dysferlinopathic spinal muscle described in this study.

### Canonical Wnt signaling enables BLA/J FAP adipogenesis, and GSK3 inhibition reverses it

**Figure 8:**
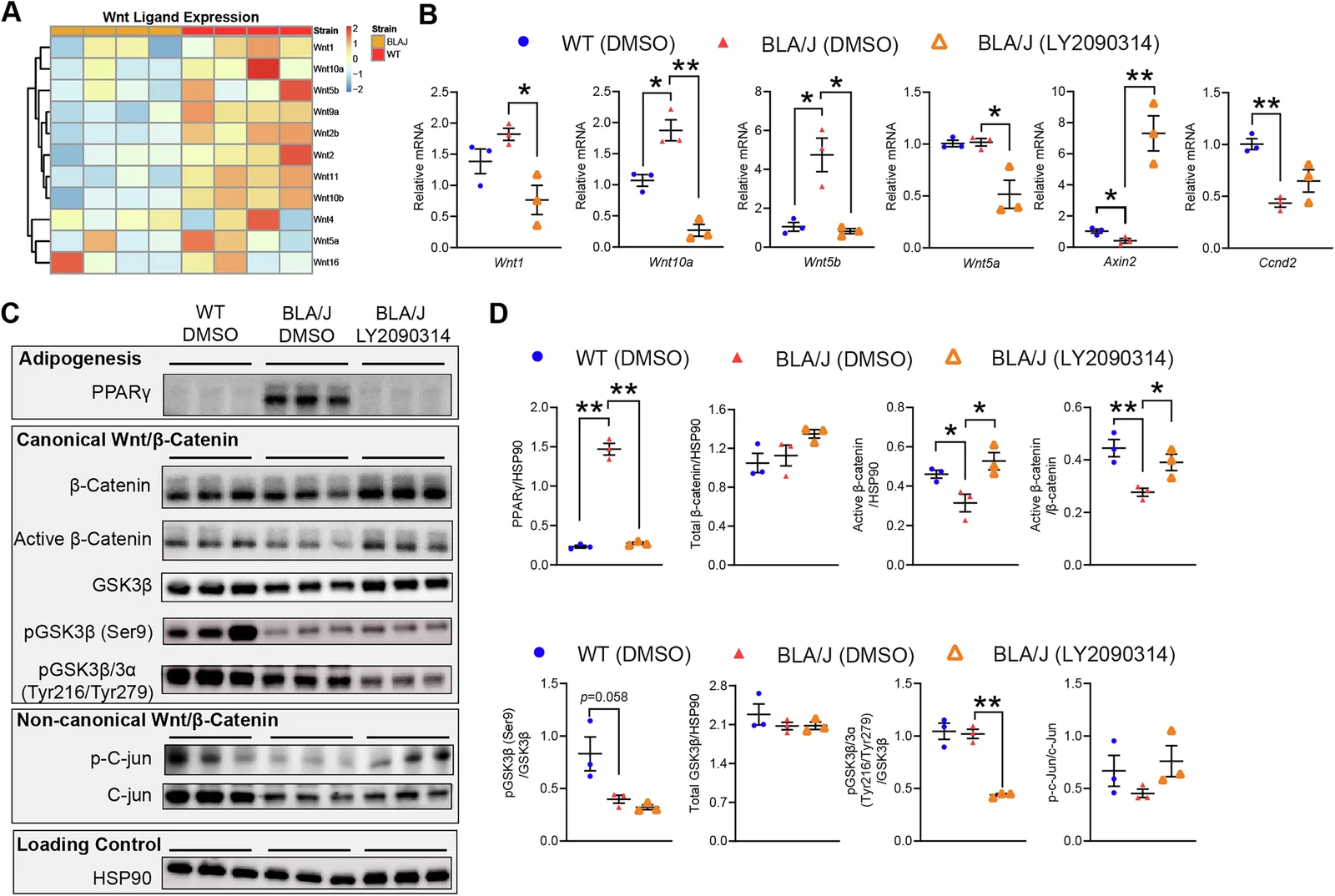
Assessment of altered canonical Wnt/β-catenin signaling in dysferlinopathic FAPs and its reversal by GSK3 inhibition. (**A**) Heatmap showing the differential expression of Wnt ligands in FAPs from WT versus BLA/J muscles (*n* = 4). (**B**–**D**) FAPs from the iliocostalis muscles were treated with DMSO or 20 nM LY2090314 for 3 days and analyzed for β-catenin signaling pathway. (**B**) Plots showing qPCR-based quantification of mRNA for select Wnt ligands in FAPs from WT and BLA/J treated as above with DMSO (control) or with the drug (*n* = 3). (**C**) Images and (**D**) quantification of Western Blots to assess the abundance of the key proteins in the FAP that regulate adipogenesis and Wnt/β-catenin signaling (*n* = 3 separate samples). The values are mean ± SD. *P < 0.05, **P < 0.01 versus DMSO by Mann–Whitney test. Source data are available online for this figure.

Given the strong inhibitory effect of GSK3 inhibition on the adipogenic fate of dysferlinopathic FAPs, we investigated the mechanisms underlying this response. Analysis of Wnt ligands identified by RNA-seq comparing WT and BLA/J FAPs revealed increased expression of several canonical and non-canonical Wnt ligands in BLA/J FAPs (Fig. 8A). To validate these findings and determine the effect of GSK3 inhibition on Wnt ligand mRNA expression, we isolated FAPs from WT and BLA/J mice and treated them with LY2090314 or DMSO (control). qPCR analysis confirmed that the top three Wnt ligands *Wnt1*, *Wnt5b*, and *Wnt10a* identified by RNAseq showed a trend or a significant overexpression in BLA/J FAPs and noncanonical *Wnt5a*, which showed a lower increase by RNAseq, was unchanged in BLA/J FAPs by qPCR (Fig. 8A,B). Treatment of BLA/J FAPs with LY2090314 suppressed the expression of all of the above Wnt ligands (Fig. 8B). To determine whether altered canonical Wnt signaling contributes to the adipogenic phenotype, we assessed TCF/LEF activity by measuring *Axin2* and *Ccnd2*. Both transcripts were reduced in BLA/J compared to WT FAPs, and LY2090314 treatment trended to reverse this inhibition, indicating restoration of canonical Wnt signaling (Fig. 8B).

At the protein level, dysferlinopathic FAPs showed that the high adipogenicity of BLA/J FAPs aligned with an elevated level of PPARγ in BLA/J FAPs that was decreased by treatment with LY2090314 (Fig. 8C,D). Concomitant with this, we observed reduced levels of active β-catenin and a decrease in the ratio of active to total β-catenin with no change in the level of total β-catenin (Fig. 8C,D). This increase in β-catenin activity in BLA/J FAPs was associated with reduced inhibitory Ser9 phosphorylation of GSK3β with no change in the level of total or Tyr216/279 activated GSK3β (Fig. 8C,D). While LY209314 treatment did not affect total or Ser9-inhibited GSK3β, it significantly reduced the level of Tyr216/279-activated GSK3β (Fig. 8C,D). Together, these findings demonstrate that the canonical Wnt/β catenin signaling is impaired in BLA/J FAPs and is restored by GSK3 inhibition. In contrast, analysis of non-canonical signaling, showed highly variable c-Jun phosphorylation in WT FAPs, with no change in the ratio of phosphorylated to total c-Jun between WT and BLA/J FAPs with or without treatment with LY2090314 (Fig. 8C,D).

Together, these findings support a model in which increased adipogenesis of dysferlinopathic BLA/J FAPs is due to elevated Wnt ligand expression with impairment of canonical Wnt/β-catenin signaling due to increased GSK3β activity. This leads to reduced active β-catenin, increased PPARγ expression, and enhanced adipogenesis. Treatment of BLA/J FAPs with LY2090314 reverses the impaired canonical Wnt/β-catenin signaling by reducing the activated pTyr216/279-GSK3β, which restores β-catenin signaling and inhibits PPARγ expression, inhibiting their adipogenic differentiation.

### GSK3 inhibition prevents fatty degeneration of dysferlinopathic muscle

**Figure 9:**
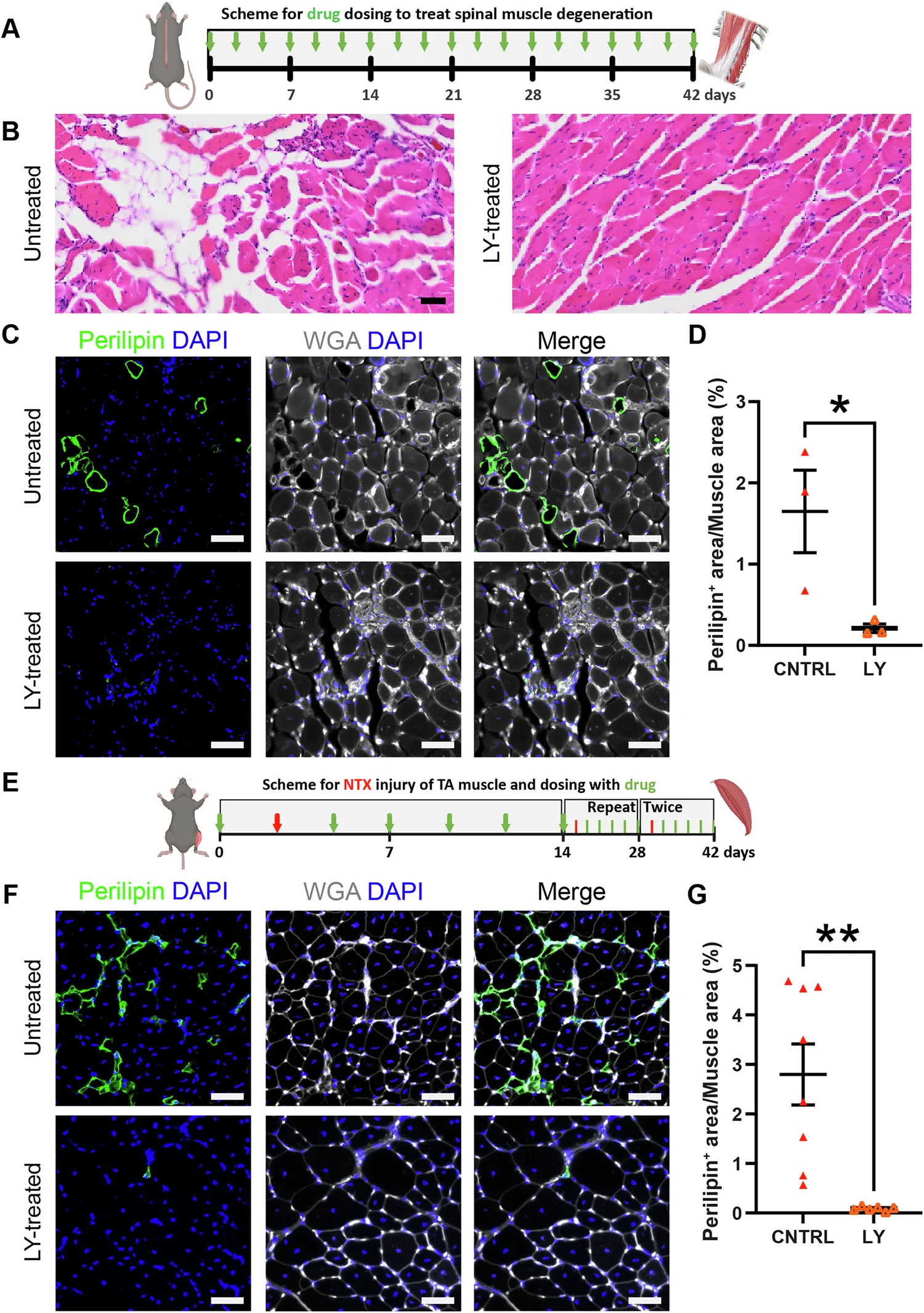
Reduced adipogenic muscle loss in dysferlinopathic mice by GSK3 inhibition of Wnt/ß-catenin signaling. (**A**) Schematic showing the experimental approach used to test the in vivo effect of LY2090314 treatment on spontaneous adipogenic degeneration of the spinal muscles in BLA/J mice. (**B**) Hemotoxylin and eosin staining of spinal muscles of control and LY2090314-treated mice. (**C**) Images and quantification (**D**) showing adipogenesis (marked by perilipin 1) staining of control and LY2090314-treated BLA/J spinal muscle. (**E**) Schematic for the experimental approach used to test the in vivo effect of LY2090314 treatment on adipogenic degeneration of the BLA/J TA muscles upon 3 successive notexin injuries. (**F**) Images and (**G**) quantification of intramuscular adipogenesis in both the TA muscles from control BLA/J mice or those treated with LY2090314. The values are mean ± SD. *P < 0.05, **P < 0.01 versus untreated by two-tailed Student’s *t* test, (*n* = 3–4 mice). Scale bar = 50 µm. Source data are available online for this figure.

**Figure EV3:**
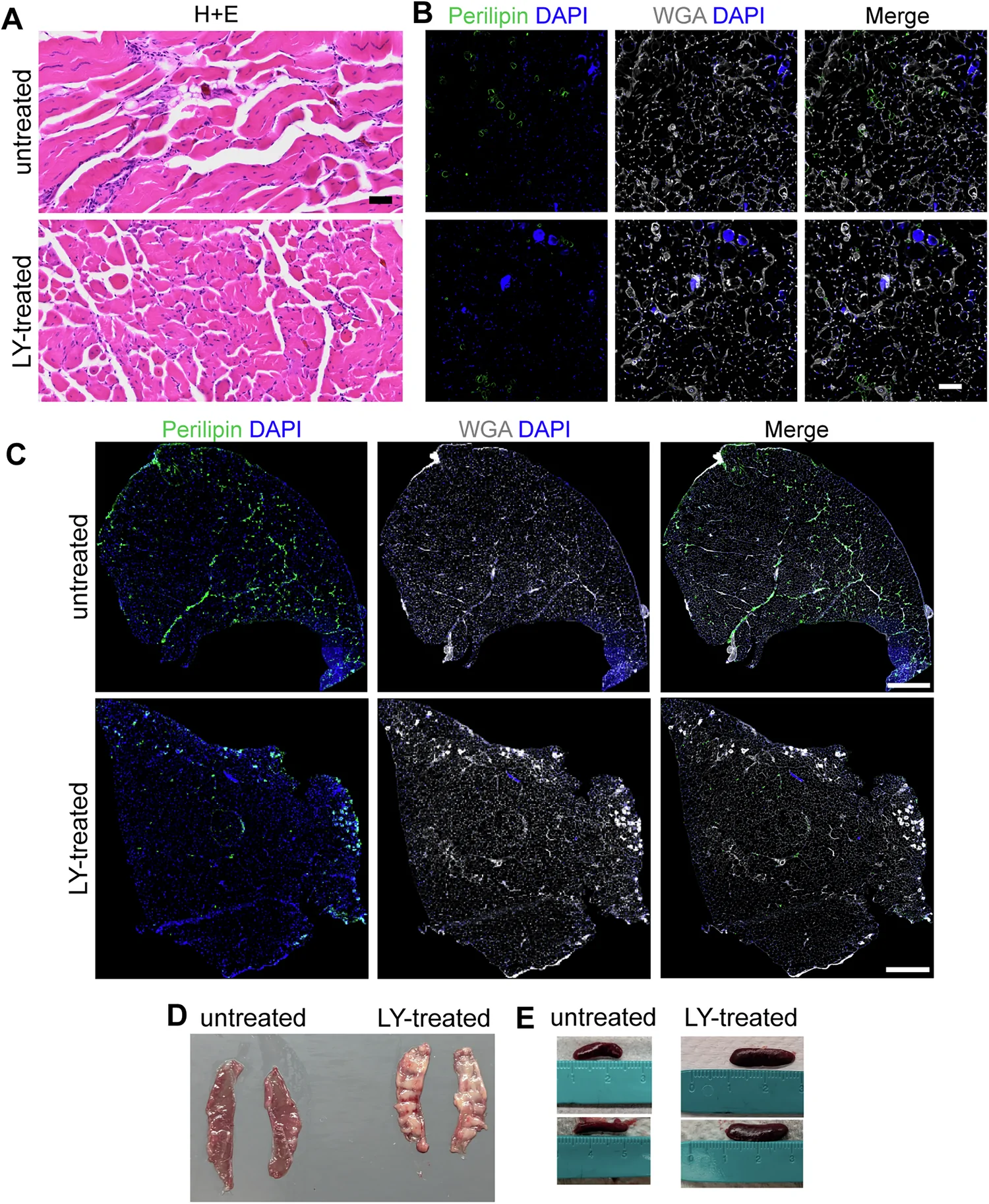
Effects of LY2090314 treatment on spinal, limb, diaphragm muscles, and spleen of dysferlinopathic mice. (**A**) H&E and (**B**) immunofluorescence images of spinal muscles from a pair of untreated and LY2090314-treated BLA/J mice (independent of those in Fig. 9B). Scale bar = 50 µm. (**C**) Immunofluorescence images of the entire cross-section of the TA muscle from untreated and LY2090314-treated BLA/J mice. Scale bar = 500 µm. Images of (**D**) Diaphragm and (**E**) Spleen from a pair of untreated and LY2090314-treated BLA/J mice.

The above results support the potential therapeutic value of GSK3 inhibition to limit adipogenic loss of dysferlinopathic muscle. To test this potential, we carried out in vivo GSK3 inhibition by LY2090314. We treated 7-months old BLA/J mice with LY2090314 on alternate days for 6 weeks to assess if this extended period of treatment will impact the spontaneous onset of adipogenic degeneration of spinal muscles in these mice (Fig. 9A). Examination of the gross pathology of the spinal muscle from untreated and drug-treated BLA/J muscle showed a notable preservation of the spinal muscle in drug treatment mice (Figs. 9B and EV3A). To examine this further, we used perilipin staining to mark the muscle areas replaced by intramuscular adipose tissue, indicative of fatty degeneration of the myofibers. In the placebo-treated control BLA/J mice, we observed abundant perilipin staining in of the spinal muscle, which was lacking in the LY2090314-treated mice (Figs. 9C,D and EV3B).

To determine whether the effect of LY2090314 on adipogenic muscle loss is mediated through inhibition of injury-triggered adipogenic fate specification in BLA/J FAPs, we performed focal injury of the asymptomatic TA muscles in the BLA/J mice using the approach we previously described (Hogarth et al, 2019). This approach involved three consecutive rounds of injury with 2 weeks in between each injury to allow muscles to repair (Fig. 9E). We previously identified that this can be prevented by in vivo treatment with the hedgehog signaling inhibitor Batimastat (Hogarth et al, 2019). In our current study, we treated mice with alternate-day dosing with LY2090314 starting from the day before injury and continuing for the next 6 weeks. Assessment of perilipin immunostaining of the injured TA muscles from control-treated mice confirmed that 3 bouts of successive injury triggered robust intramuscular fat after 3-bouts of injury (Figs. 9F,G and EV3C). In contrast, TA muscles from LY2090314-treated mice were completely protected from injury-induced adipogenesis (Figs. 9F,G and EV3C). Despite the noted benefits of the LYS2090314 treatment in preventing adipogenic muscle loss, the treated mice showed multiple adverse effects, which ranged from scarring of the skin and the diaphragm (site of intraperitoneal injection) and enlargement of the spleen (Fig. EV3D,E). While these effects should be considered in future efforts to develop long-term therapeutic use of this GSK3 inhibitor, our in vivo analysis validates the in vitro studies, identifying the suitability of GSK3 as a target to inhibit adipogenesis of dysferlinopathic muscle resident FAPs and establish its therapeutic potential to prevent adipogenic muscle loss in dysferlinopathy.

## Discussion

Dysferlinopathy is a limb-girdle muscular dystrophy that manifests initially with progressive muscle weakness in the lower extremities starting in the late teens. This disease is characterized by greater adipogenic degeneration of the affected muscles in the posterior compartment of the thigh and calf as compared to the pelvic muscles (Bolano-Diaz et al, 2024; Diaz et al, 2016). However, dysferlinopathic patients also have axial weakness, even camptocormia, that accompany lower limb symptoms (Gati et al, 2012; Nagashima et al, 2004), and chronic lower back pain with minimal lower limb symptoms with MRI diagnosis of fatty degeneration of erector spinae muscles (Kim et al, 2020; Seror et al, 2008). It remains unclear whether the spinal lesions result from altered posture or gait caused by lower limb weakness, or from a primary spinal muscle damage. Use of a comparative analysis of dysferlinopathic patients and mouse model (BLA/J) has helped us tackle this question, as the quadrupedal gait of mice results in greater stability of erector spinae than the bipedal gait of human (Valentin and Licka, 2015). Longitudinal MRI analysis of the erector spinae and quadriceps muscles in BLA/J mice, from the presymptomatic to symptomatic stages of disease, demonstrated that the erector spinae muscles exhibit fatty degeneration prior to the quadriceps muscle. Histological analysis confirmed this at the cellular level as we observed greater adipogenic potential of FAPs from spinal muscles as compared to FAPs from the quadriceps.

Spinal muscle degeneration occurs in several muscular dystrophies but is frequently overlooked (Witting et al, 2016). High-resolution spinal muscle MRI allowed us to compare the severity and specificity of fatty degeneration in spinal muscle in our patient cohort. This showed increased severity among multifidus, longissimus, and iliocostalis, with iliocostalis being the earliest affected and most severely affected, even at a stage when psoas and quadratus lumborum muscles were mildly affected. This feature is noted in *ANO5*-related myopathy (LGMDR12), where fatty degeneration is observed in the multifidus, longissimus, and iliocostalis, and the psoas muscle is less affected, but here the difference was that fatty degeneration appears first in the multifidus and longissimus (Alvarez-Velasco et al, 2022; Sarkozy et al, 2012; Silva et al, 2019). *RYR1*-associated myopathy causes fatty degeneration of paraspinal muscles similar to LGMDR12, but this is asymmetrical along the body axis (Jungbluth et al, 2009; Loseth et al, 2013). Compared to this, calpainopathy (LGMDR1) predominantly causes fatty degeneration of the iliocostalis and longissimus, with sparing of the multifidus, and fatty degeneration of psoas (Barp et al, 2020), In the late-onset Pompe disease, the multifidus muscle is the first to show fatty degeneration, and the psoas is also affected (Alejaldre et al, 2012). In facioscapulohumeral muscular dystrophy (FSHD), fatty degeneration was severe in the multifidus, longissimus, and iliocostalis, with affected quadratus lumborum, but not the psoas (Jordan et al, 2011).

While these MRI-based differences in the pattern of fatty degeneration of the paraspinal muscles can be diagnostic for dysferlinopathic patients, it also raises the question of the early degeneration of the iliocostalis muscle among the paraspinal muscles in dysferlin-deficient patients and mice. One possible cause is anatomical: unlike other paraspinal muscles that originate from the processes of the lumbar vertebrae, the iliocostalis muscle originates from and is enclosed by the thoracolumbar fascia, which contains abundant fibrous connective tissue (Cramer et al, 2014). Additionally, because it is positioned at the outermost region of the erector spinae muscle, it is exposed to the fibrous tissue of the fascia (Schuenke et al, 2012). In BLA/J mice, adipogenic deposit is seen both in the epimysium and the lateral side of the iliocostalis – an area, located at the cephalic of the iliac crest, thought to be an expansion of fibrous tissue from the origin of the iliocostalis (Hesse et al, 2010). Similarly, a so-called fatty tent structure exists between the epimysium and fascial plane of the iliocostalis and longissimus in the normal human lumbar spine area (Berry et al, 2020). Another cause for the greater effect of the iliocostalis is the mechanical property - the iliocostalis fiber bundle has the lowest stiffness compared to the multifidus and longissimus fiber bundle, suggesting the vulnerability of the iliocostalis muscle to damage (Ward et al, 2009).

Dysferlin deficiency compromises injured myofiber repair, and treating this is therapeutic (Bansal et al, 2003; Bittel et al, 2022; Defour et al, 2014; Sreetama et al 2018; Gushchina et al 2017). Thus, increased degeneration of the iliocostalis in dysferlinopathy may be caused by greater damage to this muscle. In support of this, histological analysis of the iliocostalis muscle in the 6-month-old BLA/J mice, which shows minimal adipogenic degeneration by MRI, showed the presence of damaged fibers marked by IgM (indicating sarcolemmal damage) and regenerating fibers. This space also contained abundant inflammatory cells with limited adipogenesis but was abundant in FAPs. Over time, the IgM-labeled fibers get replaced by perilipin-positive adipose tissue. This sequential progression from damaged (and unrepaired) to subsequent fatty replacement of iliocostalis by FAP-mediated adipogenesis supports the mechanical damage model for this muscle’s degeneration in dysferlinopathy. This recapitulates the pathologic changes for the limb muscle loss in dysferlinopathy, which are injury, where poor myofiber repair causes secretion of proteins by the damaged fiber, which support the recruitment of immune cells and FAPs in muscle prior to myofiber necrosis (Fanin and Angelini, 2002; Hogarth et al, 2019; Mariano et al, 2013; Selcen et al, 2001). Intensive exercise by dysferlinopathic patients is linked to adipogenic muscle loss, and longitudinal MRI shows that even routine activity in the dysferlinopathic patients is linked to progressive adipogenic muscle replacement (Bolano-Diaz et al, 2025; Moore et al, 2020). These suggest that chronic mechanical stress and muscle damage can reshape the dysferlinopathic muscle microenvironment into a pro-adipogenic niche, altering the fate choice of FAPs in this niche. As spinal muscles are continuously active for postural support and respiration, they may experience sustained damage–regeneration cycles that exacerbate inflammatory and stromal signaling that promoted adipogenic conversion of resident FAPs.

To assess the impact of the muscle niche on the differentiation commitment of FAPs, we compared spontaneous adipogenesis of PDGFRα^+^ cells cultured from the quadriceps and spinal muscles of the same 8-month-old BLA/J mice. FAPs derived from spinal muscles displayed markedly greater adipogenic differentiation than those isolated from quadriceps muscles. These findings indicate that spinal muscles undergo either an earlier or more pronounced transition toward a pro-adipogenic niche. Such a niche is likely characterized by persistent inflammatory signaling, altered extracellular matrix composition, impaired regenerative capacity, and chronic mechanical loading, all of which may bias FAP fate toward adipogenesis. Consequently, the dysferlinopathic spinal muscle niche appears to intrinsically prime resident FAPs toward adipogenic commitment. This is reflected by the increased in vitro adipogenic differentiation of FAPs from the iliocostalis of BLA/J as compared to the FAPs from BLA/J quadriceps and WT iliocostalis. The fact that FAPs isolated from muscles with different degeneration kinetics behave differently suggests that their microenvironment in vivo potentiates their adipogenic differentiation. These changes, including muscle damage and immune cell accumulation, may increase chromatin accessibility to key transcription factors, such as the PPARγ, that are involved in adipogenesis, and such an epigenetic program influences the difference in adipogenesis of FAPs isolated from muscle. Such a hypothesis is supported by our findings that use of an HDAC inhibitor, a major epigenetic modulator, limited the adipogenic fate of the BLA/J spinal muscle FAPs. Adipogenic degeneration of dysferlinopathic muscle driven by FAPs initiates in early symptomatic stages and not only does it persist over the course of disease, but improved muscle function also correlates with reduced lipid accumulation (Agarwal et al, 2019; Bittel et al, 2022; Grounds et al, 2014; Yasa et al, 2023).

RNAseq analysis of BLA/J and WT FAPs identified the potential of targeting HDAC and GSK-3 activity for future therapeutic development. Previously, we found that the use of an MMP-14 inhibitor to regulate hedgehog signaling is an approach to inhibit adipogenesis of dysferlinopathic muscle FAPs and prevent adipogenic deposits in the injured dysferlinopathic muscle (Hogarth et al, 2019). While no approved therapies currently exist to block adipogenic differentiation of FAPs, the continuous nature of this pathological process suggests a broad therapeutic window for such therapy to limit muscle loss, and slow disease progression. Our in vivo studies show that the use of a GSK3 inhibitor can prevent this FAP fate, offering a path towards the development of such therapies. Our finding that the use of GSK-3 inhibitor LY2090314 prevents adipogenesis of dysferlinopathic patients adds to the arsenal of drugs to target dysferlinopathy. Further, prior studies in 3D FAPs culture model and DMD showing that this drug can also prevent FAP adipogenesis, validates and broadens the impact of our finding (Reggio et al, 2023; Reggio et al, 2020). The issues related to the potential safety of Batimastat and LY2090314, limits the clinical utility of these drugs for chronic conditions like dysferlinopathy (Arciniegas Ruiz and Eldar-Finkelman, 2021; Rothenberg et al, 1999). For targeting GSK3, alternate inhibitors such as Tideglusib and CHIR99021 show promise in treating chronic muscle disease. While preclinical use of CHIR99021 prevents muscle loss (Fu et al, 2026; Zhang et al, 2025), chronic Tideglusib use shows therapeutic potential for DMD and myotonic dystrophy type 1 (DM1), reaching phase III clinical testing for myotonic dystrophy (Horrigan et al, 2020; Marcella et al, 2024; Wang et al, 2019). An alternate therapeutic approach includes altering the expression of genes regulated by the HDAC activity, as use of Givinostat inhibited adipogenesis of BLA/J spinal FAPs as effectively as Batimastat. The pan-HDAC inhibitor, Givinostat, has been recently approved for its clinical use in patients with DMD (Mozzetta et al, 2024). Use of this drug improves muscle function and suppresses muscle atrophy, fibrosis, and fatty degeneration in both *mdx* mice models and DMD patients (Bettica et al, 2016; Consalvi et al, 2013; Mercuri et al, 2024; Minetti et al, 2006). However, additional studies to evaluate the potential benefits of this drug in dysferlinopathy is needed. Additional studies are also needed to identify pathways downstream of HDAC that affect the adipogenic fate of dysferlinopathic FAPs, and identify the chromatin and gene expression changes that could further enhance the therapeutic efficacy by targeting the specific pathway downstream of HDAC inhibition that restricts adipogenesis of the dysferlinopathic FAPs. Given the lack of current therapies available to patients with dysferlinopathy, a potential off-label use of DUVYZAT® (Givinostat) could still offer an avenue to prevent spontaneous FAP adipogenesis and spinal muscle degeneration in dysferlinopathy.

In summary, our studies here guided by the initial observation of spinal difficulties experienced by dysferlinopathic patients have led to the confirmation of rapid adipogenic degeneration of erector spine muscles in the early stage of the disease as a hallmark of dysferlinopathy in patients and mouse models. It has uncovered the mechanistic basis for this pathology and identified treatments that could potentially treat this pathology to prevent irreversible muscle fiber loss, which causes disease symptoms in these patients.

## Methods

**Table:** Reagents and tools table

Reagent/resource	Reference or source	Identifier or catalog number
**Experimental models**		
C57BL/6J *(M. musculus)*	Jackson Laboratory	N/A
B6.A-*Dysf*^*prmd*^/GeneJ *(M. musculus)*	Jackson Laboratory	N/A
**Antibodies**		
PDGFRα	Cell Signaling Technology	3174S
Perilipin	Sigma-Aldrich	P1873
Laminin-2α-chain	Sigma-Aldrich	4H8-2
IgM	Invitrogen	A-21043
wheat germ agglutinin	ThermoFisher	W11261
DAPI	ThermoFisher	P36941
APC-anti-CD140a	Biolegend	135908
PPARγ	Santa Cruz Biotechnology	sc-7273
β-catenin	Santa Cruz Biotechnology	sc-7963
Activie β-catenin	Cell Signaling Technology	8814
GSK3β	Cell Signaling Technology	9315
pGSK3β (Ser9)	Cell Signaling Technology	9336
pGSK3β/3α (Tyr216/Tyr279)	Abcam	ab68476
p-c-Jun	Cell Signaling Technology	2361
c-Jun	Cell Signaling Technology	9165
HSP90	Enzo Life Sciences	ADI-SPA-836-F
**Oligonucleotides and other sequence-based reagents**		
*Wnt1*-F	Macrogen	ATGAACCTTCACAACAACGAG
*Wnt1*-R	Macrogen	GGTTGCTGCCTCGGTTG
*Wnt5a*-F	Macrogen	CTCCTTCGCCCAGGTTGTTATAG
*Wnt5a*-R	Macrogen	TGTCTTCGCACCTTCTCCAATG
*Wnt5b*-F	Macrogen	ATGCCCGAGAGCGTGAGAAG
*Wnt5b*-R	Macrogen	ACATTTGCAGGCGACATCAGC
*Wnt10a*-F	Macrogen	CCTGTTCTTCCTACTGCTGCTGG
*Wnt10a*-R	Macrogen	CGATCTGGATGCCCTGGATAGC
*Axin2*-F	Macrogen	ATGGAGTCCCTCCTTACCGCAT
*Axin2*-R	Macrogen	GTTCCACAGGCGTCATCTCCTT
*Ccnd2*-F	Macrogen	GCAGAAGGACATCCAACCGTAC
*Ccnd2*-R	Macrogen	ACTCCAGCCAAGAAACGGTCCA
*Actb*-F	Macrogen	CATTGCTGACAGGATGCAGAAGG
*Actb*-R	Macrogen	TGCTGGAAGGTGGACAGTGAGG
**Chemicals, enzymes, and other reagents**		
Calci-Clear Rapid	National Diagnosis	HS-105
paraformaldehyde	ThermoFisher	AAJ19943K2
bFGF	Biolegend	579604
DMSO	Sigma-Aldrich	D4540
Batimastat	Sigma-Aldrich	SML0041
Givinostat	Selleckchem	ITF2357
LY2090314	Selleckchem	S7063
LY2090314	MedChemExpress	HY-16294
boron difluoride dipyrromethene (BODIPY)	ThermoFisher	D3922
Hoechst	ThermoFisher	33342
protein extraction reagent	ThermoFisher	78505
TRIzol reagent	Invitrogen	15596026
High-Capacity cDNA Reverse Transcription Kit	Applied Biosystems	4374966
notexin	Latoxan	L8104
Direct-zol RNA Miniprep	Xymo Research	R2051
**Software**		
GraphPad Prism 9.0.0	GraphPad	N/A
ImageJ software	NIH Image	N/A
QuPath 0.6.0	QuPath	N/A
CellSens software	Olympus	N/A
**Other**		
Bruker Avance 400	Bruker Biospin	N/A
VS120 slide-scanning microscope	Olympus	N/A
VS200 Virtual Slide Scanning System	Olympus	N/A
DigiGait imaging system	Mouse Specifics	N/A
ultrafine insulin syringe	BD Biosciences	324906
Leica DMi8 microscope	Leica	N/A

### Human subjects

A retrospective analysis was conducted on twenty patients diagnosed with dysferlinopathy at Jeonbuk National University Hospital, Pusan National University Yangsan Hospital, and Hôpital Pitié-Salpêtrière. Patients were included based on the following criteria: progressive proximal muscle weakness, elevated serum creatine kinase (CK) levels, degenerative muscle changes observed on CT or MRI, and genetic confirmation of either two pathogenic heterozygous mutations or one or two homozygous mutations in the *DYSF* gene and absence of dysfelin on muscle biopsy or monocytes. All patients were diagnosed between 2009 and 2022 at Pusan, Jeonbuk National University Hospital and between 1997 and 2023 at Hôpital Pitié-Salpêtrière. The patient cohort was ethnically diverse, comprising 6 Asian patients, 9 White/Caucasian patients, and 5 Arabic patients. All enrolled patients underwent muscle MRI, including imaging of spinal musculature, and serum CK levels were assessed at disease onset. Specifically, five patients underwent lumbar spine MRI, and 15 underwent whole-body MRI [all 14 French patients underwent whole-body MRI]. MRI scans were performed using 3-T clinical MRI systems (PrismaFit, or Magnetom Verio, Siemens Healthcare, Erlangen, Germany), and individuals were imaged by T1-weighted imaging. The Mercuri score was used to grade fatty involvement of muscles (Mercuri et al, 2007). The control group consisted of age- and gender-matched individuals who underwent spine MRI for minor trauma or back pain and had no history of neuromuscular disease. The study protocol was approved by the Institutional Review Boards of Jeonbuk National University Hospital (IRB No. 2022-11-051) and Pusan National University Yangsan Hospital (IRB No. 05-2023-043). The French patients were enrolled in the Clinical Outcome Study for Dysferlinopathy (Jain COS) - ClinicalTrials.gov # NCT01676077. The study was approved by the local ethics committee (Comité de Protection des Personnes [CPP] Île de France VI, ID RCB 2012-A01689-34), and it was registered in accordance with French data protection regulations (CNIL, Commission nationale de l’informatique et des libertés). Informed consent was obtained from all human subjects, and the MRI protocols conformed to the principles set out in the WMA Declaration of Helsinki.

### Mouse model

All animal experiments used in this study were approved by the Children’s National Medical Center Institutional Animal Care and Use Committee (Protocol #30709) and were conducted in accordance with guidelines for the care and use of laboratory animals. WT (C57BL/6 J) and BLA/J (B6.A-*Dysf*^*prmd*^/GeneJ) mice were purchased from Jackson Laboratory (Bar Harbor, ME) and maintained in-house. All animals were maintained in a germ-free facility and individually vented cage system under a controlled 12-h light/ dark cycle with free access to food and water. Anatomical T1 MRI images for mice were acquired on a Bruker Avance400 (Bruker Biospin, Billerica, MA, USA) 9.4 T 89 mm vertical bore MRI with a 26 mm ID volume coil. Two sets of axial T1-weighted fast spin echo scans were acquired, one aligned with the spine and the other aligned with the femur, each scanned twice, with and without fat saturation. Scans were acquired using the RARE (Rapid Acquisition with Refocusing Echoes) sequence: TE = 8.5 ms, TR = 4000 ms, RARE Factor = 4, NA = 12, FOV = 25.6 × 25.6 mm, MTX = 256 × 256, 85 × 0.3 mm slices. To quantify the fat fractions, fat-only images were created by subtracting the T1-weighted with fat-saturation image from the T1-weighted without fat-saturation image using ImageJ image analysis software (version 1.53t, National Institutes of Health, Bethesda, Maryland). We applied a region-of-interest (ROI) approach to test specific structures throughout the lumbar spine and quadriceps in axial view and selected. The ROIs of the spinal muscle and quadriceps muscle were established based on the deep fascia surrounding the muscle observed in T1-weighted MR. The fat fraction was measured within the ROI defined as muscle, and the fat percentage within the spinal muscle or quadriceps was expressed as a percentage by measuring the fat fraction area, respectively, when each muscle ROI was set to 100%. All ROIs were drawn and quantified manually by a blinded experimenter.

### Histology and immunofluorescence

Lumbar spine and quadriceps muscles of mice were collected. The resected lumbar spine with spinal muscles was fixed in 4% paraformaldehyde and decalcified in Calci-Clear Rapid (National Diagnosis, GA, USA) for three days. The spine tissue was cut into 10-µm-thick sections based on the 4th lumbar vertebra. For quadriceps, frozen sections 8-µm thick were cut based on the mid area. To evaluate the histological findings, thawed tissue sections were processed for H&E and immunofluorescence. Immunofluorescence was performed by blocking with 5% BSA and incubation with primary antibodies against PDGFRα (1:250, Cell Signaling, #3174S), perilipin (1:250, Sigma, #P1873), laminin-2α-chain (1:100, Sigma, #clone 4H8-2), and anti-IgM (1:100, Invitrogen, #A-21043). Alexa Fluor 488 and/or 568 conjugated (1:500, ThermoFisher) secondary antibodies were used for visualization and co-stained with wheat germ agglutinin (1:500, ThermoFisher, #W11261) and DAPI (ThermoFisher, #P36941). Images were captured using a VS120 slide-scanning microscope (Olympus America) at 200x magnification and quantified using CellSens software (Olympus America) and ImageJ software (National Institutes of Health).

### Microscopy and image analysis

Frozen sections, 8-µm thick, were cut from the collected mouse muscles. Immunofluorescence was performed by fixing sections in chilled 4% Paraformaldehyde, blocking with 5% BSA in PBST, and incubation with a primary antibody against perilipin-1 (1:250, Sigma, P1873). Myofiber membranes were marked using AlexaFluor 488-conjugated wheat germ agglutinin (1:500, ThermoFisher, #W11261), which was added during incubation with secondary antibody. Coverslips were mounted using ProLong Gold with DAPI (ThermoFisher, #P36941). Microscopy was performed using an Olympus VS200 Virtual Slide Scanning System. Quantification was performed using QuPath 0.6.0 (Bankhead et al, 2017). The quantification was done using the pixel classifier function to get the total area of the muscle section and the area of the perilipin-1 staining, which was used to calculate the total positive area relative to the muscle section area. Analysis of the spinal muscles was done by averaging values across three randomly picked ROIs (800 µm × 800 µm) in the spinal muscle of each mouse and measuring the perilipin-1-stained area over the total area.

### Motor function analysis

Gait dynamic and locomotion analysis were performed using the DigiGait imaging system (Mouse Specifics, Quincy, MA). WT and BLA/J mice were placed on a motorized transparent treadmill within a plexiglass compartment, moving at 18 cm/s (horizontal degree) or 15 cm/s (15° incline and 15° decline). Digital videos of the ventral surfaces of the fore and hind paw placement were acquired using a camera mounted beneath the treadmill. The videos were analyzed using DigiGait software, which automatically processes from nine to twelve strides to calculate gait parameters. Approximately 4 s videos were collected from each mouse.

### Acute muscle injury

Notexin injuries were performed by applying Nair to the anterior hind limbs before intramuscular injection of 50 µL notexin (5 μg/ml, Latoxan, #L8104) into the tibialis anterior using a 0.3-ml ultrafine insulin syringe (BD Biosciences, #324906). Mice were allowed to recover for 14 days before the next injury was performed. Injury was repeated a total of three times, and was collected two weeks after the final injury. For drug treatment, mice at 30 weeks of age were given an intraperitoneal dose of LY2090314 (25 mg/kg; MedChemExpress-HY-16294). The drug was dissolved in DMSO and then diluted in corn oil, 5% and 95%, respectively, for IP injection. Mice were dosed 3 days a week for 6 weeks, starting the day before the first Notexin injury.

### Cell culture studies

FAPs were isolated from the iliocostalis or quadriceps of 8-month-old WT and BLA/J mice. Muscle digestive procedure was previously described (Hogarth et al, 2019). Primary cells isolated from muscles were stained with anti-CD140a-APC (Biolegend, #135908) and isotype control (Biolegend, #400512) for FACS. Stained cells with anti-CD140a-APC were sorted on an Influx cell sorter (Becton Dickinson, #646500). The CD140a^+^ cells were plated in 96-well plates at 15,000 cells/well density. Cells were cultured in FAPs growth media (DMEM with 20% FBS, 1% penicillin, and bFGF (2.5 ng/ml, Biolegend, #579604)) for 3 days to reach 80% confluence and then were switched to adipogenic maintenance media (DMEM with 10% FBS and insulin (10 µg/ml)) for 3 or 5 days. For drug treatment, the CD140a^+^ cells were plated in 96-well plates at 15,000 cells/well density. After reaching 80% confluency, they were switched into the adipogenic maintenance media and then were treated with either DMSO (Sigma-Aldrich), Batimastat (10 µM, Sigma-Aldrich, #SML0041) (Hogarth et al, 2019), Givinostat (50 nM, Selleckchem, #ITF2357) (Milan et al, 2018), or LY2090314 (10 nM, Selleckchem, #S7063) (Reggio et al, 2020) for 3 days. Cells were then fixed in 4% paraformaldehyde for 30 min and stained with boron difluoride dipyrromethene (BODIPY, ThermoFisher, #D3922) and Hoechst (ThermoFisher, #33342) for 30 min. Images were captured with a Leica DMi8 microscope (Leica, Germany) and quantified using ImageJ software (Version 1.54p).

### RNA isolation, qPCR, and western blotting

To evaluate the changes in Wnt signaling and the efficacy of LY2090314 during FAP adipogenesis from WT and BLA/J iliocostalis, we isolated CD140a^+^ from 8-month-old WT or BLA/J mice’s iliocostalis using a cell sorter. The CD140a^+^ cells were plated in 12-well plates at 2 × 10^5^ cells/well density. Cells were cultured in FAPs growth media for 3 days to reach 80% confluence and then were switched to adipogenic maintenance for 3 days with DMSO or LY2090314 (20 nM).

To obtain total RNA, cells were subjected to TRIzol reagent (Invitrogen) extraction. Purified RNA (1 μg) was reverse-transcribed using a first-strand cDNA synthesis kit (Applied Biosystems, Foster City, CA, USA). Quantitative PCR was performed in 384-well plates using the ABI Prism 7900HT Sequence Detection System (Applied Biosystems). Gene expression for all mRNA targets was normalized to *Actb* mRNA transcript levels. RNA from each sample was analyzed individually. The primer sequences used for PCR are listed in Table S1.

We extracted protein from cells using a protein extraction reagent (Thermo Fisher Scientific, 78505). Homogenates (20 μg of total protein) were then separated by 10–15% SDS-PAGE and transferred to nitrocellulose membranes. After blocking the samples with 5% skim milk, the blots were probed with primary antibodies for overnight at 4 °C. They were then incubated with HRP-conjugated secondary antibodies for 1 h at room temperature. Immunoreactive bands were detected using a Las-4000 imager (GE Healthcare Life Science, Pittsburgh, PA, USA). The primary antibodies used for Western blotting were PPARγ (1:2500, Santa Cruz Biotechnology, #sc-7273), β-catenin (1:2500, Santa Cruz Biotechnology, #sc-7963), activie β-catenin (1:2500, Cell Signaling, #8814), GSK3β (1:2500, Cell Signaling, #9315), pGSK3β (Ser9) (1:2500, Cell Signaling, #9336), pGSK3β/3α (Tyr216/Tyr279) (1:2500, Abcam, #ab68476), p-c-Jun (1:1000, Cell Signaling, #2361), c-Jun (1:2500, Cell Signaling, #9165), and HSP90 (1:2500, Enzo Life Sciences, #ADI-SPA-836-F).

### Statistical analysis

GraphPad Prism 9.0.0 was used for statistical analyses of data. Sample sizes were based on prior studies. As detailed in Appendix Table S1, we used simple linear regression to assess relationships between continuous variables, unpaired t-tests and Mann–Whitney tests to compare two independent groups, and two-way ANOVA to evaluate the effects of two factors and their interaction. Holm–Šídák multiple-comparisons tests were used following ANOVA to identify specific group differences while controlling for multiple testing. A Wald test with Benjamini–Hochberg correction was also used to assess statistical significance while controlling the false discovery rate. A *P* value < 0.05 was considered significant. Data plots were reported as scatter plots with mean ± SD. Animals used in the experiments described in the manuscript were chosen for their age and sex as noted, but were randomized for litter and for weight and other physical parameters. The experimenters were not blinded to the genotype, but during data analysis, efforts were made to minimize unblinding of the experimenters until after the analysis.

### RNA-seq analyses

FAPs were isolated from muscle as described above and previously (Hogarth et al, 2019). Cells were counted using a hemocytometer and lysed in TriZol (ThermoFisher Scientific), and total RNA was isolated using a Direct-zol RNA Miniprep (Xymo Research #R2051). For RNA sequencing, libraries were prepared using poly(A) selection and sequenced on an Illumina NovaSeq X platform with 150-bp paired-end reads, generating approximately 30 million reads per sample. Quality control was performed on FASTQ files generated from sequencing using FastQC (version 0.11.9) for individual samples and multiqc (version 1.29) (Ewels et al, 2016) for all samples. After quality trimming with Trim Galore (version 0.6.10), the reads were aligned to the human reference (hg38) using STAR (version 2.7.11b) (Dobin et al, 2013), and the counts were estimated with RSEM (version 1.3.1) (Li and Dewey, 2011). For differential expression, we first filtered out low read counts (<10 reads) and then estimated sample quality using principal component analyses (PCA). Normalization and differential expression were performed with DESeq2 (version 1.46.0) (Love et al, 2014) using an adjusted *p*-value threshold of <0.05 and an average log2 fold change of >1. Gene set enrichment analysis was done with clusterProfiler (version 4.14.6) (Yu et al, 2012) package, utilizing gene sets from the MSigDB (version 2024.1) (Liberzon et al, 2015) database. Visualization of differentially expressed genes using volcano plots was performed using EnhancedVolcano (version 1.24.0) (https://github.com/kevinblighe/EnhancedVolcano), whereas genes associated with GSK3, HDAC, and chromatid-associated pathways were visualized using pheatmap (version 1.24.0).

## Supplementary information

Table EV1

Appendix

Peer Review File

Source data Fig. 1

Source data Fig. 2

Source data Fig. 3

Source data Fig. 4

Source data Fig. 5

Source data Fig. 6

Source data Fig. 7

Source data Fig. 8

Source data Fig. 9

Expanded View Figures

## Data Availability

The RNA-seq data generated as part of this study have been deposited in the. The dataset produced for the Expression profiling by high-throughput sequencing conducted in this study is available in the Gene Expression Omnibus database, with accession number GSE333908 and the secure token (*uropwmqszzgxlmj*) while the record is in private status. The source data of this paper are collected in the following database record: biostudies:S-SCDT-10_1038-S44321-026-00514-5.

## References

[CR1] Agarwal AK, Tunison K, Mitsche MA, McDonald JG, Garg A (2019) Insights into lipid accumulation in skeletal muscle in dysferlin-deficient mice. J Lipid Res 60:2057–2073 10.1194/jlr.RA119000399 PMC6889719 31653658

[CR2] Alejaldre A, Diaz-Manera J, Ravaglia S, Tibaldi EC, D’Amore F, Moris G, Muelas N, Vilchez JJ, Garcia-Medina A, Uson M et al (2012) Trunk muscle involvement in late-onset Pompe disease: study of thirty patients. Neuromuscul Disord 22(Suppl 2):S148–S154 10.1016/j.nmd.2012.05.011 22980766

[CR3] Alvarez-Velasco R, Nunez-Peralta CA, Alonso-Perez J, Gallardo E, Collet-Vidiella R, Reyes-Leiva D, Pascual-Goni E, Martin-Aguilar L, Caballero-Avila M, Carbayo-Viejo A et al (2022) High prevalence of paraspinal muscle involvement in adults with McArdle disease. Muscle Nerve 65:568–573 10.1002/mus.27523 35174518

[CR4] Angelini C (2020) LGMD. Identification, description and classification. Acta Myol 39:207–217 10.36185/2532-1900-024 PMC7783424 33458576

[CR5] Arciniegas Ruiz SM, Eldar-Finkelman H (2021) Glycogen synthase kinase-3 inhibitors: preclinical and clinical focus on CNS-A decade onward. Front Mol Neurosci 14:792364 10.3389/fnmol.2021.792364 PMC8813766 35126052

[CR6] Bagchi DP, Li Z, Corsa CA, Hardij J, Mori H, Learman BS, Lewis KT, Schill RL, Romanelli SM, MacDougald OA (2020a) Wntless regulates lipogenic gene expression in adipocytes and protects against diet-induced metabolic dysfunction. Mol Metab 39:100992 10.1016/j.molmet.2020.100992 PMC7264081 32325263

[CR7] Bagchi DP, Nishii A, Li Z, DelProposto JB, Corsa CA, Mori H, Hardij J, Learman BS, Lumeng CN, MacDougald OA (2020b) Wnt/beta-catenin signaling regulates adipose tissue lipogenesis and adipocyte-specific loss is rigorously defended by neighboring stromal-vascular cells. Mol Metab 42:101078 10.1016/j.molmet.2020.101078 PMC7554252 32919095

[CR8] Bankhead P, Loughrey MB, Fernandez JA, Dombrowski Y, McArt DG, Dunne PD, McQuaid S, Gray RT, Murray LJ, Coleman HG et al (2017) QuPath: open source software for digital pathology image analysis. Sci Rep 7:16878 10.1038/s41598-017-17204-5 PMC5715110 29203879

[CR9] Bansal D, Miyake K, Vogel SS, Groh S, Chen CC, Williamson R, McNeil PL, Campbell KP (2003) Defective membrane repair in dysferlin-deficient muscular dystrophy. Nature 423:168–172 10.1038/nature01573 12736685

[CR10] Barp A, Laforet P, Bello L, Tasca G, Vissing J, Monforte M, Ricci E, Choumert A, Stojkovic T, Malfatti E et al (2020) European muscle MRI study in limb girdle muscular dystrophy type R1/2A (LGMDR1/LGMD2A). J Neurol 267:45–56 10.1007/s00415-019-09539-y 31555977

[CR11] Bashir R, Britton S, Strachan T, Keers S, Vafiadaki E, Lako M, Richard I, Marchand S, Bourg N, Argov Z et al (1998) A gene related to Caenorhabditis elegans spermatogenesis factor fer-1 is mutated in limb-girdle muscular dystrophy type 2B. Nat Genet 20:37–42 10.1038/1689 9731527

[CR12] Berry DB, Rodriguez-Soto AE, Englund EK, Shahidi B, Parra C, Frank LR, Kelly KR, Ward SR (2020) Multiparametric MRI characterization of level dependent differences in lumbar muscle size, quality, and microstructure. JOR Spine 3:e1079 10.1002/jsp2.1079 PMC7323468 32613159

[CR13] Bettica P, Petrini S, D’Oria V, D’Amico A, Catteruccia M, Pane M, Sivo S, Magri F, Brajkovic S, Messina S et al (2016) Histological effects of givinostat in boys with Duchenne muscular dystrophy. Neuromuscul Disord 26:643–649 10.1016/j.nmd.2016.07.002 27566866

[CR14] Bittel DC, Sreetama SC, Chandra G, Ziegler R, Nagaraju K, Van der Meulen JH, Jaiswal JK (2022) Secreted acid sphingomyelinase as a potential gene therapy for limb girdle muscular dystrophy 2B. J Clin Invest 132:e141295 10.1172/JCI141295 PMC8718136 34981776

[CR15] Bolano-Diaz C, Verdu-Diaz J, Gonzalez-Chamorro A, Fitzsimmons S, Veeranki G, Straub V, Diaz-Manera J (2024) Magnetic resonance imaging-based criteria to differentiate dysferlinopathy from other genetic muscle diseases. Neuromuscul Disord 34:54–60 10.1016/j.nmd.2023.11.004 38007344

[CR16] Bolano-Diaz, Reyngoudt CF, Wilson H, James IJ, Smith MK, Caldas de Almeida Araujo FE, Gordish-Dressman E, Hilsden H, Rufibach LE H, Wallace D et al (2025) Modeling of dysferlinopathy (LGMDR2) progression: a longitudinal fat fraction analysis. Neurol Genet 11:e200283 10.1212/NXG.0000000000200283 PMC12310143 40740171

[CR17] Bouchard C, Tremblay JP (2023) Portrait of dysferlinopathy: diagnosis and development of therapy. J Clin Med 12:6011 10.3390/jcm12186011 PMC10531777 37762951

[CR18] Chawla T, Baskar D, Polavarapu K, Preethish-Kumar V, Nashi S, Vengalil S, Kulanthaivelu K, Saini J, Huddar A, Unnikrishnan G et al (2025) Muscle MRI pattern in dysferlinopathy and its correlation with dysferlin gait. Ann Indian Acad Neurol 28:182–188 10.4103/aian.aian_987_24 PMC12049224 40079678

[CR19] Consalvi S, Mozzetta C, Bettica P, Germani M, Fiorentini F, Del Bene F, Rocchetti M, Leoni F, Monzani V, Mascagni P et al (2013) Preclinical studies in the mdx mouse model of Duchenne muscular dystrophy with the histone deacetylase inhibitor givinostat. Mol Med 19:79–87 10.2119/molmed.2013.00011 PMC3667212 23552722

[CR20] Contreras O, Cruz-Soca M, Theret M, Soliman H, Tung LW, Groppa E, Rossi FM, Brandan E (2019) Cross-talk between TGF-beta and PDGFRalpha signaling pathways regulates the fate of stromal fibro-adipogenic progenitors. J Cell Sci 132:jcs232157 10.1242/jcs.232157 31434718

[CR21] Cramer GD, Darby SA, Huff TG, Cummings SA, Mensching R, Sistak K, ScienceDirect (2014) Clinical anatomy of the spine, spinal cord, and ANS. Elsevier, St. Louis

[CR22] de Winter TJJ, Nusse R (2021) Running against the Wnt: how Wnt/beta-catenin suppresses adipogenesis. Front Cell Dev Biol 9:627429 10.3389/fcell.2021.627429 PMC7900430 33634128

[CR23] Defour A, Van der Meulen JH, Bhat R, Bigot A, Bashir R, Nagaraju K, Jaiswal JK (2014) Dysferlin regulates cell membrane repair by facilitating injury-triggered acid sphingomyelinase secretion. Cell Death Dis 5:e1306 10.1038/cddis.2014.272 PMC4079937 24967968

[CR24] Díaz J, Woudt L, Suazo L, Garrido C, Caviedes P, CÁrdenas AM, Castiglioni C, Bevilacqua JA (2016) Broadening the imaging phenotype of dysferlinopathy at different disease stages. Muscle Nerve 54:203–210 10.1002/mus.25045 26800485

[CR25] Diaz-Manera J, Fernandez-Torron RJLL, James MK, Mayhew A, Smith FE, Moore UR, Blamire AM, Carlier PG, Rufibach L et al (2018) Muscle MRI in patients with dysferlinopathy: pattern recognition and implications for clinical trials. J Neurol Neurosurg Psychiatry 89:1071–1081 10.1136/jnnp-2017-317488 PMC6166612 29735511

[CR26] Dobin A, Davis CA, Schlesinger F, Drenkow J, Zaleski C, Jha S, Batut P, Chaisson M, Gingeras TR (2013) STAR: ultrafast universal RNA-seq aligner. Bioinformatics 29:15–21 10.1093/bioinformatics/bts635 PMC3530905 23104886

[CR27] Ewels P, Magnusson M, Lundin S, Kaller M (2016) MultiQC: summarize analysis results for multiple tools and samples in a single report. Bioinformatics 32:3047–3048 10.1093/bioinformatics/btw354 PMC5039924 27312411

[CR28] Fanin M, Angelini C (2002) Muscle pathology in dysferlin deficiency. Neuropathol Appl Neurobiol 28:461–470 10.1046/j.1365-2990.2002.00417.x 12445162

[CR29] Fu Z, Wang T, Zhang C, Qi T, Chen Y, Yang J, Yang H, Yan B, Gong B, Lu W, Luo S, Liu Y, Sun L, Jiang H, Chen B, Zhang Z, Liu X, Wang Y (2026) Gmppb-mutant mice exhibit dystroglycanopathy symptoms that are rescued with GSK3β inhibition or AAV-mediated GMPPB gene replacement. Nat Commun 17:5340 10.1038/s41467-026-71524-7 PMC13272650 41957353

[CR30] Gallardo E, Rojas-Garcia R, de Luna N, Pou A, Brown RH Jr, Illa I (2001) Inflammation in dysferlin myopathy: immunohistochemical characterization of 13 patients. Neurology 57:2136–2138 10.1212/wnl.57.11.2136 11739845

[CR31] Gati I, Danielsson O, Gunnarsson C, Vrethem M, Haggqvist B, Fredriksson BA, Landtblom AM (2012) Bent spine syndrome: a phenotype of dysferlinopathy or a symptomatic DYSF gene mutation carrier. Eur Neurol 67:300–302 10.1159/000336265 22517428

[CR32] Grounds MD, Terrill JR, Radley-Crabb HG, Robertson T, Papadimitriou J, Spuler S, Shavlakadze T (2014) Lipid accumulation in dysferlin-deficient muscles. Am J Pathol 184:1668–1676 10.1016/j.ajpath.2014.02.005 24685690

[CR33] Gushchina LV, Bhattacharya S, McElhanon KE, Choi JH, Manring H, Beck EX, Alloush J, Weisleder N (2017) Treatment with recombinant human MG53 protein increases membrane integrity in a mouse model of limb girdle muscular dystrophy 2B. Mol Ther 25:2360–2371 10.1016/j.ymthe.2017.06.025 PMC5628796 28750735

[CR34] Haberland M, Carrer M, Mokalled MH, Montgomery RL, Olson EN (2010) Redundant control of adipogenesis by histone deacetylases 1 and 2. J Biol Chem 285:14663–14670 10.1074/jbc.M109.081679 PMC2863240 20190228

[CR35] Hesse B, Fischer MS, Schilling N (2010) Distribution pattern of muscle fiber types in the perivertebral musculature of two different sized species of mice. Anat Rec 293:446–463 10.1002/ar.21090 20169566

[CR36] Ho M, Gallardo E, McKenna-Yasek D, De Luna N, Illa I, Brown RH Jr (2002) A novel, blood-based diagnostic assay for limb girdle muscular dystrophy 2B and Miyoshi myopathy. Ann Neurol 51:129–133 10.1002/ana.10080 11782994

[CR37] Hogarth MW, Defour A, Lazarski C, Gallardo E, Diaz Manera J, Partridge TA, Nagaraju K, Jaiswal JK (2019) Fibroadipogenic progenitors are responsible for muscle loss in limb girdle muscular dystrophy 2B. Nat Commun 10:2430 10.1038/s41467-019-10438-z PMC6547715 31160583

[CR38] Hogarth MW, Kurukunda MP, Ismat K, Uapinyoying P, Jaiswal JK (2025) Exploring the therapeutic potential of fibroadipogenic progenitors in muscle disease. J Neuromuscul Dis 12:22143602241298545 10.1177/22143602241298545 PMC11949306 39973455

[CR39] Hogarth MW, Uapinyoying P, Mazala DAG, Jaiswal JK (2022) Pathogenic role and therapeutic potential of fibro-adipogenic progenitors in muscle disease. Trends Mol Med 28:8–11 10.1016/j.molmed.2021.10.003 PMC11969197 34750068

[CR40] Hornsey MA, Laval SH, Barresi R, Lochmuller H, Bushby K (2013) Muscular dystrophy in dysferlin-deficient mouse models. Neuromuscul Disord 23:377–387 10.1016/j.nmd.2013.02.004 23473732

[CR41] Horrigan J, Gomes TB, Snape M, Nikolenko N, McMorn A, Evans S, Yaroshinsky A, Della Pasqua O, Oosterholt S, Lochmuller H (2020) A phase 2 study of AMO-02 (Tideglusib) in congenital and childhood-onset myotonic dystrophy type 1 (DM1). Pediatr Neurol 112:84–93 10.1016/j.pediatrneurol.2020.08.001 32942085

[CR42] Jho EH, Zhang T, Domon C, Joo CK, Freund JN, Costantini F (2002) Wnt/beta-catenin/Tcf signaling induces the transcription of Axin2, a negative regulator of the signaling pathway. Mol Cell Biol 22:1172–1183 10.1128/MCB.22.4.1172-1183.2002 PMC134648 11809808

[CR43] Jordan B, Eger K, Koesling S, Zierz S (2011) Camptocormia phenotype of FSHD: a clinical and MRI study on six patients. J Neurol 258:866–873 10.1007/s00415-010-5858-z 21165637

[CR44] Jungbluth H, Lillis S, Zhou H, Abbs S, Sewry C, Swash M, Muntoni F (2009) Late-onset axial myopathy with cores due to a novel heterozygous dominant mutation in the skeletal muscle ryanodine receptor (RYR1) gene. Neuromuscul Disord 19:344–347 10.1016/j.nmd.2009.02.005 19303294

[CR45] Kim DH, Jang DH, Jang JH (2020) Incidental severe fatty degeneration of the erector spinae in a patient with L5-S1 disc extrusion diagnosed with limb-girdle muscular dystrophy R2 dysferin-related. Diagnostics 10:530 10.3390/diagnostics10080530 PMC7459781 32751317

[CR46] Lagace DC, Nachtigal MW (2004) Inhibition of histone deacetylase activity by valproic acid blocks adipogenesis. J Biol Chem 279:18851–18860 10.1074/jbc.M312795200 14985358

[CR47] Lagger G, O’Carroll D, Rembold M, Khier H, Tischler J, Weitzer G, Schuettengruber B, Hauser C, Brunmeir R, Jenuwein T et al (2002) Essential function of histone deacetylase 1 in proliferation control and CDK inhibitor repression. EMBO J 21:2672–2681 10.1093/emboj/21.11.2672 PMC126040 12032080

[CR48] Li B, Dewey CN (2011) RSEM: accurate transcript quantification from RNA-Seq data with or without a reference genome. BMC Bioinformatics 12:323 10.1186/1471-2105-12-323 PMC3163565 21816040

[CR49] Liberzon A, Birger C, Thorvaldsdottir H, Ghandi M, Mesirov JP, Tamayo P (2015) The Molecular Signatures Database (MSigDB) hallmark gene set collection. Cell Syst 1:417–425 10.1016/j.cels.2015.12.004 PMC4707969 26771021

[CR50] Liu J, Aoki M, Illa I, Wu C, Fardeau M, Angelini C, Serrano C, Urtizberea JA, Hentati F, Hamida MB et al (1998) Dysferlin, a novel skeletal muscle gene, is mutated in Miyoshi myopathy and limb girdle muscular dystrophy. Nat Genet 20:31–36 10.1038/1682 9731526

[CR51] Llanso L, Moore U, Bolano-Diaz C, James M, Blamire AM, Carlier PG, Rufibach L, Gordish-Dressman H, Boyle G, Hilsden H et al (2023) Expanding the muscle imaging spectrum in dysferlinopathy: description of an outlier population from the classical MRI pattern. Neuromuscul Disord 33:349–357 10.1016/j.nmd.2023.02.007 36972667

[CR52] Loseth S, Voermans NC, Torbergsen T, Lillis S, Jonsrud C, Lindal S, Kamsteeg EJ, Lammens M, Broman M, Dekomien G et al (2013) A novel late-onset axial myopathy associated with mutations in the skeletal muscle ryanodine receptor (RYR1) gene. J Neurol 260:1504–1510 10.1007/s00415-012-6817-7 23329375

[CR53] Love MI, Huber W, Anders S (2014) Moderated estimation of fold change and dispersion for RNA-seq data with DESeq2. Genome Biol 15:550 10.1186/s13059-014-0550-8 PMC4302049 25516281

[CR54] Lv X, Qiu J, Hao T, Zhang H, Jiang H, Tan Y (2021) HDAC inhibitor Trichostatin A suppresses adipogenesis in 3T3-L1 preadipocytes. Aging 13:17489–17498 10.18632/aging.203238 PMC8312440 34232916

[CR55] Mahjneh I, Marconi G, Bushby K, Anderson LV, Tolvanen-Mahjneh H, Somer H (2001) Dysferlinopathy (LGMD2B): a 23-year follow-up study of 10 patients homozygous for the same frameshifting dysferlin mutations. Neuromuscul Disord 11:20–26 10.1016/s0960-8966(00)00157-7 11166162

[CR56] Marcella BM, Hockey BL, Braun JL, Whitley KC, Geromella MS, Baranowski RW, Watson CJF, Silvera S, Hamstra SI, Wasilewicz LJ et al (2024) GSK3 inhibition improves skeletal muscle function and whole-body metabolism in male mouse models of Duchenne muscular dystrophy. Nat Commun 15:10210 10.1038/s41467-024-53886-y PMC11589163 39587049

[CR57] Mariano A, Henning A, Han R (2013) Dysferlin-deficient muscular dystrophy and innate immune activation. FEBS J 280:4165–4176 10.1111/febs.12261 23527661

[CR58] Mercuri E, Pichiecchio A, Allsop J, Messina S, Pane M, Muntoni F (2007) Muscle MRI in inherited neuromuscular disorders: past, present, and future. J Magn Reson Imaging 25:433–440 10.1002/jmri.20804 17260395

[CR59] Mercuri E, Vilchez JJ, Boespflug-Tanguy O, Zaidman CM, Mah JK, Goemans N, Muller-Felber W, Niks EH, Schara-Schmidt U, Bertini E et al (2024) Safety and efficacy of givinostat in boys with Duchenne muscular dystrophy (EPIDYS): a multicentre, randomised, double-blind, placebo-controlled, phase 3 trial. Lancet Neurol 23:393–403 10.1016/S1474-4422(24)00036-X 38508835

[CR60] Meznaric M, Gonzalez-Quereda L, Gallardo E, de Luna N, Gallano P, Fanin M, Angelini C, Peterlin B, Zidar J (2011) Abnormal expression of dysferlin in skeletal muscle and monocytes supports primary dysferlinopathy in patients with one mutated allele. Eur J Neurol 18:1021–1023 10.1111/j.1468-1331.2010.03240.x 21658164

[CR61] Milan M, Pace V, Maiullari F, Chirivi M, Baci D, Maiullari S, Madaro L, Maccari S, Stati T, Marano G et al (2018) Givinostat reduces adverse cardiac remodeling through regulating fibroblasts activation. Cell Death Dis 9:108 10.1038/s41419-017-0174-5 PMC5833837 29371598

[CR62] Minetti GC, Colussi C, Adami R, Serra C, Mozzetta C, Parente V, Fortuni S, Straino S, Sampaolesi M, Di Padova M et al (2006) Functional and morphological recovery of dystrophic muscles in mice treated with deacetylase inhibitors. Nat Med 12:1147–1150 10.1038/nm1479 16980968

[CR63] Moore U, Jacobs M, Fernandez-Torron RJLLR, Smith FE, James M, Mayhew A, Rufibach L, Carlier PG, Blamire AM et al (2020) Intensive teenage activity is associated with greater muscle hyperintensity on T1W magnetic resonance imaging in adults with dysferlinopathy. Front Neurol 11:613446 10.3389/fneur.2020.613446 PMC7773023 33391171

[CR64] Mozzetta C, Sartorelli V, Steinkuhler C, Puri PL (2024) HDAC inhibitors as pharmacological treatment for Duchenne muscular dystrophy: a discovery journey from bench to patients. Trends Mol Med 30:278–294 10.1016/j.molmed.2024.01.007 PMC11095976 38408879

[CR65] Nagashima T, Chuma T, Mano Y, Goto Y, Hayashi YK, Minami N, Nishino I, Nonaka I, Takahashi T, Sawa H et al (2004) Dysferlinopathy associated with rigid spine syndrome. Neuropathology 24:341–346 10.1111/j.1440-1789.2004.00573.x 15641596

[CR66] Nagy N, Nonneman RJ, Llanga T, Dial CF, Riddick NV, Hampton T, Moy SS, Lehtimaki KK, Ahtoniemi T, Puolivali J et al (2017) Hip region muscular dystrophy and emergence of motor deficits in dysferlin-deficient Bla/J mice. Physiol Rep 5:e13173 10.14814/phy2.13173 PMC5371557 28320887

[CR67] Potter RA, Griffin DA, Sondergaard PC, Johnson RW, Pozsgai ER, Heller KN, Peterson EL, Lehtimaki KK, Windish HP, Mittal PJ et al (2018) Systemic delivery of dysferlin overlap vectors provides long-term gene expression and functional improvement for dysferlinopathy. Hum Gene Ther 29:749–762 10.1089/hum.2017.062 PMC6066196 28707952

[CR68] Pryadkina M, Lostal W, Bourg N, Charton K, Roudaut C, Hirsch ML, Richard I (2015) A comparison of AAV strategies distinguishes overlapping vectors for efficient systemic delivery of the 6.2 kb Dysferlin coding sequence. Mol Ther Methods Clin Dev 2:15009 10.1038/mtm.2015.9 PMC4445010 26029720

[CR69] Quinn CJ, Cartwright EJ, Trafford AW, Dibb KM (2024) On the role of dysferlin in striated muscle: membrane repair, t-tubules and Ca(2+) handling. J Physiol 602:1893–1910 10.1113/JP285103 38615232

[CR70] Rawls A, Diviak BK, Smith CI, Severson GW, Acosta SA, Wilson-Rawls J (2023) Pharmacotherapeutic approaches to treatment of muscular dystrophies. Biomolecules 13:1536 10.3390/biom13101536 PMC10605463 37892218

[CR71] Reggio A, De Paolis F, Bousselmi S, Cicciarelli F, Bernardini S, Rainer A, Seliktar D, Testa S, Cirillo C, Grumati P et al (2023) A 3D adipogenesis platform to study the fate of fibro/adipogenic progenitors in muscular dystrophies. Dis Model Mech 16:dmm049915 10.1242/dmm.049915 PMC10309591 37272428

[CR72] Reggio A, Rosina M, Palma A, Cerquone Perpetuini A, Petrilli LL, Gargioli C, Fuoco C, Micarelli E, Giuliani G, Cerretani M et al (2020) Adipogenesis of skeletal muscle fibro/adipogenic progenitors is affected by the WNT5a/GSK3/beta-catenin axis. Cell Death Differ 27:2921–2941 10.1038/s41418-020-0551-y PMC7492278 32382110

[CR73] Ross SE, Hemati N, Longo KA, Bennett CN, Lucas PC, Erickson RL, MacDougald OA (2000) Inhibition of adipogenesis by Wnt signaling. Science 289:950–953 10.1126/science.289.5481.950 10937998

[CR74] Rothenberg ML, Nelson AR, Hande KR (1999) New drugs on the horizon: matrix metalloproteinase inhibitors. Stem Cells 17:237–240 10.1002/stem.170237 10437989

[CR75] Sarkozy A, Deschauer M, Carlier RY, Schrank B, Seeger J, Walter MC, Schoser B, Reilich P, Leturq F, Radunovic A et al (2012) Muscle MRI findings in limb girdle muscular dystrophy type 2L. Neuromuscul Disord 22(Suppl 2):S122–S129 10.1016/j.nmd.2012.05.012 22980763

[CR76] Schuenke MD, Vleeming A, Van Hoof T, Willard FH (2012) A description of the lumbar interfascial triangle and its relation with the lateral raphe: anatomical constituents of load transfer through the lateral margin of the thoracolumbar fascia. J Anat 221:568–576 10.1111/j.1469-7580.2012.01517.x PMC3512280 22582887

[CR77] Selcen D, Stilling G, Engel AG (2001) The earliest pathologic alterations in dysferlinopathy. Neurology 56:1472–1481 10.1212/wnl.56.11.1472 11402103

[CR78] Seror P, Krahn M, Laforet P, Leturcq F, Maisonobe T (2008) Complete fatty degeneration of lumbar erector spinae muscles caused by a primary dysferlinopathy. Muscle Nerve 37:410–414 10.1002/mus.20910 17932988

[CR79] Shtutman M, Zhurinsky J, Simcha I, Albanese C, D’Amico M, Pestell R, Ben-Ze’ev A (1999) The cyclin D1 gene is a target of the beta-catenin/LEF-1 pathway. Proc Natl Acad Sci USA 96:5522–5527 10.1073/pnas.96.10.5522 PMC21892 10318916

[CR80] Silva AMS, Coimbra-Neto AR, Souza PVS, Winckler PB, Goncalves MVM, Cavalcanti EBU, Carvalho A, Sobreira C, Camelo CG, Mendonca RDH et al (2019) Clinical and molecular findings in a cohort of ANO5-related myopathy. Ann Clin Transl Neurol 6:1225–1238 10.1002/acn3.50801 PMC6649425 31353849

[CR81] Sreetama SC, Chandra G, Van der Meulen JH, Ahmad MM, Suzuki P, Bhuvanendran S, Nagaraju K, Hoffman EP, Jaiswal JK (2018) Membrane stabilization by modified steroid offers a potential therapy for muscular dystrophy due to dysferlin deficit. Mol Ther 26:2231–2242 10.1016/j.ymthe.2018.07.021 PMC6127637 30166241

[CR82] Straub V, Murphy A, Udd B (2018) 229th ENMC international workshop: limb girdle muscular dystrophies-nomenclature and reformed classification Naarden, the Netherlands, 17-19 March 2017. Neuromuscul Disord 28:702–710 10.1016/j.nmd.2018.05.007 30055862

[CR83] Telles E, Seto E (2012) Modulation of cell cycle regulators by HDACs. Front Biosci 4:831–839 10.2741/s303 PMC3990255 22202094

[CR84] Tominaga K, Tominaga N, Williams EO, Rufibach L, Schowel V, Spuler S, Viswanathan M, Guarente LP (2022) 4-Phenylbutyrate restores localization and membrane repair to human dysferlin mutations. iScience 25:103667 10.1016/j.isci.2021.103667 PMC8741482 35028538

[CR85] Triki M, Lapierre M, Cavailles V, Mokdad-Gargouri R (2017) Expression and role of nuclear receptor coregulators in colorectal cancer. World J Gastroenterol 23:4480–4490 10.3748/wjg.v23.i25.4480 PMC5504363 28740336

[CR86] Uezumi A, Fukada S, Yamamoto N, Takeda S, Tsuchida K (2010) Mesenchymal progenitors distinct from satellite cells contribute to ectopic fat cell formation in skeletal muscle. Nat Cell Biol 12:143–152 10.1038/ncb2014 20081842

[CR87] Valentin S, Licka TF (2015) Comparative need for spinal stabilisation between quadrupedal and bipedal locomotion. Comp Exerc Physiol 11:95–105 10.3920/CEP150008 PMC5614437 28959357

[CR88] van de Wetering M, Sancho E, Verweij C, de Lau W, Oving I, Hurlstone A, van der Horn K, Batlle E, Coudreuse D, Haramis AP et al (2002) The beta-catenin/TCF-4 complex imposes a crypt progenitor phenotype on colorectal cancer cells. Cell 111:241–250 10.1016/s0092-8674(02)01014-0 12408868

[CR89] Vaziri C, Faller DV (1996) Down-regulation of platelet-derived growth factor receptor expression during terminal differentiation of 3T3-L1 pre-adipocyte fibroblasts. J Biol Chem 271:13642–13648 10.1074/jbc.271.23.13642 8662875

[CR90] Walter MC, Reilich P, Thiele S, Schessl J, Schreiber H, Reiners K, Kress W, Muller-Reible C, Vorgerd M, Urban P et al (2013) Treatment of dysferlinopathy with deflazacort: a double-blind, placebo-controlled clinical trial. Orphanet J Rare Dis 8:26 10.1186/1750-1172-8-26 PMC3617000 23406536

[CR91] Wang M, Weng WC, Stock L, Lindquist D, Martinez A, Gourdon G, Timchenko N, Snape M, Timchenko L (2019) Correction of glycogen synthase kinase 3beta in myotonic dystrophy 1 reduces the mutant RNA and improves postnatal survival of DMSXL mice. Mol Cell Biol 39:e00155–19 10.1128/MCB.00155-19 PMC6791656 31383751

[CR92] Ward SR, Tomiya A, Regev GJ, Thacker BE, Benzl RC, Kim CW, Lieber RL (2009) Passive mechanical properties of the lumbar multifidus muscle support its role as a stabilizer. J Biomech 42:1384–1389 10.1016/j.jbiomech.2008.09.042 PMC2752430 19457491

[CR93] Witting N, Andersen LK, Vissing J (2016) Axial myopathy: an overlooked feature of muscle diseases. Brain 139:13–22 10.1093/brain/awv332 26667281

[CR94] Wosczyna MN, Rando TA (2018) A muscle stem cell support group: coordinated cellular responses in muscle regeneration. Dev Cell 46:135–143 10.1016/j.devcel.2018.06.018 PMC6075730 30016618

[CR95] Xu Z, Ande SR, Mishra S (2013) Temporal analysis of protein lysine acetylation during adipocyte differentiation. Adipocyte 2:33–40 10.4161/adip.21916 PMC3661132 23700550

[CR96] Yang Loureiro Z, Joyce S, DeSouza T, Solivan-Rivera J, Desai A, Skritakis P, Yang Q, Ziegler R, Zhong D, Nguyen TT et al (2023) Wnt signaling preserves progenitor cell multipotency during adipose tissue development. Nat Metab 5:1014–1028 10.1038/s42255-023-00813-y PMC10290956 37337125

[CR97] Yasa J, Reed CE, Bournazos AM, Evesson FJ, Pang I, Graham ME, Wark JR, Nijagal B, Kwan KH, Kwiatkowski T et al (2023) Minimal expression of dysferlin prevents development of dysferlinopathy in dysferlin exon 40a knockout mice. Acta Neuropathol Commun 11:15 10.1186/s40478-022-01473-x PMC9847081 36653852

[CR98] Yu G, Wang LG, Han Y, He QY (2012) clusterProfiler: an R package for comparing biological themes among gene clusters. OMICS 16:284–287 10.1089/omi.2011.0118 PMC3339379 22455463

[CR99] Zhang J, Wang E, Li Q, Peng Y, Jin H, Naseem S, Sun B, Park S, Choi S, Li X (2024) GSK3 regulation Wnt/beta-catenin signaling affects adipogenesis in bovine skeletal muscle fibro/adipogenic progenitors. Int J Biol Macromol 275:133639 10.1016/j.ijbiomac.2024.133639 38969042

[CR100] Zhang P, Zhou M, Zhu Y, Xie J, Huo Z, Zhang D, Li P, Guo J, Li G, Li X et al (2025) The glycogen synthase kinase-3 inhibitor CHIR99021 reduces fatty infiltration and muscle atrophy after rotator cuff tears: an in vitro experiment and in vivo mouse model. Am J Sports Med 53:1184–1194 10.1177/03635465251319549 PMC12084656 39989144

