# Supplementary material for: FAP adipogenesis drives early spinal muscle loss in dysferlinopathy and reveals a therapeutic target
Source: EMBO Mol Med. 2026 Oct 12;18(10):4034–58. doi: 10.1038/s44321-026-00514-5 (PMC13658238; doi:10.1038/s44321-026-00514-5)
Supplement: Supplementary file 1 [file 44321_2026_514_MOESM13_ESM.pdf]

Expanded View Figures

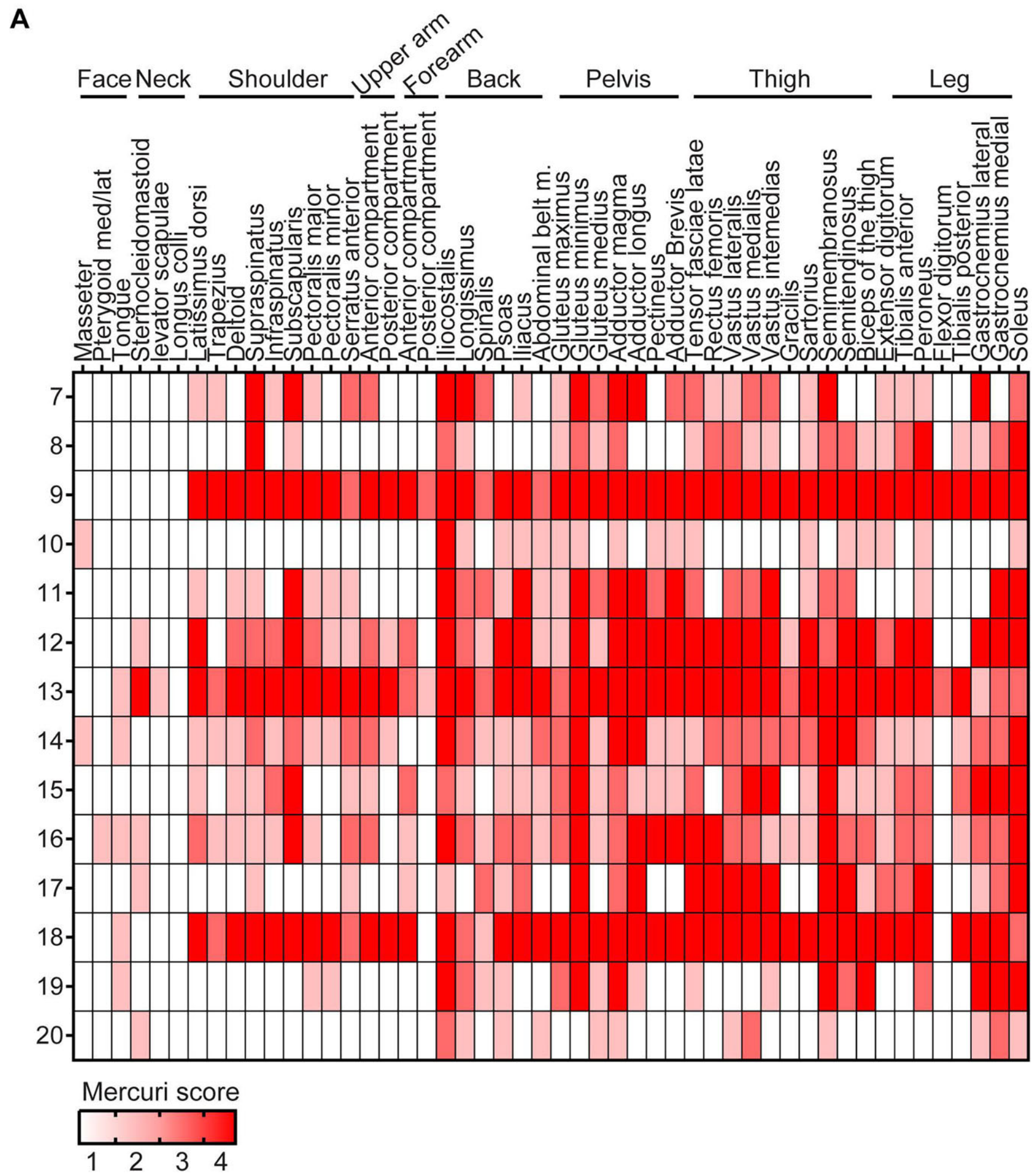

Figure EV1. Heterogeneous manifestation of muscle loss in the dysferlinopathic patient cohort.

Heat map showing the degree of fatty change observed on MRI T1-weighted images of the various body muscles imaged in the dysferlinopathic patient cohort that underwent high-resolution whole-body MRI. The numbers denote the patient ID as listed in Table 1.

© The Author(s)

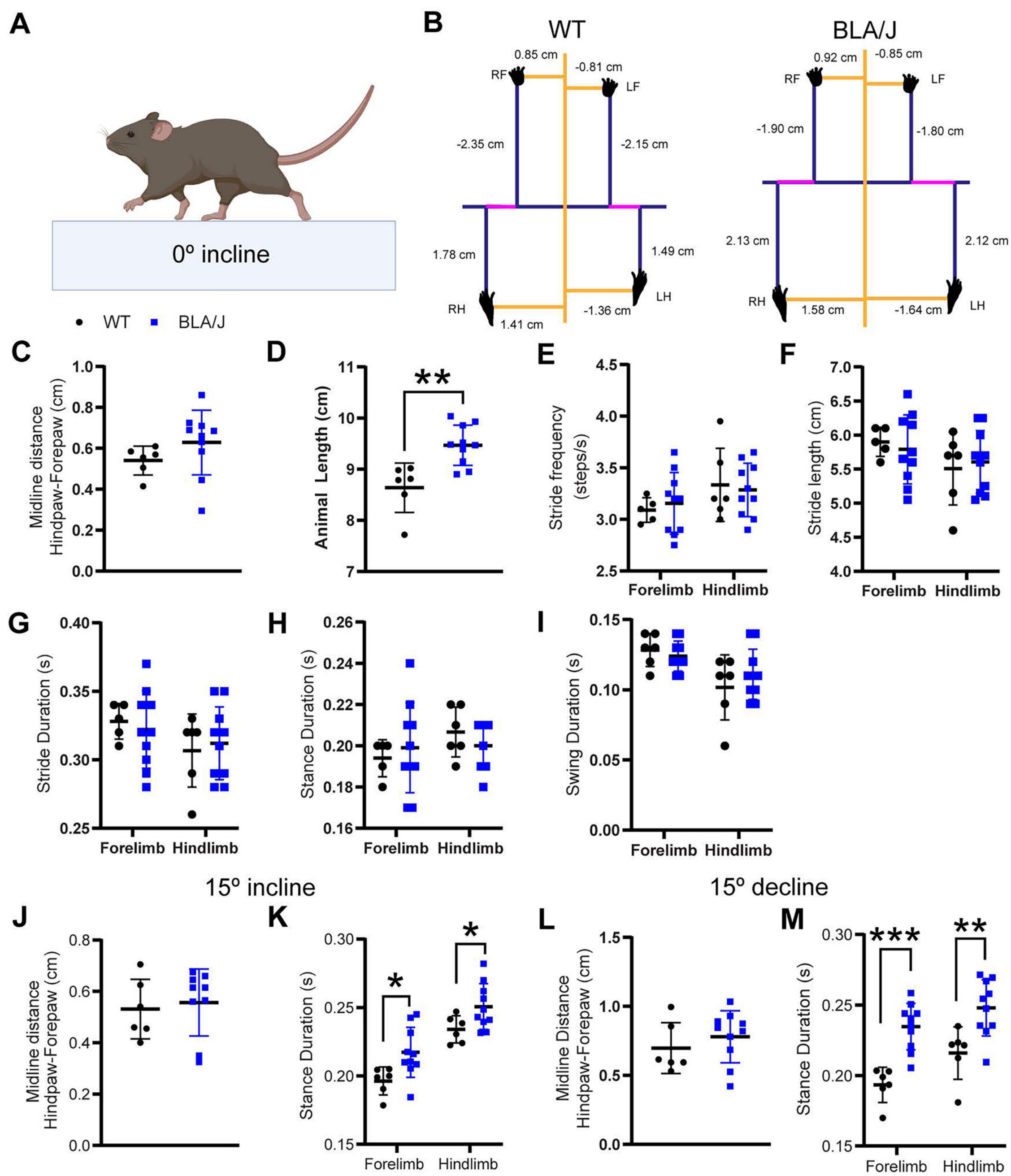

Figure EV2. Analysis of dysferlinopathic mouse gait during horizontal locomotion.

(**A**) Schematic showing a mouse running on a flat treadmill. (**B**) Representative posture plot generated by Digigait software for a mouse walking on the treadmill at a speed of 18 cm/s. The golden lines mark the midline and axial distance of the forepaw and hindpaw. The magenta line indicates the difference between the midline distance of the hindlimb and the midline distance of the forelimb. RF right forelimb, LF left forelimb, RH right hindlimb, LH left hindlimb. (**C–I**) Plots showing quantification of (**C**) midline distance between forepaw and hindpaw, (**D**) animal length, (**E**) stride frequency, (**F**) stride length, (**G**) stride duration, (**H**) stride duration, and (**I**) swing duration. Gait parameters are presented for 8-month-old female BLA/J ($n = 10$) and WT ($n = 6$) mice, with results included for both (left and right) sides. (**J**, **K**) Plots showing (**J**) midline distance, and (**K**) stance duration for BLA/J and WT mice walking on a 15° incline. (**L**, **M**) Plots showing (**L**) midline distance, and (**M**) stance duration from mice walking on a 15° decline. The values are mean ± SD. *$P < 0.05$, **$P < 0.01$, ***$P < 0.001$, ****$P < 0.0001$ by T test (when normally distributed) or Mann–Whitney test.

© The Author(s)

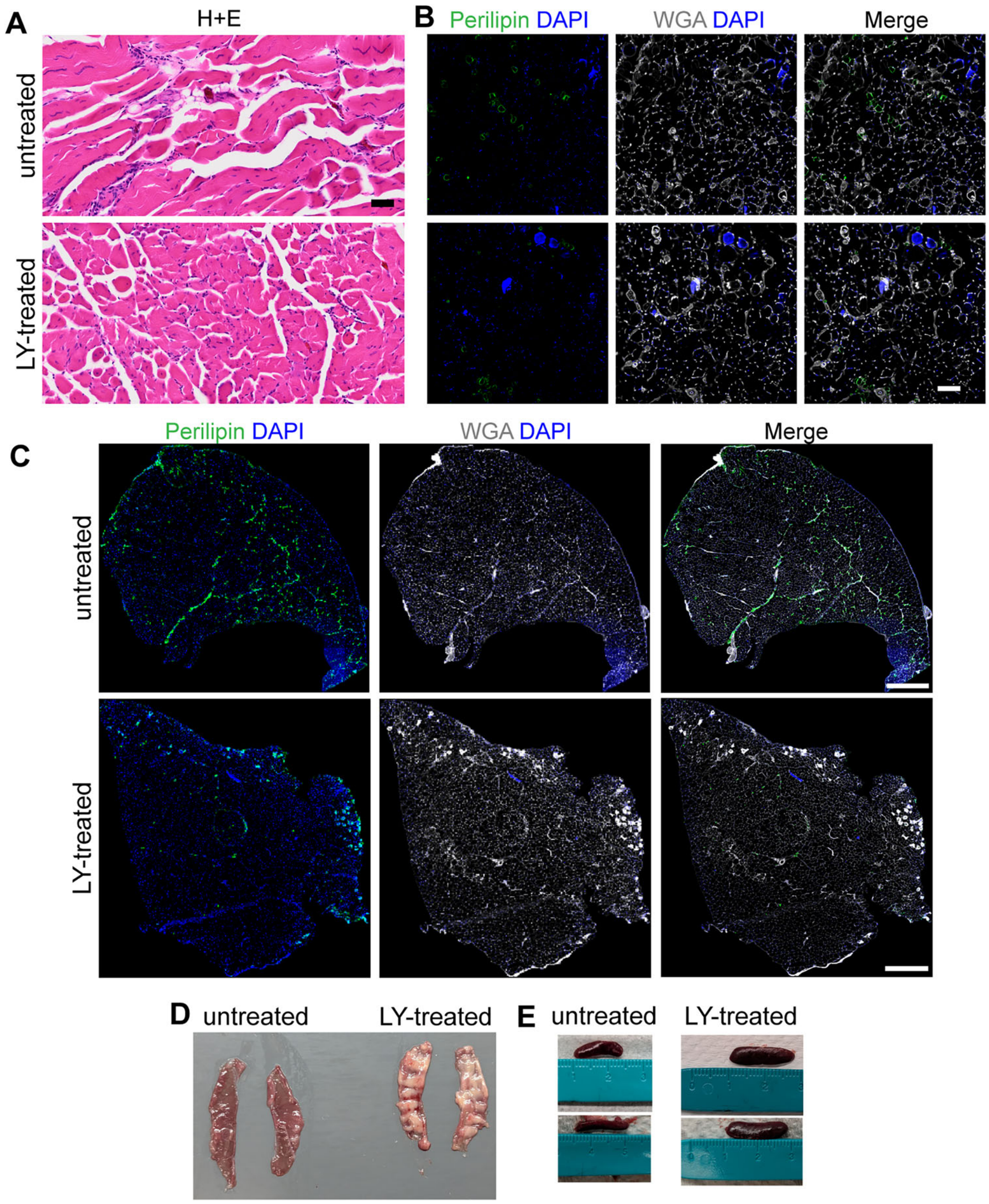

Figure EV3. Effects of LY2090314 treatment on spinal, limb, diaphragm muscles, and spleen of dysferlinopathic mice.

(**A**) H&E and (**B**) immunofluorescence images of spinal muscles from a pair of untreated and LY2090314-treated BLA/J mice (independent of those in Fig. 9B). Scale bar = 50 µm. (**C**) Immunofluorescence images of the entire cross-section of the TA muscle from untreated and LY2090314-treated BLA/J mice. Scale bar = 500 µm. Images of (**D**) Diaphragm and (**E**) Spleen from a pair of untreated and LY2090314-treated BLA/J mice.
