# Supplementary material for: FAP adipogenesis drives early spinal muscle loss in dysferlinopathy and reveals a therapeutic target
Source: EMBO Mol Med. 2026 Oct 12;18(10):4034–58. doi: 10.1038/s44321-026-00514-5 (PMC13658238; doi:10.1038/s44321-026-00514-5)
Supplement: Supplementary file 2 [file 44321_2026_514_MOESM2_ESM.pdf]

Appendix

Table of Content

- **Appendix Table S1**

Appendix Table S1: Statistical Analysis Corresponding to the Data Presented in Figures

Figure	Panel	Sample Size (n = biological replicates)	Statistical Test	p-value
**1**	G	20	Simple Linear Regression	0.0274
**1**	H	20	Simple Linear Regression	0.0023
**2**	F	WT: 3	unpaired t test	0.0002
		BlaJ: 3		
**2**	G	WT: 3	unpaired t test	0.0063
		BlaJ: 3		
**2**	H	6-month spine: 4	Two-way ANOVA	0.0249
		6-month quad: 4	Holm-Sidak's multiple comparisons test	6 mo. spine vs. 6 mo. quad: 0.0493
		8-month spine: 4	Holm-Sidak's multiple comparisons test	8 mo. spine vs. 8 mo. quad: 0.0151
		8-month quad: 4	Holm-Sidak's multiple comparisons test	10 mo. spine vs. 10 mo. quad: 0.0025
		10-month spine: 4	Holm-Sidak's multiple comparisons test	6 mo. spine vs. 8 mo. spine: 0.0150
		10-month quad: 4	Holm-Sidak's multiple comparisons test	6 mo. spine vs. 10 mo. spine: 0.0028
		22-month spine: 4	Holm-Sidak's multiple comparisons test	6 mo. spine vs. 22 mo. spine: 0.0079
		22-month quad: 4	Holm-Sidak's multiple comparisons test	6 mo. quad vs. 10 mo. quad: 0.0330
			Holm-Sidak's multiple comparisons test	6 mo. quad vs. 22 mo. quad: 0.0027
**3**	D	WT: 6	unpaired t test	0.0033
		BlaJ: 9		
**3**	E	Forelimb WT: 6	unpaired t test	0.0511
		Forelimb BlaJ: 10		
		Hindlimb WT: 6	unpaired t test	0.0347
		Hindlimb BlaJ: 10		
**3**	F	Forelimb WT: 6	unpaired t test	0.0332
		Forelimb BlaJ: 10		
		Hindlimb WT: 6	unpaired t test	0.0263
		Hindlimb BlaJ: 10		
**3**	G	Forelimb WT: 6	unpaired t test	0.0386
		Forelimb BlaJ: 10		
		Hindlimb WT: 6	unpaired t test	0.0268
		Hindlimb BlaJ: 10		
**3**	H	Forelimb WT: 6	unpaired t test	0.3434
		Forelimb BlaJ: 10		
		Hindlimb WT: 5	Mann-Whitney test	0.0736
		Hindlimb BlaJ: 10		
**3**	K	WT: 6	unpaired t test	0.0191
		BlaJ: 9		
**3**	L	Forelimb WT: 6	unpaired t test	<0.0001
		Forelimb BlaJ: 10		
		Hindlimb WT: 6	unpaired t test	0.0052
		Hindlimb BlaJ: 10		

**3**	M	Forelimb WT: 6	unpaired t test	<0.0001
		Forelimb BlaJ: 10		
		Hindlimb WT: 6	unpaired t test	0.0031
		Hindlimb BlaJ: 10		
**3**	N	Forelimb WT: 6	unpaired t test	<0.0001
		Forelimb BlaJ: 10		
		Hindlimb WT: 6	unpaired t test	0.0031
		Hindlimb BlaJ: 10		
**3**	O	Forelimb WT: 6	unpaired t test	0.001
		Forelimb BlaJ: 10		
		Hindlimb WT: 6	unpaired t test	0.009
		Hindlimb BlaJ: 10		
**5**	E	6-month multifidus: 3	Two-way ANOVA	0.0041
		6-month longissimus: 3	Holm-Sidak's multiple comparisons test	6 mo. Iliocostalis vs. 8 mo. Iliocostalis: 0.0524
		6-month iliocostalis: 3	Holm-Sidak's multiple comparisons test	6 mo. Iliocostalis vs. 10 mo. Iliocostalis: 0.0005
		8-month multifidus: 3		
		8-month longissimus: 3		
		8-month iliocostalis: 3		
		10-month multifidus: 3		
		10-month longissimus: 3		
		10-month iliocostalis: 3		
**5**	F	6-month multifidus: 3	Two-way ANOVA	<0.0001
		6-month longissimus: 3	Holm-Sidak's multiple comparisons test	6 mo. Iliocostalis vs. 8 mo. Iliocostalis: 0.0278
		6-month iliocostalis: 3	Holm-Sidak's multiple comparisons test	6 mo. Iliocostalis vs. 10 mo. Iliocostalis: 0.0140
		8-month multifidus: 3	Holm-Sidak's multiple comparisons test	8 mo. Iliocostalis vs. 10 mo. Iliocostalis: 0.0415
		8-month longissimus: 3	Holm-Sidak's multiple comparisons test	6 mo. Longissimus vs. 10 mo. Longissimus: 0.0278
		8-month iliocostalis: 3	Holm-Sidak's multiple comparisons test	8 mo. Longissimus vs. 10 mo. Longissimus: 0.0479
		10-month multifidus: 3		
		10-month longissimus: 3		
		10-month iliocostalis: 3		
**6**	C	WT: 3	unpaired t test	<0.0001
		BlaJ: 3		
**6**	E	Spine: 4	unpaired t test	<0.0001
		Quad: 4		
**7**	B	WT: 4	Wald and Benjamini Hochberg test	<0.05
		BlaJ: 4		
**7**	H	DMSO: 4	Mann-Whitney test	DMSO vs. MMP inhibitor: 0.0286
		MMP inhibitor: 4		DMSO vs. HDAC inhibitor: 0.0286
		HDAC inhibitor: 4		DMSO vs. GSK3 inhibitor: 0.0286
		GSK3 inhibitor: 4		
**8**	B	*axin2* WT+DMSO: 3	unpaired t test	WT+DMSO vs. BlaJ+DMSO: 0.0368

		axin2 BlaJ+DMSO: 3	unpaired t test	BlaJ+DMSO vs. BlaJ+LY: 0.0037
		axin2 BlaJ+LY: 3		
		Ccnd2 WT+DMSO: 3	unpaired t test	WT+DMSO vs. BlaJ+DMSO: 0.0009
		Ccnd2 BlaJ+DMSO: 3	unpaired t test	BlaJ+DMSO vs. BlaJ+LY: 0.1371
		Ccnd2 BlaJ+LY: 3		
		Wnt1 WT+DMSO: 3	unpaired t test	WT+DMSO vs. BlaJ+DMSO: 0.1201
		Wnt1 BlaJ+DMSO: 3	unpaired t test	BlaJ+DMSO vs. BlaJ+LY: 0.0143
		Wnt1 BlaJ+LY: 3		
		Wnt5a WT+DMSO: 3	unpaired t test	WT+DMSO vs. BlaJ+DMSO: 0.8073
		Wnt5a BlaJ+DMSO: 3	unpaired t test	BlaJ+DMSO vs. BlaJ+LY: 0.0231
		Wnt5a BlaJ+LY: 3		
		Wnt5b WT+DMSO: 3	unpaired t test	WT+DMSO vs. BlaJ+DMSO: 0.0142
		Wnt5b BlaJ+DMSO: 3	unpaired t test	BlaJ+DMSO vs. BlaJ+LY: 0.0108
		Wnt5b BlaJ+LY: 3		
		Wnt10a WT+DMSO: 3	unpaired t test	WT+DMSO vs. BlaJ+DMSO: 0.0139
		Wnt10a BlaJ+DMSO: 3	unpaired t test	BlaJ+DMSO vs. BlaJ+LY: 0.0012
		Wnt10a BlaJ+LY: 3		
**8**	D	active beta-catenin/HSP90 WT+DMSO: 3	unpaired t test	WT+DMSO vs. BlaJ+DMSO: 0.0402
		active beta-catenin/HSP90 BlaJ+DMSO: 3	unpaired t test	BlaJ+DMSO vs. BlaJ+LY: 0.0274
		active beta-catenin/HSP90 BlaJ+LY: 3		
		active beta-catenin/total beta-catenin WT+DMSO: 3	unpaired t test	WT+DMSO vs. BlaJ+DMSO: 0.0095
		active beta-catenin/total beta-catenin BlaJ+DMSO: 3	unpaired t test	BlaJ+DMSO vs. BlaJ+LY: 0.0309
		active beta-catenin/total beta-catenin BlaJ+LY: 3		
		p-C-Jun/C-Jun WT+DMSO: 3	unpaired t test	WT+DMSO vs. BlaJ+DMSO: 0.2344
		p-C-Jun/C-Jun BlaJ+DMSO: 3	unpaired t test	BlaJ+DMSO vs. BlaJ+LY: 0.1171
		p-C-Jun/C-Jun BlaJ+LY: 3		
		pGSK3beta(Ser9)/GSK3beta WT+DMSO: 3	unpaired t test	WT+DMSO vs. BlaJ+DMSO: 0.0587
		pGSK3beta(Ser9)/GSK3beta BlaJ+DMSO: 3	unpaired t test	BlaJ+DMSO vs. BlaJ+LY: 0.1664
		pGSK3beta(Ser9)/GSK3beta BlaJ+LY: 3		
		pGSK3beta/3alpha (Tyr216/Tyr279)/GSK3beta WT+DMSO: 3	unpaired t test	WT+DMSO vs. BlaJ+DMSO: 0.7931

		pGSK3beta/3alpha (Tyr216/Tyr279)/GSK3beta BlaJ+DMSO: 3	unpaired t test	BlaJ+DMSO vs. BlaJ+LY: 0.0002
		pGSK3beta/3alpha (Tyr216/Tyr279)/GSK3beta BlaJ+LY: 3		
		PPARy/HSP90 WT+DMSO: 3	unpaired t test	WT+DMSO vs. BlaJ+DMSO: <0.0001
		PPARy/HSP90 BlaJ+DMSO: 3	unpaired t test	BlaJ+DMSO vs. BlaJ+LY: <0.0001
		PPARy/HSP90 BlaJ+LY: 3		
		Total beta-catenin/HSP90 WT+DMSO: 3	unpaired t test	WT+DMSO vs. BlaJ+DMSO: 0.6222
		Total beta-catenin/HSP90 BlaJ+DMSO: 3	unpaired t test	BlaJ+DMSO vs. BlaJ+LY: 0.1239
		Total beta-catenin/HSP90 BlaJ+LY: 3		
		Total GSK3beta/HSP90 WT+DMSO: 3	unpaired t test	WT+DMSO vs. BlaJ+DMSO: 0.3609
		Total GSK3beta/HSP90 BlaJ+DMSO: 3	unpaired t test	BlaJ+DMSO vs. BlaJ+LY: 0.9574
		Total GSK3beta/HSP90/HSP90 BlaJ+LY: 3		
**9**	D	Control: 8	unpaired t test	0.0027
		LY: 6		
**9**	G	Control: 3	unpaired t test	0.048
		LY: 3		
