# Supplementary material for: FAP adipogenesis drives early spinal muscle loss in dysferlinopathy and reveals a therapeutic target
Source: EMBO Mol Med. 2026 Oct 12;18(10):4034–58. doi: 10.1038/s44321-026-00514-5 (PMC13658238; doi:10.1038/s44321-026-00514-5)
Supplement: Supplementary file 3 [file 44321_2026_514_MOESM3_ESM.pdf]

EMBO Molecular Medicine

FAP Adipogenesis Drives Early Spinal Muscle Loss in Dysferlinopathy and Reveals a Therapeutic Target

Young Jae Moon, Nikki McCormack, Thomas Diehn, Stephen Lin, Katia Staedler, Surajit Bhattacharya, Daniel Bittel, Paul Wang, Marshall Hogarth, Jong-Sung Oh, Tae Sik Goh, Ji Hong Min, Tanya Stojkovic, Isabelle Ackermann-Bonan, and Jyoti Jaiswal

Corresponding author: Jyoti Jaiswal (jkjaiswal@childrensnational.org)

Review Timeline:

Submission Date:	9th Oct 25
Editorial Decision:	20th Nov 25
Revision Received:	14th Jun 26
Editorial Decision:	1st Jul 26
Revision Received:	20th Aug 26
Accepted:	21st Aug 26

Editor: Zeljko Durdevic

Transaction Report:

(Note: With the exception of the correction of typographical or spelling errors that could be a source of ambiguity, letters and reports are not edited. Depending on transfer agreements, referee reports obtained elsewhere may or may not be included in this compilation. Referee reports are anonymous unless the Referee chooses to sign their reports.)

20th Nov 2025

Dear Prof. Jaiswal,

Thank you for the submission of your manuscript to EMBO Molecular Medicine. We have now received feedback from the three reviewers who agreed to evaluate your manuscript.

As you will see from the reports pasted below, while the referee #3 supports publication of the manuscript raising important but minor concerns, referees #1 and #2 recognize the potential interest of the study but also raise serious concerns particularly regarding the novelty, mechanistic insight and the lack of in vivo pharmacological treatments. Based on the referee reports I would like to invite major revision of the present manuscript, with the understanding that authors should provide deeper mechanistic insight and perform in vivo treatment. Considering that the revision will require extensive experimentation we think six months rather than three months would be more appropriate to provide the complete revision. If you would like to discuss further the points raised by the referees, I am available to do so via email or video. Let me know if you are interested in this option.

Further consideration of a revision that addresses reviewers' concerns in full will entail a second round of review. EMBO Molecular Medicine encourages a single round of revision only and therefore, acceptance or rejection of the manuscript will depend on the completeness of your responses included in the next, final version of the manuscript. For this reason, and to save you from any frustrations in the end, I would strongly advise against returning an incomplete revision.

We would welcome the submission of a revised version within six months for further consideration. Please let us know if you require longer to complete the revision.

I look forward to receiving your revised manuscript.

Yours sincerely,

Zeljko Durdevic

Zeljko Durdevic
Senior Editor
EMBO Molecular Medicine

*****

When submitting your revised manuscript, please carefully review the instructions that follow below. We perform an initial quality control of all revised manuscripts before re-review; failure to include requested items will delay the evaluation of your revision.

We require:

1) A .docx formatted version of the manuscript text (including legends for main figures, EV figures and tables). Please make sure that the changes are highlighted to be clearly visible.

2) Individual production quality figure files as .eps, .tif, .jpg (one file per figure). For guidance, download the 'Figure Guide PDF': (https://www.embopress.org/page/journal/17574684/authorguide#figureformat).

3) A .docx formatted letter INCLUDING the reviewers' reports and your detailed point-by-point responses to their comments. As part of the EMBO Press transparent editorial process, the point-by-point response is part of the Review Process File (RPF), which will be published alongside your paper.

4) A complete author checklist, which you can download from our author guidelines (https://www.embopress.org/page/journal/17574684/authorguide#submissionofrevisions). Please insert information in the

checklist that is also reflected in the manuscript. The completed author checklist will also be part of the RPF.

5) Please note that all corresponding authors are required to supply an ORCID ID for their name upon submission of a revised manuscript.

6) It is mandatory to include a 'Data Availability' section after the Materials and Methods. Before submitting your revision, primary datasets produced in this study need to be deposited in an appropriate public database, and the accession numbers and database listed under 'Data Availability'. Please remember to provide a reviewer password if the datasets are not yet public (see https://www.embopress.org/page/journal/17574684/authorguide#dataavailability).

In case you have no data that requires deposition in a public database, please state so in this section. Note that the Data Availability Section is restricted to new primary data that are part of this study.

7) For data quantification: please specify the name of the statistical test used to generate error bars and P values, the number (n) of independent experiments (specify technical or biological replicates) underlying each data point and the test used to calculate p-values in each figure legend. The figure legends should contain a basic description of n, P and the test applied. Graphs must include a description of the bars and the error bars (s.d., s.e.m.). See also 'Figure Legend' guidelines: https://www.embopress.org/page/journal/17574684/authorguide#figureformat

8) At EMBO Press we ask authors to provide source data for the main manuscript figures. You will receive a separate email with instructions for providing source data with your revised manuscript, including how to upload and organize the files.

9) Our journal encourages inclusion of *data citations in the reference list* to directly cite datasets that were re-used and obtained from public databases. Data citations in the article text are distinct from normal bibliographical citations and should directly link to the database records from which the data can be accessed. In the main text, data citations are formatted as follows: "Data ref: Smith et al, 2001" or "Data ref: NCBI Sequence Read Archive PRJNA342805, 2017". In the Reference list, data citations must be labeled with "[DATASET]". A data reference must provide the database name, accession number/identifiers and a resolvable link to the landing page from which the data can be accessed at the end of the reference. Further instructions are available at .

10) We replaced Supplementary Information with Expanded View (EV) Figures and Tables that are collapsible/expandable online. A maximum of 5 EV Figures can be typeset. EV Figures should be cited as 'Figure EV1, Figure EV2" etc... in the text and their respective legends should be included in the main text after the legends of regular figures.

- For the figures that you do NOT wish to display as Expanded View figures, they should be bundled together with their legends in a single PDF file called *Appendix*, which should start with a short Table of Content. Appendix figures should be referred to in the main text as: "Appendix Figure S1, Appendix Figure S2" etc.

- Additional Tables/Datasets should be labeled and referred to as Table EV1, Dataset EV1, etc. Legends have to be provided in a separate tab in case of .xls files. Alternatively, the legend can be supplied as a separate text file (README) and zipped together with the Table/Dataset file.

See detailed instructions here:

.

11) The paper explained: EMBO Molecular Medicine articles are accompanied by a summary of the articles to emphasize the major findings in the paper and their medical implications for the non-specialist reader. Please provide a draft summary of your article highlighting

- the medical issue you are addressing,

- the results obtained and

- their clinical impact.

This may be edited to ensure that readers understand the significance and context of the research. Please refer to any of our published articles for an example.

12) Author contributions: You will be asked to provide CRediT (Contributor Role Taxonomy) terms in the submission system. These replace a narrative author contribution section in the manuscript.

13) A Conflict of Interest statement should be provided in the main text.

14) Every published paper now includes a 'Synopsis' to further enhance discoverability. Synopses are displayed on the journal webpage and are freely accessible to all readers. They include a short stand first (maximum of 300 characters, including space) as well as 2-5 one-sentences bullet points that summarizes the paper. Please write the bullet points to summarize the key NEW findings. They should be designed to be complementary to the abstract - i.e. not repeat the same text. We encourage inclusion of key acronyms and quantitative information (maximum of 30 words / bullet point). Please use the passive voice. Please attach these in a separate file or send them by email, we will incorporate them accordingly.

Please also suggest a striking image or visual abstract to illustrate your article as a PNG file 550 px wide x 300-600 px high.

15) Include a Reagents and Tools Table as part of the Methods section, which can be downloaded from our author guidelines (https://www.embopress.org/page/journal/17574684/authorguide#structuredmethods)

EMBO Molecular Medicine has a "scooping protection" policy, whereby similar findings that are published by others during review or revision are not a criterion for rejection. Should you decide to submit a revised version, I do ask that you get in touch after three months if you have not completed it, to update us on the status.

***** Reviewer's comments *****

Referee #1 (Remarks for Author):

The group of Jyoti K. Jaiswal is presenting a novel work focused on investigating pharmacological targets and treatments to counteract fatty degeneration in dysferlinopathies. The authors prepared a manuscript that contains findings coming from a retrospective study that are further corroborated and rationalized in a relevant mouse model of the disease. The fact that spinal muscle degeneration is independent from gait or as a consequence of limb degeneration is of interest and open to wide array of future considerations. The histological and cell biology sections, included in this paper, are simple but of impact. Highlighting, how spinal muscle differentially experienced dystrophic damage and fat replacement is important to prioritize local treatments (pharmacological or corrective) in the future. the overall manuscript reads well and it is logically organized. Methods are sufficiently detailed ensuring data transfer and reproducibility. Although these positive evaluations, the referee has some concerns and suggestion for the authors. All are listed below:

The fact that FAPs isolated from muscles with different degeneration kinetics behave differently suggests that their source microenvironment primes/restrain them for adipogenic differentiation. It could be of interest discuss this in detail.

Linked to the previous comment, alteration in the expression of some target genes linked to the WNT- -catenin pathways may reflect relevant depletion of WNT ligands in the diseased niche. Although single-cell seq is not performed limiting broader investigation, FAPs are the major source of WNT ligands in the skeletal muscle niche. Exploring expression of WNT ligands in the bulk RNAseq may support the author decision in targeting GSK to rescue a defective pathway.

"The issues related to the potential safety of Batimastat and LY2090314, limits the clinical utility of these drugs for chronic conditions like dysferlinopathy". Regarding LY2090314, this statement is not properly true. For the sake of scientific discussion, LY2090314 has undergone rigorous evaluation in phase I/II clinical trials for AML, demonstrating a favourable safety profile and tolerability in humans. Moreover, the drug demonstrated on target activity by checking at b-catenin level. Additional tests have been discontinued due to low efficacy for the type of malignancy. These events do not preclude the repositioning of this molecules for other purposes, and of course the activation of of SAR campaigns to improve its pharmacokinetics and on-target potency. Please consider smoothing the original authors' sentence.

The MOA data for these compounds inhibiting FAP adipogenesis are not presented. It remains unclear whether the tested molecules act on the pathways identified as differentially expressed by the authors. Although this referee acknowledges the inherent difficulty in validating mechanisms associated with poorly specific HDAC inhibitors, recent evidence has shown that LY2090314 operates through two complementary circuits, both converging on β-catenin: one mediated by GSK3 inhibition and the other by the stimulation of WNT5A expression. Adding some of these mechanistic information would strength the quality of the work.

Minor
Figure 1A. the B2 label is unreadable

PDGFRa downregulation during adipogenesis has been demonstrated to occur in FAP cells using mass cytometry. The references Gao et al and Vaziri et al are too general since reported to adipose progenitor cells that are not muscle FAPs. These should be implemented to include a field-relevant bibliographic support.

Referee #2 (Comments on Novelty/Model System for Author):

Technical quality: in MRI and histology is high. It is low in the pharmacological experiments performed on FAPs. A single lipid stain and missing quantification in Fig. 7G does not suffice. So, overall, I consider it "medium"
Novelty: The paraxial fatty degeneration of muscle in muscular dystrophies has been described several times before. Unfortunately, the exciting finding shown in Fig. 7G has not been elaborated on further, the overall novelty is low.
Medical impact: If the preliminary findings shown in Fig. 7G are substantiated further in in vivo experiments the impact would be high. As shown here, the impact is low to medium.
Model system: The MRI techniques are of very high quality, as are the histological analyses.

Referee #2 (Remarks for Author):

Moon et al. provide a thorough description of MRI findings of paravertebral adipose tissue in one form of limb girdle muscular dystrophy, named dysferlinopathy or LGMD2A/R2. Dysferlinopathy is caused by mutations in the DYSF gene and follows autosomal-recessive inheritance. Correlation of MRI analysis with histological features is demonstrated at different ages in a well-known mouse model of dysferlinopathy (BLA/J).

In addition, MRI data from paraxial and thigh-muscles of 20 patients with LGMDR2 are shown. The extent of paraspinal fatty degeneration of muscle is as severe as in mice.

The authors demonstrate the in vitro reduction of lipid droplets in mouse FAPs by pharmacological intervention, the most efficient medication being the GSK3 inhibitor LY2090314.

Major questions:
With a mouse model in place in addition to functional outcome measures on a treadmill, MRI- and histological/immunofluorescent read-outs, the authors should perform in vivo pharmacological treatments. In Figure 7G, an interesting preliminary observation is depicted which should and can be substantiated much further.

It is known that paraxial muscles are significantly affected in a number of muscular dystrophies. The authors do not show comparisons between LGMDR2 and other dystrophies but cite available literature. It would be important to show which aspect of the distribution of fatty replacement of dysferlin-deficient muscle or which pharmacological intervention is specific or helps in the understanding of the underlying mechanistic pathology. This information is missing. If an intervention could be demonstrated that reverses several adipogenic tissue replacements in muscular dystrophies, this would be of very high impact.

Minor questions:

Fig. 1: Patient MRIs: Please specify the exact height of the demonstrated sections. Lumbar spine is an area of > 20 cm in lengths in humans.

Fig. 2: The quantification of data is difficult to understand. Fig. 2H - as an example: How can a quotient between fat in paraxial muscle and quadriceps be transformed into %? In addition: May spine and quadriceps muscle be mixed up?

Fig. 7G: What does n= 4 mean? Four different biological samples? Four different areas of which photos were taken? Technical replicates? Please specify

Table 1: Please be consistent in the mutation information whether the exon information should be included or not.

Referee #3 (Comments on Novelty/Model System for Author):

Relevant models used, including both human and mouse studies.

Referee #3 (Remarks for Author):

The authors present a thorough and interesting study with relevance to the dysferlin field. Clinical observations in humans with supporting functional studies in mice provides compelling evidence of early-onset spinal muscle degeneration as a feature of dysferlinopathy and identifies promising therapeutic strategies.

Some minor points for revision:
1. Introduction: Specify which dysferlin mouse model is being referred to.

2. Page 7 results Figure 1D & E labelling these images with which patient number they are would help with clarity for in-text reference to patient 4.

3. Results page 7 Figure 1/Supp figure 1 "However, irrespective of how widespread fatty degeneration was, the extent of signal in the spinal muscles was higher than the girdle muscles." While this is generally true, there are some patients (9, 13, 18) who have widespread high signal intensity across all muscles.

4. Results page 8, Figure 3 "mice showed increased stride length, stride duration, stance duration, and swing duration" swing duration was only statistically significantly increased in mice on the decline, not incline

5. Results page 8 "As the treadmill was maintained at a constant speed (15 cm/s), these delays in locomotory parameters in BLA/J mice were matched by a concomitant reduction in their forelimb and hindlimb stride frequency" is not clear to me what delays in locomotory parameters refers to in regard to the results presented

6. Figure 4A zoomed in regions are taken from different areas for different time points. Given the variability observed between different spinal muscles, this makes it more difficult to directly compare these zoomed in images

7. Figure 7A: One WT sample is a clear outlier on PCA. Was this sample included in the analyses and could the authors comment on this either way?

8. The evidence presents exciting possibilities for potential therapeutic approaches for dysferlinopathies. However, as the spinal adipogenesis appears to be an early event, can the authors comment on the possible therapeutic window for these treatments, especially considering the timeline of diagnosis for many dysferlin patients?

9. Could the authors comment on other studies that have also reported fatty accumulations in dysferlin deficient spinal muscles including Agarwal et al doi.org/10.1194/jlr.RA119000399 and Yasa et al doi.org/10.1186/s40478-022-01473-x

10. The manuscript is generally well written and easy to read but there a few minor text edits required. For example:
Page 3: and maintain calcium homeostasis
Page 3: anti-inflammatory corticosteroids or immunosuppressant
Page 9: Hemotoxylin and eosin staining of revealed
Page 9: Compared to iliocostalis, longissimus, and multifidus muscles showed relatively limited signs of myofiber damage and adipogenic degeneration of the multifidus muscles by 10 months of age. Sentence is unclear
Page 11: has helped tackled this question
Page 11: more stable erector spinae than biped human
Page 13: FAPs in these dysferlinopathic muscle niche that leads to greater adipogenic commitment of FAPs from the spinal muscles. Sentence is unclear

Dear Editor,

We thank the reviewers for their recognition of the importance of this work and constructive feedback to improve this further. We have performed additional studies resulting in the addition of two new figures that address the following two queries by the reviewers:

- Wnt signaling dysregulation in BLA/J FAPs and the mechanism of its correction by the GSK3 inhibitor LY2090314 (**Fig. 8**).
- Extending our studies to in vivo correction of adipogenic muscle loss in BLA/J mice (**Fig. 9**).

The details of these results and point-by-point response for the rest of the reviewer comments are described below and noted in the revised manuscript.

Referee #1 (Remarks for Author):

The group of Jyoti K. Jaiswal is presenting a novel work focused on investigating pharmacological targets and treatments to counteract fatty degeneration in dysferlinopathies. The authors prepared a manuscript that contains findings coming from a retrospective study that are further corroborated and rationalized in a relevant mouse model of the disease. The fact that spinal muscle degeneration is independent from gait or as a consequence of limb degeneration is of interest and open to wide array of future considerations. The histological and cell biology sections, included in this paper, are simple but of impact. Highlighting, how spinal muscle differentially experienced dystrophic damage and fat replacement is important to prioritize local treatments (pharmacological or corrective) in the future. the overall manuscript reads well and it is logically organized. Methods are sufficiently detailed ensuring data transfer and reproducibility. Although these positive evaluations, the referee has some concerns and suggestion for the authors. All are listed below:

Response: We thank the reviewer for the positive comments and suggestions to help us further improve the work, which are addressed below.

The fact that FAPs isolated from muscles with different degeneration kinetics behave differently suggests that their source microenvironment primes/restrain them for adipogenic differentiation. It could be of interest discuss this in detail.

Response: We agree with the reviewer that this is an interesting and important feature, which we had noted only briefly in our discussion section, and added further details to this discussion.

Linked to the previous comment, alteration in the expression of some target genes linked to the WNT-β-catenin pathways may reflect relevant depletion of WNT ligands in the diseased niche. Although single-cell seq is not performed limiting broader investigation, FAPs are the major source of WNT ligands in the skeletal muscle niche. Exploring expression of WNT ligands in the bulk RNAseq may support the author decision in targeting GSK to rescue a defective pathway.

Response: We agree with the reviewer regarding the importance of this pathway and had explored this in our previous work (Uapinyoying et al 2023. PMID: 37599828). We had examined the expression of WNT ligands in **Fig. 7F** and have now performed analysis of all Wnt ligands that show differential expression in our bulk seq data. This led us to identify several canonical and non-canonical Wnt ligands that show increased levels in BLA/J FAPs, including WNT1, WNT2, WNT2B, WNT4, WNT5A, WNT5B, WNT9A, WNT10A, and WNT11. Further, by qPCR analysis, we confirmed upregulation of several of these Wnt ligands and show that LY2090314 treatment effectively downregulates expression of these ligands. These data are now included in a new figure in the revised manuscript (**Fig. 8**).

"The issues related to the potential safety of Batimastat and LY2090314, limits the clinical utility of these drugs for chronic conditions like dysferlinopathy". Regarding LY2090314, this statement is not properly true. For the sake of scientific discussion, LY2090314 has undergone rigorous evaluation in phase I/II clinical trials for AML, demonstrating a favourable safety profile and tolerability in humans. Moreover, the drug demonstrated on target activity by checking at b-catenin level. Additional tests have been discontinued due to low efficacy for the type of malignancy. These events do not preclude the repositioning of this molecules for other purposes, and of course the activation of of SAR campaigns to improve its pharmacokinetics and on-

target potency. Please consider smoothing the original authors' sentence.

Response: We agree with the reviewer regarding the value of LY2090314 among the various GSK-3 inhibitors/Wnt activators. In fact, our selection of this drug was based on its published potency even at sub-nanomolar dose. Further, in an acute (28 day) phase I/II clinical trial for AML, this drug showed acceptable safety with limited efficacy (PMID: 26403509, 26735141). We agree with the reviewer that this does not preclude its repurposing, but the potential for clinical use of this drug is challenged by the failure of this drug over the past decade towards an approved clinical use even for an acute treatment while still lacking any safety and efficacy data for chronic long-term clinical use. We have discussed these limitations in the revised manuscript and have also included discussion of an alternate GSK3 inhibitor - Tideglusib. Unlike LY2090314, Tideglusib irreversibly inhibits GSK-3 (PMID: 22102280) and has been used for long-term dosing in phase II human clinical trials for progressive supranuclear palsy (NCT01049399), Alzheimer's (NCT01350362, NCT00948259), Cardiomyopathy (NCT06174220), and for muscle diseases where it has not only shown efficacy in preclinical use for DMD and myotonic dystrophy type 1 (DM1), it is in phase III clinical testing for the treatment of pediatric and adult DM1 patients (PMID: 31383751, 39587049, 32942085; NCT05004129, NCT02858908, NCT03692312).

The MOA data for these compounds inhibiting FAP adipogenesis are not presented. It remains unclear whether the tested molecules act on the pathways identified as differentially expressed by the authors. Although this referee acknowledges the inherent difficulty in validating mechanisms associated with poorly specific HDAC inhibitors, recent evidence has shown that LY2090314 operates through two complementary circuits, both converging on β-catenin: one mediated by GSK3 inhibition and the other by the stimulation of WNT5A expression. Adding some of these mechanistic information would strength the quality of the work.

Response: As discussed in the response above, to address the mode of LY2090314-mediated inhibition of FAP adipogenesis we first analyzed the level and phosphorylation of β-Catenin. Overall, our new data supports a model in which FAPs from dysferlin deficient muscle show a dysfunctional canonical Wnt signaling characterized by no change in β-catenin level but a reduction of active β-catenin with reduced inhibitory (Ser 9) phosphorylation of GSK-3. This leads to a compensatory upregulation of multiple Wnt ligands including *Wnt 1*, *Wnt 5b*, and *Wnt 10a*. This dysregulated signaling is associated with the increased level of PPARγ, which is consistent with the reduced canonical Wnt signaling, and no change in non-canonical signaling as indicated by unchanged c-JUN phosphorylation. Treatment with LY2090314 reverses key aspects of this imbalance by decreasing p-Tyr216-Tyr279-GSK3 to restore active β-catenin levels, causing reactivation of canonical Wnt signaling through a Ser9-independent mechanism. Accordingly, the drug treatment suppressed the disease-associated upregulation of WNT ligands, indicating normalization of upstream compensatory feedback upon restoration of the downstream GSK-3 signaling, leading to reduced PPARγ levels to inhibit adipogenesis. We have now included this data in a new figure in the revised manuscript (**Fig. 8**).

Minor

Figure 1A. the B2 label is unreadable

Response: We have modified the figure to address this issue.

PDGFRa downregulation during adipogenesis has been demonstrated to occur in FAP cells using mass cytometry. The references Gao et al and Vaziri et al are too general since reported to adipose progenitor cells that are not muscle FAPs. These should be implemented to include a field-relevant bibliographic support.

Response: We have added the citation for the original work (PMID: 20081842) that identified the adipogenic potential of FAPs and demonstrated that, during in vitro adipogenesis, these cells showed increased PPARγ expression and decreased PDGFRα expression, and was validated by an independent study (PMID: 31434718).

Referee #2 (Comments on Novelty/Model System for Author):
Technical quality: in MRI and histology is high. It is low in the pharmacological experiments performed on FAPs. A single lipid stain and missing quantification in Fig. 7G does not suffice. So, overall, I consider it "medium"
Novelty: The paraxial fatty degeneration of muscle in muscular dystrophies has been described several times before. Unfortunately, the exciting finding shown in Fig. 7G has not been elaborated on further, the overall novelty is low.
Medical impact: If the preliminary findings shown in Fig. 7G are substantiated further in in vivo experiments the impact would be high. As shown here, the impact is low to medium.
Model system: The MRI techniques are of very high quality, as are the histological analyses.
Response: We thank the referee for the overall recognition of the strengths of the work and have noted below the changes we have provided additional data and discussion towards the suggested improvements. These are described in our detailed, point-by-point responses below.

Referee #2 (Remarks for Author):
Moon et al. provide a thorough description of MRI findings of paravertebral adipose tissue in one form of limb girdle muscular dystrophy, named dysferlinopathy or LGMD2A/R2. Dysferlinopathy is caused by mutations in the DYSF gene and follows autosomal-recessive inheritance. Correlation of MRI analysis with histological features is demonstrated at different ages in a well-known mouse model of dysferlinopathy (BLA/J).
In addition, MRI data from paraxial and thigh-muscles of 20 patients with LGMDR2 are shown. The extent of paraspinal fatty degeneration of muscle is as severe as in mice.
The authors demonstrate the in vitro reduction of lipid droplets in mouse FAPs by pharmacological intervention, the most efficient medication being the GSK3 inhibitor LY2090314.
Response: We appreciate the reviewer's positive feedback and valuable suggestions for further strengthening the impact of this work.

Major questions:
With a mouse model in place in addition to functional outcome measures on a treadmill, MRI- and histological/immunofluorescent read-outs, the authors should perform in vivo pharmacological treatments. In Figure 7G, an interesting preliminary observation is depicted which should and can be substantiated much further.
Response: We agree with the reviewer that in vivo evidence to test the therapeutic potential of targeting Wnt/B-catenin pathway would enhance the impact of our findings here. Therefore, we tested the *in vivo* impact of LY2090314 treatment on injury-induced adipogenic muscle loss. We treated BLA/J mice with LY2090314 on alternate days for 6 weeks and found that treatment reduced adipogenic degeneration of spinal muscle compared with placebo-treated control mice. These results are presented in the revised manuscript (**Fig. 9, Fig. S3**). To determine whether the effect of LY2090314 on adipogenic muscle loss is mediated through inhibition of injury-triggered adipogenic fate specification in BLA/J FAPs, we monitored the improvement in spinal muscle pathology as well as performed focal limb muscle injury to assess reduction in limb muscle adipogenesis following injury. We observed robust adipogenesis of the spinal and injured limb (TA) muscles in untreated BLA/J mice which was significantly reduced in LY2090314-treated mice. Together, the in vivo studies take the preliminary findings in **Fig. 7G** and establish its in vivo therapeutic potential for preventing dysferlinopathic muscle loss.

It is known that paraxial muscles are significantly affected in a number of muscular dystrophies. The authors do not show comparisons between LGMDR2 and other dystrophies but cite available literature. It would be important to show which aspect of the distribution of fatty replacement of dysferlin-deficient muscle or which pharmacological intervention is specific or helps in the understanding of the underlying mechanistic

pathology. This information is missing. If an intervention could be demonstrated that reverses several adipogenic tissue replacements in muscular dystrophies, this would be of very high impact.

Response: Our new data in **Fig. 8** examining the Wnt/ß-catenin pathway provides a deeper understanding of the underlying mechanistic pathology associated with the adipogenic muscle loss in dysferlinopathy. Further, based on the new experiments we have conducted with the data presented in **Fig. 9** of the revised manuscript, we show the *in vivo* efficacy of this drug treatment and the relevance of the underlying biochemical mechanism in preventing adipogenic muscle degeneration *in vivo*, providing the high impact data alluded by the reviewer. We also agree with the reviewer that comparative cross-disease MRI analyses are important for distinguishing shared versus disease-specific imaging features among dystrophic myopathies. A recent study developed MRI-based criteria to differentiate dysferlinopathy from other genetic muscle diseases (PMID: 38007344); however, this analysis was limited to limb muscles and did not assess spinal/paraxial muscle involvement. Although paraxial muscle involvement has been described in multiple muscular dystrophies, our study was not designed as a direct comparative analysis between LGMDR2 and other dystrophies. We have therefore expanded the Discussion to include this study and, in light of our findings, emphasized the need for future comparative MRI analyses that incorporate spinal muscles to better define imaging features unique to dysferlinopathy versus those shared across dystrophic myopathies.

Minor questions:

Fig. 1: Patient MRIs: Please specify the exact height of the demonstrated sections. Lumbar spine is an area of > 20 cm in lengths in humans.

Response: The sections here are from the axial MRI images of the disc space of L3-4 or the superior endplate of L4, which is now described in the legend for **Fig. 1**.

Fig. 2: The quantification of data is difficult to understand. Fig. 2H - as an example: How can a quotient between fat in paraxial muscle and quadriceps be transformed into %? In addition: May spine and quadriceps muscle be mixed up?

Response: Thank you for pointing out the parts that were difficult to understand. The muscle region was defined based on the deep fascia observed on T1-weighted MRI surrounding the muscle (spinal or quadriceps). This (total) area was set to 100%, and the area covered by fat within each muscle was then expressed as a percentage of the total area for that muscle. **Fig. 2H** shows this ratio as the fat % in the spinal muscle and the fat% in the quadriceps. We thank the reviewer for catching the mislabeling of the two muscle groups, which we have corrected, and added the above description of the quantitative approach in the Methods section.

Fig. 7G: What does n= 4 mean? Four different biological samples? Four different areas of which photos were taken? Technical replicates? Please specify

Response: The data represents 4 independent coverslips, which is now stated in the revised figure legend.

Table 1: Please be consistent in the mutation information whether the exon information should be included or not.

Response: We have provided an updated table with to harmonize all the entries.

Referee #3 (Comments on Novelty/Model System for Author):
Relevant models used, including both human and mouse studies.

Referee #3 (Remarks for Author):

The authors present a thorough and interesting study with relevance to the dysferlin field. Clinical observations in humans with supporting functional studies in mice provides compelling evidence of early-onset spinal muscle degeneration as a feature of dysferlinopathy and identifies promising therapeutic strategies.
Response: We thank the reviewer for recognizing the relevance and the promise of this work, and the valuable suggestions. We have used these to revise the work as described in the point-by-point responses below.

Some minor points for revision:
1. Introduction: Specify which dysferlin mouse model is being referred to.
Response: Thie mouse used in this study is the B6.A-*Dysf*^{prmd}/GeneJ or the Bl/AJ model, which is noted in the method section (Mouse Model) of the manuscript.

2. Page 7 results Figure 1D & E labelling these images with which patient number they are would help with clarity for in-text reference to patient 4.
Response: Patient in Fig. 1D is Patient #2 and in Fig. 1E is Patient #4 from the table and these details are listed in the figure legend.

3. Results page 7 Figure 1/Supp figure 1 "However, irrespective of how widespread fatty degeneration was, the extent of signal in the spinal muscles was higher than the girdle muscles." While this is generally true, there are some patients (9, 13, 18) who have widespread high signal intensity across all muscles.
Response: The reviewer is correct in noting the variability in the signal intensity across muscles. In fact, the extent of signal in the spinal muscles was in most cases higher in the girdle muscles, except for patients 9,13, 18 who have severe disability. To address this comment, we have included the extent of patient's disability in the revised table noting the patients that were wheelchair bound or not at the last known MRI.

4. Results page 8, Figure 3 "mice showed increased stride length, stride duration, stance duration, and swing duration" swing duration was only statistically significantly increased in mice on the decline, not incline.
Response: We have corrected this and to clarify that increased swing duration was only observed during the decline motion.

5. Results page 8 "As the treadmill was maintained at a constant speed (15 cm/s), these delays in locomotory parameters in BLA/J mice were matched by a concomitant reduction in their forelimb and hindlimb stride frequency" is not clear to me what delays in locomotory parameters refers to in regard to the results presented
Response: The locomotory parameters referred to are the altered stride length and duration as well as stance duration. To clarify this, we have reworded this sentence as follows "Since the treadmill speed was fixed at 15 cm/s, these changes meant the mice stepped less often with both their front and back legs."

6. Figure 4A zoomed in regions are taken from different areas for different time points. Given the variability observed between different spinal muscles, this makes it more difficult to directly compare these zoomed in images
Response: The enlarged image in **Fig. 4A** shows the most severe degenerative lesion of a similar location in the iliocostalis, but at a different time point.

7. Figure 7A: One WT sample is a clear outlier on PCA. Was this sample included in the analyses and could the authors comment on this either way?
Response: Yes, this sample was included in the analysis. As in case of human patients, BLA/J mice also exhibit variability of disease severity across mice, thus to ensure the robustness of our analysis, we feel inclusion of all samples is beneficial.

8. The evidence presents exciting possibilities for potential therapeutic approaches for dysferlinopathies. However, as the spinal adipogenesis appears to be an early event, can the authors comment on the possible therapeutic window for these treatments, especially considering the timeline of diagnosis for many dysferlin patients?

Response: The reviewer is correct in noting the need to discuss the issue of therapeutic window, which was not discussed in the original manuscript. We have included a discussion of this topic in the revised manuscript.

9. Could the authors comment on other studies that have also reported fatty accumulations in dysferlin deficient spinal muscles including Agarwal et al doi.org/10.1194/jlr.RA119000399 and Yasa et al doi.org/10.1186/s40478-022-01473-x

Response: We have now included the findings from these studies and cited them in the revised manuscript.

10. The manuscript is generally well written and easy to read but there a few minor text edits required. For example:
Page 3: and maintain calcium homeostasis
Page 3: anti-inflammatory corticosteroids or immunosuppressant
Page 9: Hemotoxylin and eosin staining of revealed
Page 9: Compared to iliocostalis, longissimus, and multifidus muscles showed relatively limited signs of myofiber damage and adipogenic degeneration of the multifidus muscles by 10 months of age. Sentence is unclear
Page 11: has helped tackled this question
Page 11: more stable erector spinae than biped human
Page 13: FAPs in these dysferlinopathic muscle niche that leads to greater adipogenic commitment of FAPs from the spinal muscles. Sentence is unclear

Response: We have made the text edits to correct/clarify these sentences.

1st Jul 2026

Dear Prof. Jaiswal,

Thank you for the submission of your revised manuscript to EMBO Molecular Medicine. I am pleased to inform you that we will be able to accept your manuscript pending the following final amendments:

1) Please consider the referee #1 reference suggestion.
2) Authors: We note name discrepancy Isabelle Ackermann-Bonan in the manuscript and Isabelle Ackermann in our system. Please correct as appropriate.
3) Please consider revising the title to better highlight the main findings, e.g. "GSK3 inhibition ameliorates FAP-driven spinal muscle degeneration in dysferlinopathy"
4) Abstract: I have gone through your text and revised it (see below). Please review it and amend as you see fit:

Dysferlinopathy (Limb Girdle Muscular Dystrophy R2/LGMD2B) is characterized by progressive weakness of limb-girdle muscles. Although gait abnormalities are common, the contribution of spinal muscle pathology to disease progression remains poorly understood. Using cross-sectional MRI in patients and longitudinal analyses in a dysferlinopathic mouse model, we identify early-onset, progressive adipogenic degeneration of spinal muscles as a previously unrecognized hallmark of dysferlinopathy. Multiple spinal muscles, including the iliocostalis, longissimus, and spinalis, are affected, with degeneration preceding pathology in limb muscles. In mice, early spinal muscle loss is associated with altered posture and gait, implicating axial muscle degeneration as a functional driver of disease. Mechanistically, spinal muscle degeneration is accompanied by an intrinsic increase in adipogenic differentiation of spinal fibroadipogenic progenitors (FAPs). Transcriptomic profiling implicated HDAC and GSK3 signaling as main regulators of this adipogenic shift. Pharmacological inhibition of either pathway suppressed adipogenic differentiation of dysferlinopathic FAPs in vitro, while GSK3 inhibition in vivo reduced adipogenic degeneration in spinal and injured limb muscles. These findings establish early spinal muscle degeneration as a key pathological feature of dysferlinopathy and identify GSK3 signaling as a promising therapeutic target.

5) Author checklist: Please submit a complete checklist. https://www.embopress.org/pb-assets/embo-site/EMBO%20Press%20Author%20Checklist-1642513524327.xlsx
6) Figures: Please upload supplementary figures as separate, high resulotion figure files and rename them to Figure EV1 - Figure EV3. The legends should be added to the main manuscript file, after the main figure legends and Table 1, under the heading "Expanded View Figure Legends". Please update their callouts in the text. or more information on figure presentation please check "Author Guidelines". https://www.embopress.org/page/journal/17574684/authorguide#datapresentationformat
7) In the main manuscript file, please do the following:
- Please address all comments suggested by our data editors listed below:
o Figure legends:
1. Please note that the legends for figure 2 are not provided in the sequential manner (legend for figure 2D is provided before legend of figure 2C;). This needs to be rectified.
2. Please note that the exact p values are not provided in the legends of 2F,G,H; 3D-G,K-O; 5E,F; 6C,E; 7H; 8B,D; 9D,G; Supplementary Figure 2-D,K,M.
3. Please indicate the statistical test used for data analysis in the legends of figures 1G,H; 7B.
4. Please note that information related to n is missing in the legends Supplementary Figure 2-J,K,L,M.
5. Please note that the scale bar needs to be defined for figures Supplementary Figure 3-A,B.
6. Please note that scale bar and its definition are missing for figures Supplementary Figure 3-C..
- The manuscript sections should be in the following order: Title page - Abstract - Introduction - Results - Discussion - Methods - Data Availability - Acknowledgments - Disclosure and Competing Interests Statement - References - Figure Legends - Main Tables with legends - Expanded View Figure Legends.
- Add callouts for Fig 3B, E, I-K, N, O.
- In Methods, provide the statement that informed consent was obtained from all human subjects and confirm that the experiments conformed to the principles set out in the WMA Declaration of Helsinki and the Department of Health and Human Services Belmont Report.
- In Methods, a statistical paragraph should reflect all information that you have filled in the Authors Checklist, especially regarding randomization, blinding, replication.
- Indicate in legends exact n and exact p values, not a range, along with the statistical test used. To keep the figures "clear" some authors found providing an Appendix table Sx with all exact p-values preferable. You are welcome to do this if you want to.
- Please add "Disclosure Statement & Competing Interests". We updated our journal's competing interests policy in January 2022 and request authors to consider both actual and perceived competing interests. Please review the policy https://www.embopress.org/competing-interests and update your competing interests if necessary.
- Author contributions: Please remove it from the manuscript and specify author contributions in our submission system. CRediT has replaced the traditional author contributions section because it offers a systematic machine-readable author contributions format that allows for more effective research assessment. You are encouraged to use the free text boxes beneath each contributing author's name to add specific details on the author's contribution. More information is available in our guide to

authors:
https://www.embopress.org/page/journal/17574684/authorguide#authorshipguidelines
- In data availability statement please use the following format to report the accession number of your data:

The datasets produced in this study are available in the following databases:
[data type]: [full name of the resource] [accession number/identifier] ([doi or URL or identifiers.org/DATABASE:ACCESSION])

Please check "Author Guidelines" for more information.
https://www.embopress.org/page/journal/17574684/authorguide#availabilityofpublishedmaterial
8) Tables: Rename Suppl. Table 1 to Table EV1, upload it as a separate file with its legend in the file. Please update the callout in the main text.
9) Reagent Table: Please rename the file to Reagent Table. More information on how to adhere to this format as well as downloadable templates (.docx) for the Reagents and Tools Table can be found in our author guidelines:
https://www.embopress.org/page/journal/17574684/authorguide#structuredmethods
10) Funding: Please make sure that information about all sources of funding are complete in both our submission system and in the manuscript. Currently, the Jain Foundation, Children's National Hospital, RS- 2023-00236157, Ministry of Health & Welfare of Korea (HR22C1832), research funds of Jeonbuk National University, and Biomedical Research Institute of Jeonbuk National University is missing in our submission system. Please correct.
11) The Paper Explained: Please provide "The Paper Explained" and add it to the main manuscript text. Please check any of recently published EMBO Mol Med research articles for reference.
12) Synopsis: Every published paper now includes a 'Synopsis' to further enhance discoverability. Synopses are displayed on the journal webpage and are freely accessible to all readers. They include separate synopsis image and synopsis text.
- Synopsis image: Please provide a visual abstract as a high-resolution jpeg file exactly 550 px-wide x 300 pixels high to illustrate your article.
- Synopsis text: Please provide a short standfirst (maximum of 300 characters, including space) as well as 2-5 one sentence bullet points that summarise the paper as a .doc file. Please write the bullet points to summarise the key NEW findings. They should be designed to be complementary to the abstract - i.e. not repeat the same text. We encourage inclusion of key acronyms and quantitative information (maximum of 30 words / bullet point). Please use the passive voice.
- Please check your synopsis text and image before submission with your revised manuscript. Please be aware that in the proof stage minor corrections only are allowed (e.g., typos).
13) As part of the EMBO Publications transparent editorial process (see our Editorial at http://embomolmed.embopress.org/content/2/9/329), EMBO Molecular Medicine will publish online a Review Process File (RPF) to accompany accepted manuscripts. This file will be published in conjunction with your paper and will include the anonymous referee reports, your point-by-point response and all pertinent correspondence relating to the manuscript. Let us know if you want to remove or not any figures from it prior to publication. Please note that the Authors checklist will be published at the end of the RPF.
14) Please provide a point-by-point letter INCLUDING my comments as well as the reviewer's reports and your detailed responses (as Word file).

I look forward to reading a new revised version of your manuscript as soon as possible.

Yours sincerely,

Zeljko Durdevic

Zeljko Durdevic
Senior Editor
EMBO Molecular Medicine

*** Instructions to submit your revised manuscript ***

*** PLEASE NOTE *** As part of the EMBO Publications transparent editorial process initiative (see our Editorial at https://www.embopress.org/doi/pdf/10.1002/emmm.201000094), EMBO Molecular Medicine will publish online a Review Process File to accompany accepted manuscripts.

In the event of acceptance, this file will be published in conjunction with your paper and will include the anonymous referee reports, your point-by-point response and all pertinent correspondence relating to the manuscript. If you do NOT want this file to be published, please inform the editorial office at contact@embomolmed.org.

*****

When preparing your revised manuscript, please refer to our guidelines: https://link.springer.com/journal/44321/submission-guidelines#cms-Revised-submissions. We perform an initial quality control of all revised manuscripts before re-review; failure to include requested items will delay the evaluation of your revision.

We require:

1) A .docx formatted version of the manuscript text (including legends for main figures, EV figures and tables). Please make sure that the changes are highlighted to be clearly visible.

2) Individual production quality figure files as .eps, .tif, .jpg (one file per figure). For guidance, download the 'Figure Guide PDF': https://media.springernature.com/original/springer-cms/rest/v1/content/27825798/data/v1.

3) A .docx formatted letter INCLUDING the reviewers' reports and your detailed point-by-point responses to their comments. As part of the EMBO Press transparent editorial process, the point-by-point response is part of the Review Process File (RPF), which will be published alongside your paper.

4) A complete author checklist, which you can download from our author guidelines. Please insert information in the checklist that is also reflected in the manuscript. The completed author checklist will also be part of the RPF.

5) Please note that all corresponding authors are required to supply an ORCID ID for their name upon submission of a revised manuscript.

6) It is mandatory to include a 'Data Availability' section after the Materials and Methods. Before submitting your revision, primary datasets produced in this study need to be deposited in an appropriate public database, and the accession numbers and database listed under 'Data Availability'. Please remember to provide a reviewer password if the datasets are not yet public.

In case you have no data that requires deposition in a public database, please state so in this section. Note that the Data Availability Section is restricted to new primary data that are part of this study.

7) For data quantification: please specify the name of the statistical test used to generate error bars and P values, the number (n) of independent experiments (specify technical or biological replicates) underlying each data point and the test used to calculate p-values in each figure legend. The figure legends should contain a basic description of n, P and the test applied. Graphs must include a description of the bars and the error bars (s.d., s.e.m.).

8) At EMBO Press we ask authors to provide source data for the main manuscript figures. You will receive a separate email with instructions for providing source data with your revised manuscript, including how to upload and organize the files.

9) Our journal encourages inclusion of *data citations in the reference list* to directly cite datasets that were re-used and obtained from public databases. Data citations in the article text are distinct from normal bibliographical citations and should directly link to the database records from which the data can be accessed. In the main text, data citations are formatted as follows: "Data ref: Smith et al, 2001" or "Data ref: NCBI Sequence Read Archive PRJNA342805, 2017". In the Reference list, data citations must be labeled with "[DATASET]". A data reference must provide the database name, accession number/identifiers and a resolvable link to the landing page from which the data can be accessed at the end of the reference.

10) We replaced Supplementary Information with Expanded View (EV) Figures and Tables that are collapsible/expandable online. A maximum of 5 EV Figures can be typeset. EV Figures should be cited as 'Figure EV1, Figure EV2" etc... in the text and their respective legends should be included in the main text after the legends of regular figures.

- For the figures that you do NOT wish to display as Expanded View figures, they should be bundled together with their legends in a single PDF file called *Appendix*, which should start with a short Table of Content. Appendix figures should be referred to in the main text as: "Appendix Figure S1, Appendix Figure S2" etc.

- Additional Tables/Datasets should be labeled and referred to as Table EV1, Dataset EV1, etc. Legends have to be provided in a separate tab in case of .xls files. Alternatively, the legend can be supplied as a separate text file (README) and zipped together with the Table/Dataset file.

11) The paper explained: EMBO Molecular Medicine articles are accompanied by a summary of the articles to emphasize the

major findings in the paper and their medical implications for the non-specialist reader. Please provide a draft summary of your article highlighting

- the medical issue you are addressing,

- the results obtained and

- their clinical impact.

This may be edited to ensure that readers understand the significance and context of the research. Please refer to any of our published articles for an example.

12) Author contributions: You will be asked to provide CRediT (Contributor Role Taxonomy) terms in the submission system. These replace a narrative author contribution section in the manuscript.

13) A Conflict of Interest statement should be provided in the main text.

14) Every published paper includes a 'Synopsis' to further enhance discoverability. Synopses are displayed on the journal webpage and are freely accessible to all readers. They include a short stand first (maximum of 300 characters, including space) as well as 2-5 one-sentences bullet points that summarizes the paper. Please write the bullet points to summarize the key NEW findings. They should be designed to be complementary to the abstract - i.e. not repeat the same text. We encourage inclusion of key acronyms and quantitative information (maximum of 30 words / bullet point). Please use the passive voice. Please attach these in a separate file or send them by email, we will incorporate them accordingly.

Please also suggest a striking image or visual abstract to illustrate your article as a PNG file 550 px wide x 300 px high.

15) Include a Reagents and Tools Table as part of the Methods section, which can be downloaded from our author guidelines.

EMBO Molecular Medicine has a "scooping protection" policy, whereby similar findings that are published by others during review or revision are not a criterion for rejection. Should you decide to submit a revised version, I do ask that you get in touch after three months if you have not completed it, to update us on the status.

*Additional important information regarding Figures

Each figure should be given in a separate file and should have the following resolution:
Graphs 800-1,200 DPI
Photos 400-800 DPI
Colour (only CMYK) 300-400 DPI"

Figures are not edited by the production team. All lettering should be the same size and style; figure panels should be indicated by capital letters (A, B, C etc). Gridlines are not allowed except for log plots. Figures should be numbered in the order of their appearance in the text with Arabic numerals. Each Figure must have a separate legend and a caption is needed for each panel.

*Additional important information regarding figures and illustrations can be found at
https://media.springernature.com/original/springer-cms/rest/v1/content/27825798/data/v1

***** Reviewer's comments *****

Referee #1 (Comments on Novelty/Model System for Author):

The experimental design and the findings demonstrating the ability of LY2090314 are not novel and highly overlaps with the original papers demonstrating the ability of this compound to mitigate intramuscular fat deposition in other models of muscle wasting disease. Despite this, this work is valid and merit publication.

Referee #1 (Remarks for Author):

The manuscript has been substantially improved and all the concerns raised by this referee have been adequately addressed. The referee kindly suggests considering the inclusion of the following relevant reference among those cited in the final manuscript: PMID: 37272428. This study demonstrated that inhibition of GSK3 effectively blocks adipogenic differentiation of human FAPs, thereby providing additional evidence supporting the rationale for the use of GSK3 inhibitors as a strategy to limit intramuscular fat infiltration. Incorporating this work would further strengthen the discussion and contextualization of the present findings.

Referee #3 (Comments on Novelty/Model System for Author):

The authors have addressed the issues and concerns raised in my original review. The additional data and revised text strengthens and clarifies the manuscript which is now suitable for publication in my opinion. The in vivo testing of LY2090314 is promising, but side effects require further investigation which are outside the scope of the current work. This work will provide important information for the field.

Referee #3 (Remarks for Author):

The additional work has now improved this manuscript for publication and I look forward to seeing the next steps in this research.

<u>Response to Reviewer Comments</u>

Referee #1 (Comments on Novelty/Model System for Author):

The experimental design and the findings demonstrating the ability of LY2090314 are not novel and highly overlaps with the original papers demonstrating the ability of this compound to mitigate intramuscular fat deposition in other models of muscle wasting disease. Despite this, this work is valid and merit publication.

Response: The experiments conducted here establish the role of Wnt signaling dysregulation in adipogenic fate of FAPs from dysferlinopathic mouse spinal muscle and establishes that GSK3 inhibition inhibits this. These are novel findings for this disease and we thank the reviewer for recognizing the merit of this work for publication.

Referee #1 (Remarks for Author):

The manuscript has been substantially improved and all the concerns raised by this referee have been adequately addressed.

The referee kindly suggests considering the inclusion of the following relevant reference among those cited in the final manuscript: PMID: 37272428. This study demonstrated that inhibition of GSK3 effectively blocks adipogenic differentiation of human FAPs, thereby providing additional evidence supporting the rationale for the use of GSK3 inhibitors as a strategy to limit intramuscular fat infiltration. Incorporating this work would further strengthen the discussion and contextualization of the present findings.

Response: This reference suggested by the reviewer has been added to the discussion section.

21st Aug 2026

Dear Prof. Jaiswal,

We are pleased to inform you that your manuscript is accepted for publication and is now being sent to our publisher to be included in the next available issue of EMBO Molecular Medicine.

Your manuscript will be processed for publication by EMBO Press. It will be copy edited and you will receive page proofs prior to publication. Please note that you will be contacted by Springer Nature Author Services to complete licensing and payment information.

You may qualify for financial assistance for your publication charges - either via a Springer Nature fully open access agreement or an EMBO initiative. Check your eligibility: https://link.springer.com/journal/44321/how-to-publish-with-us

Should you be planning a Press Release on your article, please notify the production team via this form in order to coordinate publication and release dates: https://forms.cloud.microsoft/e/eH4A8JVP6g

If you have any questions, please do not hesitate to contact the Editorial Office. Thank you for your contribution to EMBO Molecular Medicine.

Yours sincerely,
Zeljko Durdevic

Zeljko Durdevic
Senior Editor
EMBO Molecular Medicine

>>> Please note that it is EMBO Molecular Medicine policy for the transcript of the editorial process (containing referee reports and your response letter) to be published as an online supplement to each paper. If you do NOT want this, you will need to inform the Editorial Office via email immediately. More information is available here: https://link.springer.com/partners/embo-press/editorial-policies#Peer%20review

EMBO Press Author Checklist

Corresponding Author Name: Jyoti K. Jaiswal	
Journal Submitted to: EMBO Molecular Medicine	
Manuscript Number: EMM-2025-22753-V2	

USEFUL LINKS FOR COMPLETING THIS FORM
The EMBO Journal - Author Guidelines
EMBO Reports - Author Guidelines
Molecular Systems Biology - Author Guidelines
EMBO Molecular Medicine - Author Guidelines

Reporting Checklist for Life Science Articles (updated January
This checklist is adapted from Materials Design Analysis Reporting (MDAR) Checklist for Authors. MDAR establishes a minimum set of requirements in transparent reporting in the life sciences (see Statement of Task: 10.31222/osf.io/9sm4x). Please follow the journal's guidelines in preparing your
Please note that a copy of this checklist will be published alongside your article.

Abridged guidelines for figures
1. Data
The data shown in figures should satisfy the following conditions:
- the data were obtained and processed according to the field's best practice and are presented to reflect the results of the experiments in an accurate and unbiased manner.
- ideally, figure panels should include only measurements that are directly comparable to each other and obtained with the same assay.
- plots include clearly labeled error bars for independent experiments and sample sizes. Unless justified, error bars should not be shown for technical
- if n<5, the individual data points from each experiment should be plotted. Any statistical test employed should be justified.
- Source Data should be included to report the data underlying figures according to the guidelines set out in the authorship guidelines on Data

2. Captions
Each figure caption should contain the following information, for each panel where they are relevant:
- a specification of the experimental system investigated (eg cell line, species name).
- the assay(s) and method(s) used to carry out the reported observations and measurements.
- an explicit mention of the biological and chemical entity(ies) that are being measured.
- an explicit mention of the biological and chemical entity(ies) that are altered/varied/perturbed in a controlled manner.
- the exact sample size (n) for each experimental group/condition, given as a number, not a range;
- a description of the sample collection allowing the reader to understand whether the samples represent technical or biological replicates (including how many animals, litters, cultures, etc.).
- a statement of how many times the experiment shown was independently replicated in the laboratory.
- definitions of statistical methods and measures:
  - common tests, such as t-test (please specify whether paired vs. unpaired), simple χ2 tests, Wilcoxon and Mann-Whitney tests, can be unambiguously identified by name only, but more complex techniques should be described in the methods section;
  - are tests one-sided or two-sided?
  - are there adjustments for multiple comparisons?
  - exact statistical test results, e.g., P values = x but not P values < x;
  - definition of 'center values' as median or average;
  - definition of error bars as s.d. or s.e.m.

Please complete ALL of the questions below.
Select "Not Applicable" only when the requested information is not relevant for your study.

Materials

Newly Created Materials	Information included in the manuscript?	In which section is the information available? (Reagents and Tools Table, Materials and Methods, Figures, Data Availability Section)
New materials and reagents need to be available; do any restrictions apply?	Not Applicable	

Antibodies	Information included in the manuscript?	In which section is the information available? (Reagents and Tools Table, Materials and Methods, Figures, Data Availability Section)
For **antibodies** provide the following information:<br>- Commercial antibodies: RRID (if possible) or supplier name, catalogue number and or/clone number<br>- Non-commercial: RRID or citation	Yes	Reagent Table

DNA and RNA sequences	Information included in the manuscript?	In which section is the information available? (Reagents and Tools Table, Materials and Methods, Figures, Data Availability Section)
**Short novel DNA or RNA including primers, probes**: provide the sequences.	Yes	Table EV1

Cell materials	Information included in the manuscript?	In which section is the information available? (Reagents and Tools Table, Materials and Methods, Figures, Data Availability Section)
**Cell lines:** Provide species information, strain. Provide accession number in repository **OR** supplier name, catalog number, clone number, and/**OR** RRID.	Not Applicable	
**Primary cultures:** Provide species, strain, sex of origin, genetic modification status.	Yes	Methods
Report if the cell lines were recently **authenticated** (e.g., by STR profiling) and tested for mycoplasma contamination.	Not Applicable	

Experimental animals	Information included in the manuscript?	In which section is the information available? (Reagents and Tools Table, Materials and Methods, Figures, Data Availability Section)
**Laboratory animals or Model organisms:** Provide species, strain, sex, age, genetic modification status. Provide accession number in repository **OR** supplier name, catalog number, clone number, **OR** RRID.	Yes	Methods, Reagent Table
**Animal observed in or captured from the field:** Provide species, sex, and age where possible.	Not Applicable	
Please detail **housing and husbandry conditions**.	Yes	Methods

Plants and microbes	Information included in the manuscript?	In which section is the information available? (Reagents and Tools Table, Materials and Methods, Figures, Data Availability Section)
**Plants:** provide species and strain, ecotype and cultivar where relevant, unique accession number if available, and source (including location for collected wild specimens).	Not Applicable	
**Microbes:** provide species and strain, unique accession number if available, and source.	Not Applicable	

Human research participants	Information included in the manuscript?	In which section is the information available? (Reagents and Tools Table, Materials and Methods, Figures, Data Availability Section)
If collected and within the bounds of privacy constraints report on age, sex and gender or ethnicity for all study participants.	Yes	Table 1

Core facilities	Information included in the manuscript?	In which section is the information available? (Reagents and Tools Table, Materials and Methods, Figures, Data Availability Section)
If your work benefited from core facilities, was their service mentioned in the acknowledgments section?	Yes	Acknowledgements

Design

Study protocol	Information included in the manuscript?	In which section is the information available? (Reagents and Tools Table, Materials and Methods, Figures, Data Availability Section)
If study protocol has been **pre-registered, provide DOI in the manuscript**. For clinical trials, provide the trial registration number **OR** cite DOI.	Not Applicable	
Report the **clinical trial registration number** (at ClinicalTrials.gov or equivalent), where applicable.	Yes	Methods

Laboratory protocol	Information included in the manuscript?	In which section is the information available? (Reagents and Tools Table, Materials and Methods, Figures, Data Availability Section)
Provide DOI OR other citation details if **external detailed step-by-step protocols** are available.	Not Applicable	

Experimental study design and statistics	Information included in the manuscript?	In which section is the information available? (Reagents and Tools Table, Materials and Methods, Figures, Data Availability Section)
Include a statement about **sample size** estimate even if no statistical methods were used.	Yes	Methods
Were any steps taken to minimize the effects of subjective bias when allocating animals/samples to treatment (e.g. **randomization procedure**)? If yes, have they been described?	Yes	Methods
Include a statement about **blinding** even if no blinding was done.	Yes	Methods
Describe **inclusion/exclusion criteria** if samples or animals were excluded from the analysis. Were the criteria pre-established? If sample or data points were omitted from analysis, report if this was due to attrition or intentional exclusion and provide justification.	Yes	Methods
For every figure, are **statistical tests** justified as appropriate? Do the data meet the assumptions of the tests (e.g., normal distribution)? Describe any methods used to assess it. Is there an estimate of variation within each group of data? Is the variance similar between the groups that are being statistically compared?	Yes	Methods, Appendix

Sample definition and in-laboratory replication	Information included in the manuscript?	In which section is the information available? (Reagents and Tools Table, Materials and Methods, Figures, Data Availability Section)
In the figure legends: state number of times the experiment was **replicated** in laboratory.	Yes	Appendix
In the figure legends: define whether data describe **technical or biological replicates**.	Yes	Appendix

Ethics

Ethics	Information included in the manuscript?	In which section is the information available? (Reagents and Tools Table, Materials and Methods, Figures, Data Availability Section)
Studies involving **human participants**: State details of **authority granting ethics approval** (IRB or equivalent committee(s), provide reference number for approval.	Yes	Methods
Studies involving **human participants**: Include a statement confirming that **informed consent** was obtained from all subjects and that the experiments conformed to the principles set out in the WMA Declaration of Helsinki and the Department of Health and Human Services Belmont Report.	Yes	Methods
Studies involving **human participants: For publication of patient photos**, include a statement confirming that consent to publish was obtained.	Not Applicable	
Studies involving experimental **animals**: State details of **authority granting ethics approval** (IRB or equivalent committee(s), provide reference number for approval. Include a statement of compliance with ethical regulations.	Yes	Methods
Studies involving **specimen and field samples:** State if relevant **permits** obtained, provide details of authority approving study; if none were required, explain why.	Not Applicable	

Dual Use Research of Concern (DURC)	Information included in the manuscript?	In which section is the information available? (Reagents and Tools Table, Materials and Methods, Figures, Data Availability Section)
Could your study fall under dual use research restrictions? Please check biosecurity documents and list of **select agents and toxins** (CDC): https://www.selectagents.gov/sat/list.htm	Not Applicable	
If you used a select agent, is the security level of the lab appropriate and reported in the manuscript?	Not Applicable	
If a study is subject to dual use research of concern regulations, is the name of the **authority granting approval and reference number** for the regulatory approval provided in the manuscript?	Not Applicable	

Reporting

The MDAR framework recommends adoption of discipline-specific guidelines, established and endorsed through community initiatives. Journals have their own policy about requiring specific guidelines and recommendations to complement MDAR.

Adherence to community standards	Information included in the manuscript?	In which section is the information available? (Reagents and Tools Table, Materials and Methods, Figures, Data Availability Section)
State if relevant guidelines or checklists (e.g., **ICMJE, MIBBI, ARRIVE, PRISMA**) have been followed or provided.	Yes	Methods
For **tumor marker prognostic studies,** we recommend that you follow the **REMARK** reporting guidelines (see link list at top right). See author guidelines, under 'Reporting Guidelines'. Please confirm you have followed these guidelines.	Not Applicable	
For **phase II and III randomized controlled trials**, please refer to the **CONSORT** flow diagram (see link list at top right) and submit the CONSORT checklist (see link list at top right) with your submission. See author guidelines, under 'Reporting Guidelines'. Please confirm you have submitted this list.	Not Applicable	

Data Availability

Data availability	Information included in the manuscript?	In which section is the information available? (Reagents and Tools Table, Materials and Methods, Figures, Data Availability Section)
Have **primary datasets** been deposited according to the journal's guidelines (see 'Data Deposition' section) and the respective accession numbers provided in the Data Availability Section?	Yes	Data Availability
Were **human clinical and genomic datasets** deposited in a public access-controlled repository in accordance to ethical obligations to the patients and to the applicable consent agreement?	Not Applicable	
Are **computational models** that are central and integral to a study available without restrictions in a machine-readable form? Were the relevant accession numbers or links provided?	Not Applicable	
If publicly available data were reused, provide the respective **data citations in the reference list**.	Not Applicable	
