# Supplementary material for: FAP adipogenesis drives early spinal muscle loss in dysferlinopathy and reveals a therapeutic target
Source: EMBO Mol Med. 2026 Oct 12;18(10):4034–58. doi: 10.1038/s44321-026-00514-5 (PMC13658238; doi:10.1038/s44321-026-00514-5)
Supplement: Supplementary file 4 [file 44321_2026_514_MOESM1_ESM.docx]

**Table EV1: The primer sequences used in the qPCR.** Sequence of the gene-specific primer pairs used for qPCR analysis reported in Figure 8.

| **qRT-PCR** | | |
| --- | --- | --- |
| Genes | Forward (5′ to 3′) | Reverse (3′ to 5′) |
| *Wnt1* | ATGAACCTTCACAACAACGAG | GGTTGCTGCCTCGGTTG |
| *Wnt5a* | CTCCTTCGCCCAGGTTGTTATAG | TGTCTTCGCACCTTCTCCAATG |
| *Wnt5b* | ATGCCCGAGAGCGTGAGAAG | ACATTTGCAGGCGACATCAGC |
| *Wnt10a* | CCTGTTCTTCCTACTGCTGCTGG | CGATCTGGATGCCCTGGATAGC |
| *Axin2* | ATGGAGTCCCTCCTTACCGCAT | GTTCCACAGGCGTCATCTCCTT |
| *Ccnd2* | GCAGAAGGACATCCAACCGTAC | ACTCCAGCCAAGAAACGGTCCA |
| *Actb* | CATTGCTGACAGGATGCAGAAGG | TGCTGGAAGGTGGACAGTGAGG |
